# Screening and validation of diagnostic markers for keloids via bioinformatics analysis

**DOI:** 10.1016/j.bbrep.2025.102219

**Published:** 2025-08-22

**Authors:** Ze Wang, Bo Hu, Wenfei Li, Tengxiao Ma, Lei Li

**Affiliations:** Hainan Affiliated Hospital of Hainan Medical University, Hainan, Haikou, 570100, China

**Keywords:** Keloid, Bioinformatics, Machine learning, Diagnostic markers, Immune cell infiltration

## Abstract

**Background:**

Keloid (KD) disease is a skin lesions caused by abnormal wound healing that involve complex cellular and molecular mechanisms. The aim of this study was to screen diagnostic markers of KD via bioinformatics methods and evaluate their clinical application value.

**Methods:**

The GSE44270, GSE145725, GSE7890 and GSE83286 datasets were analyzed in combination with difference analysis and weighted gene coexpression network analysis (WGCNA) and machine learning algorithms, candidate genes related to KD were screened and verified via receiver operating characteristic (ROC) curves and external datasets. KD samples were classified by consistent clustering, and the infiltration of immune cells was investigated. Simultaneously, a diagnostic biomarker-related ceRNA network was constructed. Drug small molecules and compounds were predicted online, and molecular docking was performed. Finally, RT‒qPCR and WB were used to verify the expression of the markers.

**Results:**

In this study, two upregulated genes, SMURF2 and CCDC80, which are significantly associated with a variety of immune cells, were screened. KD was divided into the C1 and C2 subtypes, SMURF2 was highly expressed in C1, and CCDC80 was highly expressed in C2. Drug prediction and molecular docking analysis suggest that bisphenol A may have a potential effect on KD therapy. RT‒qPCR and WB revealed that the mRNA and protein expression levels of SMURF2 and CCDC80 in KD samples were significantly increased.

**Conclusion:**

Our study identified two genes that may be used as diagnostic markers of KD, providing new perspectives and potential molecular targets for the study of the molecular mechanisms and clinical diagnosis of KD.

## Highlights

• SMURF2 and CCDC80 genes may serve as potential diagnostic biomarkers for keloids.
• SMURF2 and CCDC80 genes are correlated with the infiltration of multiple types of immune cells.
• Bisphenol A may have a potential therapeutic effect on keloids.

## 1 Introduction

Keloid (KD) is the result of abnormal wound healing and is often caused by skin damage and stimulation, such as trauma and surgery [1]. KD involves a variety of cell types and complex molecular mechanisms, such as inflammation, cell proliferation, and abnormal deposition of the extracellular matrix [2]. The clinical manifestations are an increased skin surface area, a darker color, and invasive growth into the surrounding tissues [3]. This lesion not only affects the appearance of patients but also leads to an inferiority complex, depression and anxiety in patients and is often accompanied by uncomfortable symptoms such as pruritus and pain, which seriously affect patient quality of life [4]. Epidemiological studies have shown that the prevalence of KD is greater in women and that the incidence is greater in populations with darker skin color, such as Africans and Asians [5]. Currently, the treatment methods for KD include laser therapy, radiation therapy, corticosteroid injection, compression therapy, cryotherapy, and surgical resection [6]. Although these treatment methods can delay the progression of KD, most of them are hindered by low efficacy, high recurrence rates, and long treatment cycles [7]. Although many studies have investigated the pathogenesis of KD, to date, the exact pathophysiology of KD development remains unclear, thus limiting the development of effective treatments. Therefore, an in-depth study of KD has far-reaching significance.

With the development of high-throughput sequencing technology, the application of bioinformatics in genomics, transcriptomics, proteomics and other fields has become increasingly widespread, especially in the identification of disease-associated genes and biomarkers. Bioinformatics has become an indispensable part of modern biological research. This study first used difference analysis and weighted gene coexpression network analysis (WGCNA) to screen key genes and then explored candidate genes with the help of a machine learning algorithm. The expression of the candidate genes was subsequently validated with the help of external datasets to construct the KD diagnostic model. We also investigated immune cell infiltration and analyzed the correlations between diagnostic markers and immune cells. This study performed consensus clustering analysis on KD samples to further investigate the relationships between different subtypes and the immune microenvironment. Prediction of microRNA (miRNA), transcription factor (TF), and long noncoding RNA (lncRNA) biomarkers through online databases, and construction of a complex lncRNA-miRNA-mRNA-TF network. Drug prediction and molecular docking experiments were performed on the diagnostic markers. Finally, the relevant bioinformatics findings were validated via RT‒qPCR and WB experiments. Through the above process, we hope to provide new perspectives for the study of the molecular mechanisms of KD and potential molecular targets for the development of future treatment strategies.

## 2 Materials and methods

### 2.1 Data acquisition

This study downloaded the following datasets from the GEO database (https://www.ncbi.nlm.nih.gov/geo/): GSE44270 [8], GSE145725 [9], GSE7890 [10], and GSE83286 [11]. All included samples did not receive any treatment, and their ids were converted according to the platform files. The details of the datasets are listed in Table 1.

**Table 1:** GEO dataset information.

GEO series	GPL platform	Sequencing type	Molecular type	Species origin	Tissue source	Control case	Keloid case
GSE44270	GPL6244 [HuGene-1_0-st] Affymetrix Human Gene 1.0 ST Array [transcript (gene) version]	microarray	mRNA	Homo sapiens	fibroblasts	7	9
GSE145725	GPL16043 GeneChip® PrimeView™ Human Gene Expression Array (with External spike-in RNAs)	microarray	mRNA	Homo sapiens	fibroblasts	10	9
GSE7890	GPL570 [HG-U133_Plus_2] Affymetrix Human Genome U133 Plus 2.0 Array	microarray	mRNA	Homo sapiens	fibroblasts	5	5
GSE83286	GPL19612 Agilent-062918 OE Human lncRNA Microarray V4.0 028004 [Probe Name version]	microarray	lncRNA	Homo sapiens	skin	3	3

### 2.2 Difference analysis and construction of weighted gene coexpression network analysis (WGCNA)

The limma package in R software (version 4.2.1) was used [12] to identify the differentially expressed genes (DEGs) in the GSE44270 dataset. The screening threshold was set to a |log2-fold change| >0 and a p value < 0.05, and the DEGs were visualized in the form of a volcano map and heatmap. Using the WGCNA package [13], we read, filtered and normalized the GSE145725 dataset and used the goodSamplesGenes function to check and remove the samples and genes with missing values. After the soft threshold was determined via the pickSoftThreshold function, the adjacency matrix was converted to a topological overlap matrix (TOM), hierarchical cluster analysis was subsequently performed on the basis of the TOM, and branches with low heights in the clustering tree were cut off. Next, the dynamic tree cutting method of WGCNA was used to identify gene modules and merge similar modules. The relationships between the modules and the traits were analyzed by calculating the correlations between the module eigengenes (MEs) and the clinical phenotypes (the fit index was greater than 0.8). On this basis, module membership (MM) and gene importance (GS) were calculated and visualized. Finally, the gene list of each module is output.

### 2.3 Functional enrichment analysis of DEGs

The DEGs in the GSE44270 dataset and the significant module genes from the WGCNA of the GSE145725 dataset were intersected and calculated via the VennDiagram package [14]. A Venn diagram was drawn, and the expression of DEGs is shown as a heatmap. The clusterProfiler package [15] was subsequently used to perform Gene Ontology (GO) analysis on the DEGs [16], and the Kyoto Encyclopedia of Genes and Genomes (KEGG) [17] was used for enrichment analysis, which included biological process (BP), cellular component (CC), and molecular function (MF) terms. The results of the GO and KEGG enrichment analyses were visualized via a bubble plot.

### 2.4 Machine learning and intergene correlation analysis

On the basis of the GSE44270 gene expression data, a generalized linear model (GLM) was used, with 70 % for the training set and 30 % for the test set [18]. Four machine learning algorithms, namely, random forest (RF) [19], support vector machine (SVM) [20] and extreme gradient boosting (XGB) [21], were used for model training, and the performance of each model was evaluated on the basis of the prediction results. In the model validation stage, the trained model was used to make predictions on the test set, the ROC curve was drawn, and the area under the curve (AUC) was calculated [22]. Using the DALEX package [23], the importance of genes to the prediction model was calculated, and a feature importance plot was drawn. The residuals of different models were analyzed, and the reverse cumulative distributions of the residual diagram and boxplots of the residual diagram were drawn.

The corrplot package [24] was used to generate a correlation heatmap among the candidates. The gene location information of the chromosomes was subsequently downloaded from the Ensembl database (https://grch37.ensembl.org/index.html), and a chromosome location map of the 10 genes was drawn.

### 2.5 Validation on external datasets and construction and evaluation of diagnostic models

The expression of the candidate genes was validated via two external databases, GSE7890 and GSE145725. A box plot of the expression of the candidate genes was drawn, and the intersection of the significant DEGs was further analyzed. Using the rms package [25] with GSE44270 as the training set, a nomogram of the disease risk prediction model of the intersection genes was constructed. The model was calibrated, and a calibration curve was drawn. The ggDCA package was used [26] to perform decision curve analysis (DCA). The GSE145725 and GSE7890 datasets were used as the validation cohorts. ROC curves were drawn, and AUC values were calculated to evaluate the predictive performance of genes and further evaluate the stability and reliability of the models [27].

### 2.6 Immune infiltration assay

The GSE7890, GSE44270 and GSE145725 datasets were merged [28]. The infiltration of 22 types of immune cells in the KD and control samples was analyzed via the CIBERSORT package. The relative percentages of immune cells in different samples are shown via bar graphs, and the differences in immune cell infiltration are shown via box plots. After the samples from the control group were removed, the correlations between the diagnostic markers and immune cell infiltration were analyzed, and a Lollipop chart was drawn on the basis of the p value and correlation coefficient. In addition, the samples were divided into two groups, high expression and low expression, on the basis of the gene expression levels of diagnostic markers, to explore the effects of differences in the expression levels of diagnostic markers on immune cell infiltration.

### 2.7 Consistency cluster analysis

Using the Consensus Cluster Plus package [29], cluster analysis was performed on the basis of the mRNA expression profiles of the diagnostic markers. The optimal number of clusters was determined on the basis of intragroup consistency. Immune infiltration analysis was performed on the different classifications, and a bar graph and box plot were drawn. Differential expression analysis of different classifications was subsequently performed. The threshold was set to a |log2-fold fold change| > 0.2 and a p value < 0.05. The results were visualized via a volcano map and heatmap. Finally, GO and KEGG enrichment analyses were performed on the DEGs to obtain pathway information at the gene level.

### 2.8 ceRNA network construction

Three databases were used for prediction of the upstream miRNAs of diagnostic markers: miRDB (https://mirdb.org/), miRTarBase (https://mirtarbase.cuhk.edu.cn/), and TargetScan8.0 (https://www.targetscan.org/vert_80/). The ChIPBase (https://rnasysu.com/chipbase3/index.php) database was used to predict the TFs associated with diagnostic markers. Differential analysis of GSE83286 was performed to screen the differentially expressed lncRNAs. Finally, the lncRNAs upstream of the miRNAs were predicted via the miRcode database (http://www.mircode.org/). On the basis of these prediction results, a lncRNA‒miRNA‒mRNA‒TF regulatory network was constructed.

### 2.9 Drug prediction and molecular docking

The CTD database (https://ctdbase.org) was used to predict the compounds or small molecule drugs that may act as biomarkers. The 2D structures of potential active ingredients and marker targets were downloaded from PubChem (https://pubchem.ncbi.nlm.nih.gov/) and the PDB database (http://www.rcsb.org/), respectively, and then an AutoDock Vina (v1.2.2) [30] molecular docking study was performed. Finally, the docking results were visualized and analyzed via PyMOL (v.2.4.0) software.

### 2.10 Reverse transcription quantitative polymerase chain reaction (RT‒qPCR) and western blotting

The specimens were obtained from 3 patients who were diagnosed with keloids in the Department of Plastic Surgery, Hainan General Hospital, in November 2024 and who underwent keloid surgical resection. Another 3 samples of redundant normal skin tissue formed during the closure of incisions in patients who underwent excision of masses during the same period were collected. All patients were fully informed of the study objectives and procedures before surgery, and written informed consent was obtained. The study was approved by the Ethics Committee of Hainan General Hospital (Ethical approval No. Med-Eth-Re [2025] 562). All research was conducted in accordance with relevant regulations and the Declaration of Helsinki.

Tissue (100 mg) was ground into powder with a homogenizer. RNA from the tissue was extracted via a TRIzol kit (Invitrogen™, Japan). The RNA was then reverse-transcribed into cDNA via a PrimeScript RT kit (RR047A) (Takara, Japan) at a concentration of 1000 ng. For qRT‒PCR, TB Green Premix Ex *Taq*II (RR820A) (Takara, Japan) was used. All samples were analyzed three times, and the data were processed via the 2^−ΔΔCt^ equation with GAPDH used as an internal reference gene to obtain the expression level of the target mRNA. The following primers were used: for SMURF2, 5′-ATGTCTAACCCCGGAGGC-3' (forward), 5′-GCATTGCCCAGATCCATCAAC-3' (reverse); for CCDC80, 5′-GCTTTTACGCCCGGAAACAA-3' (Forward), 5′-GTCCAGTCCTGGCGATTTCT-3' (Reverse); for GAPDH, 5′-AATGGGCAGCCGTTAGGAAA-3' (Forward), 5′-GCGCCCAATACGACCAAATC-3' (Reverse).

Total protein extraction from tissues: A 100 mg tissue sample was collected, cut on ice, and washed twice with precooled PBS, after which 1 mL of protein lysis buffer (RIPA) was added. After being fully lysed via an electric homogenizer and centrifuged at 12,000×*g* for 5 min at 4 °C, the supernatant was transferred to a precooled Eppendorf tube to extract the cellular protein. The total amount of protein was determined via a BCA protein quantification kit (KGB2101), and finally, the sample preparation was completed by adding 5 × loading buffer in a boiling water bath for 10 min. The prepared samples were separated via sodium dodecyl sulfate‒polyacrylamide gel electrophoresis (SDS‒PAGE) and transferred to a PVDF membrane. After being blocked in 5 % skim milk for 1 h at room temperature on a shaker, the membrane was incubated with the following antibodies: anti-SMURF2 (T58020) (Abmart, 1:1000) and anti-CCDC80 (PK90179) (Abmart, 1:500). Anti-GAPDH (GB11002-100) (Servicebio, 1:5000) was then used, and the samples were blocked at 4 °C overnight. The membrane was washed 3 times with TBST for 10 min each and then incubated with an HRP-labeled goat anti-rabbit IgG secondary antibody (GB23303) (Servicebio) for 1 h at room temperature. The membrane was washed three times with TBST and developed with enhanced chemiluminescence (ECL) solution (32209) (Thermo Fisher Scientific, USA).

### 2.11 Statistics

The bioinformatics analysis data calculations were performed via R (version 4.2.1) software. The experimental validation data were analyzed and graphed via GraphPad Prism 9 (version 9.4.0), and the images were organized and combined via Adobe Illustrator (version 26.3.1). All the data are expressed as the means ± SDs, and significant differences among groups were determined via a *t*-test. A P value less than 0.05 was considered significant.

## 3 Results

### 3.1 DEGs were screened by differential expression analysis and WGCNA

**Fig. 1:**
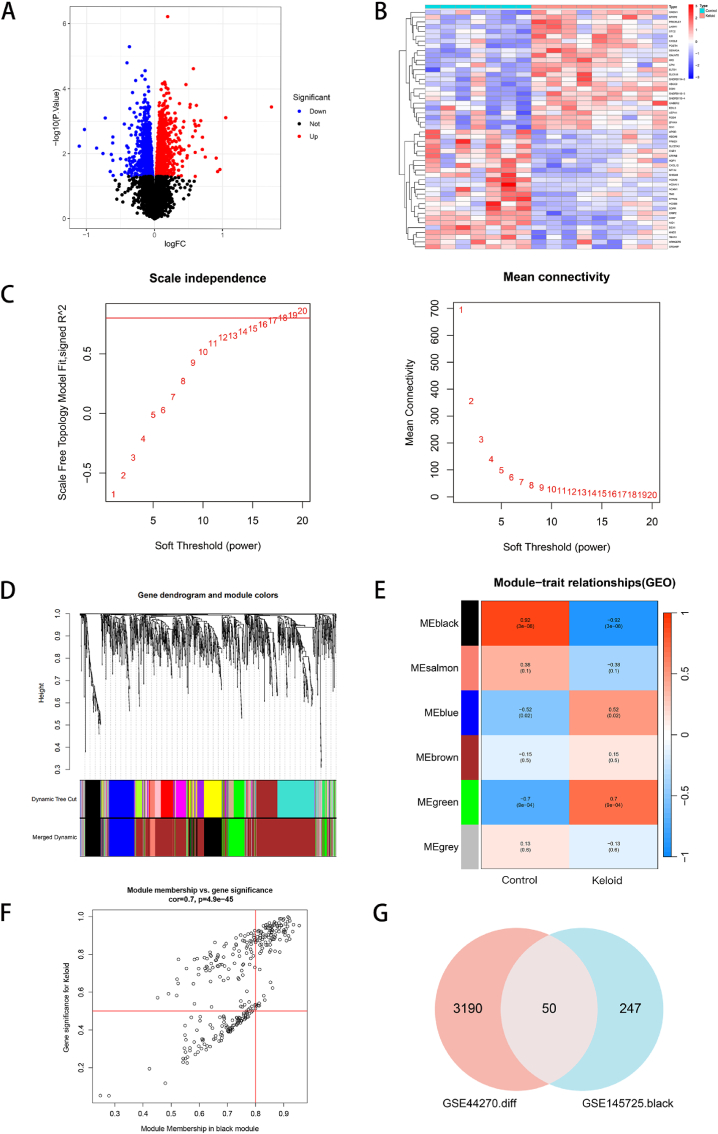
Screening of DEGs and construction of the WGCNA network. (A) Volcano plot showing the DEGs in the GSE44270 dataset. Red represents upregulated genes, blue represents downregulated genes, and black represents genes whose expression did not significantly differ. (B) Heatmap showing the top 50 DEGs. Red represents high expression, and blue represents low expression. (C) Network topology and average connectivity network of different soft thresholds in the GSE145725 WGCNA dataset. The red line represents the optimal soft threshold (β = 18, R^2^ = 0.9). (D) The gene clustering tree plot. “Dynamic Tree Cut” represents the initial modules, and “Merged dynamic” represents the final modules; each branch (or vertical line) represents a gene, and genes not assigned to any module are shown in gray. (E) The module‒trait association plot. Each row represents a module, and each column represents a trait attribute. Red indicates a positive correlation, and blue indicates a negative correlation. (F) Scatter plot of MM and GS in the KD group. There is a strong correlation between MM and GS, and the dots in the plot represent all the genes in the module. (G) Venn diagram of the intersection between DEGs and key modules.

First, differential analysis of the GSE44270 dataset revealed 3240 DEGs, including 1647 upregulated genes and 1593 downregulated genes (Fig. 1A). The heatmap shows the top 50 DEGs (Fig. 1B). Key gene modules significantly associated with KDs were identified via WGCNA. We chose 18 as the optimal soft threshold (R^2^ = 0.9) to establish a scale-free network (Fig. 1C). The cluster tree showed the original and merged modules (Fig. 1D). After merging, six highly correlated modules with characteristic genes were obtained. The module‒trait relationships were subsequently evaluated and visualized. The black module was positively correlated with normal (r = 0.92, p = 3e−08) and negatively correlated with KDs (r = −0.92, p = 3e−0.8) (Fig. 1E). In addition, the correlation between module membership (MM) and gene importance (GS) of the black module (r = 0.7, p = 4.9e-45) was significant, which also reflected the importance of the module (Fig. 1F). Finally, after the intersection of the 297 genes in the black module and 3240 DEGs, a total of 50 key genes were obtained for subsequent analysis (Fig. 1G).

### 3.2 Enrichment analysis to explore the biological functions of key genes

**Fig. 2:**
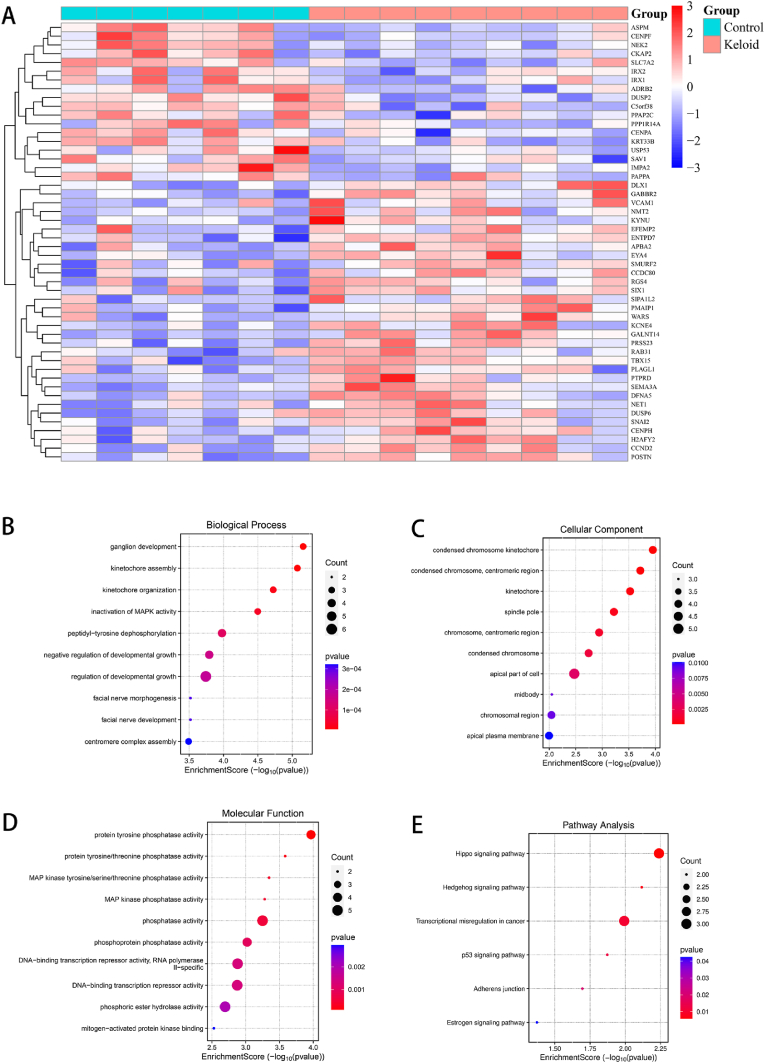
Display and enrichment analysis of the intersecting genes. (A) Heatmap of intersection genes, where red represents high expression and blue represents low expression. (B, C, D) Bubble plots of the GO analysis results, with the size of the dots representing the number of genes and the color representing the p value. (E) Bubble plot of the KEGG analysis results, where the size of the dots represents the number of genes and the color represents the p value.

First, we showed the expression of 50 key genes via a heatmap (Fig. 2A). To understand the functions and pathways associated with these genes, we conducted functional enrichment analysis on 50 key genes. GO-BP analysis revealed that the key genes were significantly enriched in ganglion development and kidneytochore assembly (Fig. 2B). GO-CC analysis revealed that key genes were significantly enriched in condensed chromosome kinetochores, condensed chromosomes, and centromeric regions (Fig. 2C). GO-MF analysis revealed that key genes were significantly enriched in protein tyrosine phosphatase activity and phosphatase activity (Fig. 2D). In addition, key genes were involved in signaling pathways such as the Hippo signaling pathway and transcriptional misregulation in cancer (Fig. 2E).

### 3.3 Machine learning identification of candidate genes and intergene correlation analysis

**Fig. 3:**
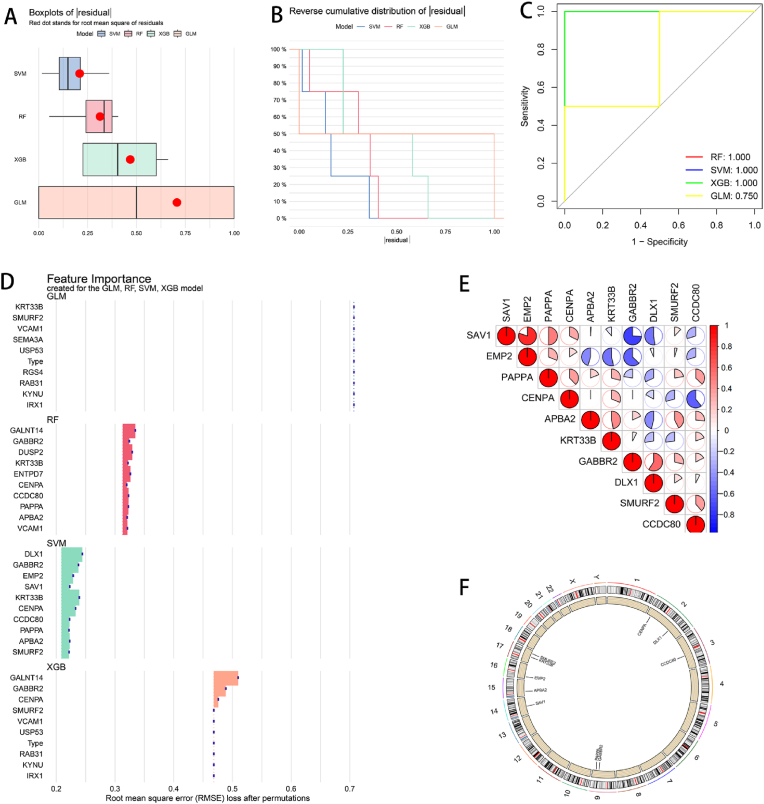
Candidate genes screened by the machine learning algorithm. (A) Residual boxplots of the four algorithms. (B) Inverse cumulative distribution plot of the residuals. (C) ROC curve. (D) Feature importance map. (E) Correlation heatmap of 10 candidate genes. The size of the circles indicates the strength of the correlation; red represents a positive correlation, and blue represents a negative correlation, with darker colors indicating stronger correlations. (F) Locations of the 10 candidate genes on the chromosomes.

Machine learning models were constructed via the SVM, RF, GLM, and XGB algorithms. The residual distributions of the four algorithms were calculated and compared (Fig. 3A and B). The SVM algorithm had the smallest root mean square of the residuals (Fig. 3A), indicating that it had the highest prediction accuracy in predicting the KD sample. The SVM algorithm also performed best in terms of the inverse cumulative distribution of the absolute values of the residuals (Fig. 3B). ROC curves were used to further evaluate the prediction reliability of the four models (Fig. 3C). The areas under the curve (AUCs) of the SVM, RF, and XGB models all had good performance at 1.0; the AUC of the GLM was 0.75, indicating poor performance. The top 10 genes with the highest importance scores in each model were selected to draw the feature importance map. The variation in the root mean square error (RMSE) of the SVM model was relatively small, indicating that the SVM model had better stability (Fig. 3D). On the basis of the above results, we considered that the SVM had good predictive ability, so 10 genes in the SVM algorithm were selected as candidate genes for further analysis.

We calculated the Pearson correlation coefficient of the candidate genes to generate a correlation heatmap of the 10 candidate genes. The color represents the strength of the correlation; red represents a positive correlation, and blue represents a negative correlation. The darker the color is, the stronger the correlation. Fig. 3E shows that there are interactions among the 10 candidate genes. The chromosome location map revealed that the 10 candidate genes were scattered on different chromosomes (Fig. 3F).

### 3.4 Validation of candidate genes and construction and evaluation of diagnostic models

**Fig. 4:**
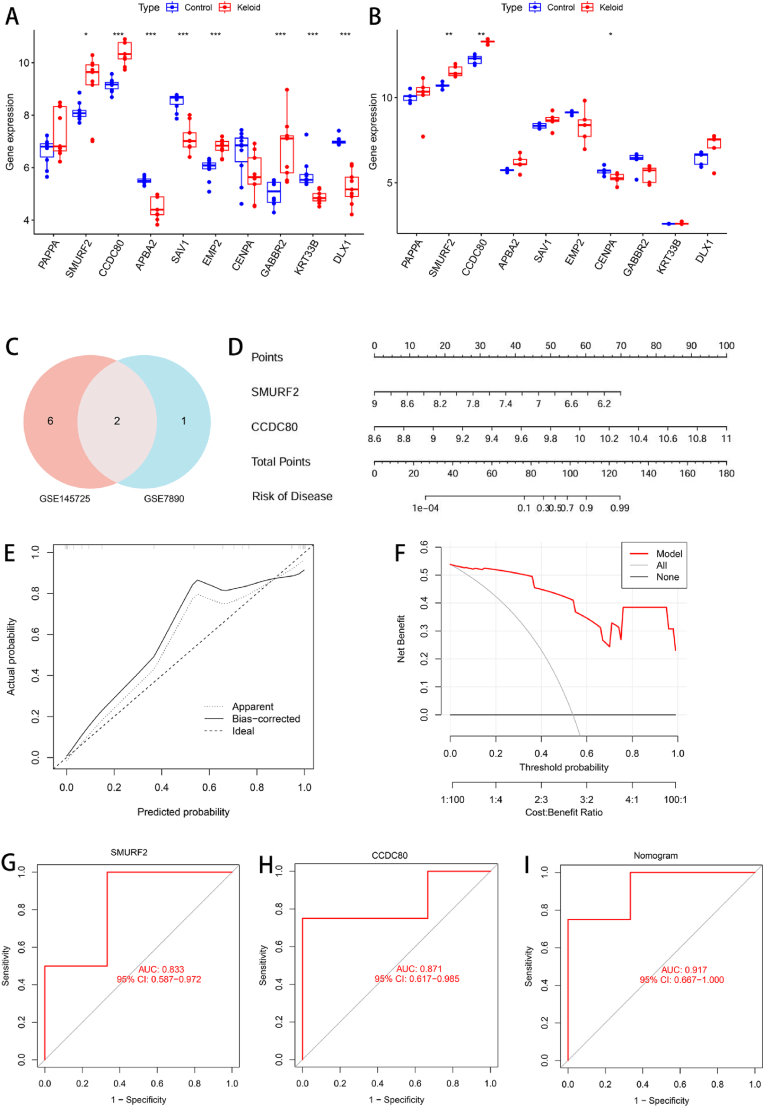
Construction and evaluation of the external validation and diagnostic models. (A) Boxplots showing the expression of candidate genes in the external dataset GSE145725. (B) Boxplots showing the expression of candidate genes in the external dataset GSE7890. (C) Venn diagram of the intersection of significantly expressed genes. (D) Nomogram model for predicting KD risk on the basis of the expression levels of SMURF2 and CCDC80. (E) Calibration curve of the nomogram model predictions. (F) Decision curve analysis (DCA) of the nomogram model predictions. (G) ROC curve for the diagnostic performance of SMURF2. (H) ROC curve for the diagnostic performance of CCDC80. (I) ROC curve for the diagnostic performance of the model. (∗P < 0.05, ∗∗P < 0.01, ∗∗∗P < 0.001).

The expression validation of the 10 candidate genes screened by machine learning was performed via an external dataset. Eight genes in GSE145725 were significantly differentially expressed: SMURF2, CCDC80, APBA2, SAV1, EMP2, GABBR2, KRT33B, and DLX1 (Fig. 4A). Three genes were significantly differentially expressed in GSE7890: SMURF2, CCDC80, and CENPA (Fig. 4B). After the genes intersected, two diagnostic marker genes, SMURF2 and CCDC80, were obtained (Fig. 4C). We performed multivariate logistic regression modeling on these 2 genes and developed a nomogram for KD diagnosis (Fig. 4D). In addition, the calibration curve revealed that the difference between the actual and predicted disease risks was small, indicating that the KD prediction accuracy was high (Fig. 4E). DCA revealed that the curve was located above the gray line, indicating that the model had potential value (Fig. 4F). We then performed external validation and plotted ROC curves to assess their specificity and sensitivity in diagnosis. The area under the curve (AUC) was calculated to obtain SMURF2 (AUC = 0.833) and CCDC80 (AUC = 0.871) (Fig. 4G and H). Moreover, the ROC curve was used to evaluate the model, and the calculated AUC was 0.917 (Fig. 4I). In summary, the results showed that the diagnostic model composed of SMURF2 and CCDC80 had high diagnostic value.

### 3.5 Correlation analysis of immune infiltration, diagnostic markers and immune cells

**Fig. 5:**
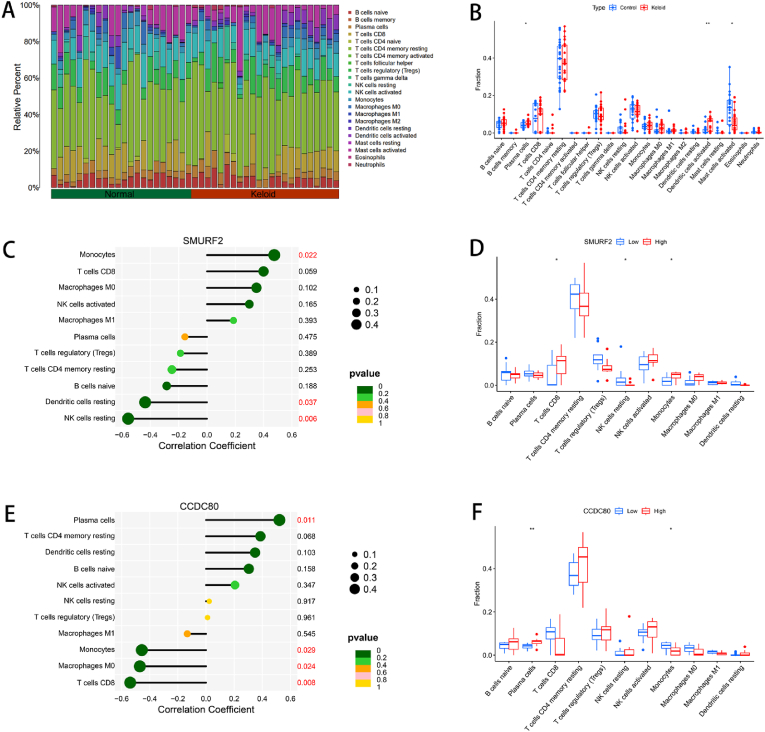
Immune infiltration analysis and correlation analysis of diagnostic markers and immune cells. (A) Stacked histogram of the proportions of 22 types of immune cells. (B) Box plot of the infiltration of 22 types of immune cells in KD and normal samples. (C) Lollipop chart showing the correlation between SMURF2 and immune cells. The size of the dots represents the strength of the correlation between the gene and immune cells: the larger the dot is, the stronger the correlation; the smaller the dot is, the weaker the correlation. The color of the dots represents the p value. (D) Box plot of the effect of the SMURF2 gene expression level on immune cells. (E) Lollipop chart of the correlation between CCDC80 and immune cells. (F) Box plot of the effect of the CCDC80 gene expression level on immune cells. (∗P < 0.05, ∗∗P < 0.01).

Next, immune cell infiltration in different samples was analyzed. The proportions of the 22 types of immune cells are presented in bar graph form. In the two groups, memory B cells and activated memory T cells accounted for the smallest proportion, whereas resting memory T cells, follicular helper T cells, CD8^+^ T cells, and M2 macrophages accounted for a large proportion (Fig. 5A). The proportions of plasma cells (P < 0.05) and activated dendritic cells (P < 0.01) were greater in the KD group than in the control group; however, the proportion of activated mast cells was lower (P < 0.05). (Fig. 5B). To further study the effects of SMURF2 and CCDC80 on 22 types of immune cells, we used Lollipop charts to determine the correlations between immune cells and diagnostic marker genes. SMURF2 was positively correlated with monocytes and negatively correlated with resting dendritic cells and resting NK cells (Fig. 5C). CCDC80 was positively correlated with the number of plasma cells and negatively correlated with the number of monocytes, M0 macrophages, and CD8^+^ T cells (Fig. 5E). In addition, the expression level of SMURF2 affected the immune infiltration level of CD8^+^ T cells, resting NK cells, and monocytes (P < 0.05) (Fig. 5D), and the expression level of CCDC80 affected the immune infiltration level of plasma cells and monocytes (P < 0.05) (Fig. 5F).

### 3.6 Consistency clustering and immune infiltration of subtypes and subtype differences and enrichment analysis

**Fig. 6:**
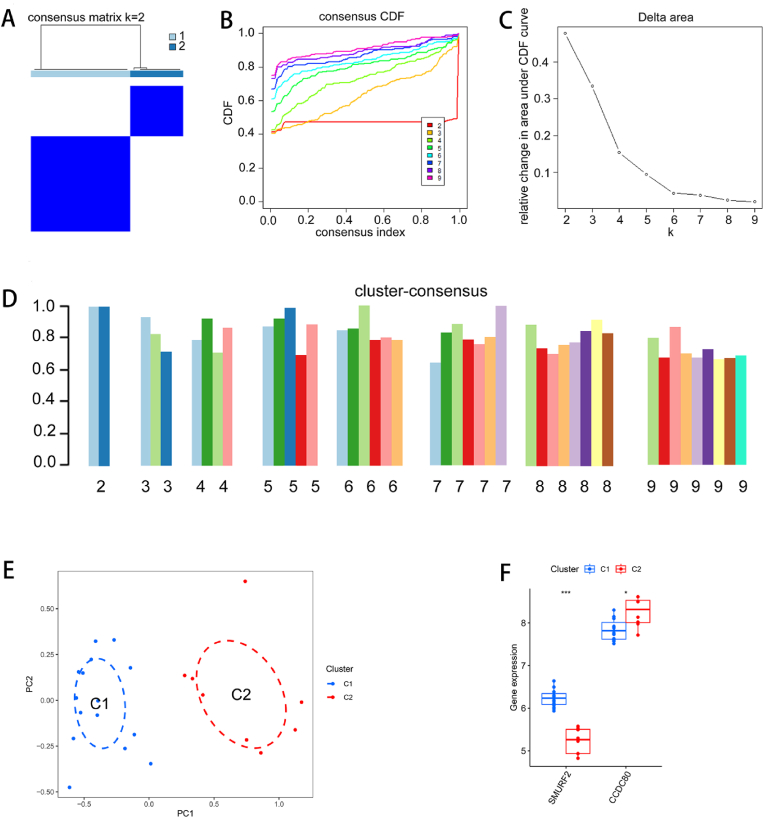
Consistency cluster analysis. (A) Consensus set clustering matrix when k = 2. (B) Representative CDF curves when k = 2–9. (C) Relative change in the area under the CDF curve. (D) Cluster score for each subtype when k = 2–9. (E) PCA revealed that KD patients can be divided into two different subtypes. (F) Box plot of the expression of diagnostic markers in different classifications. (∗P < 0.05, ∗∗∗P < 0.001).

The KD dataset was clustered via consensus cluster analysis. On the basis of the consensus matrix, CDF plot, relative changes in the area under the CDF curve, and consistency clustering score (>0.9), the optimal number of subtypes was comprehensively determined to be two (Fig. 6A–D), and the two isoforms were named C1 and C2. The PCA plot revealed significant differences among the subtypes (Fig. 6E).

**Fig. 7:**
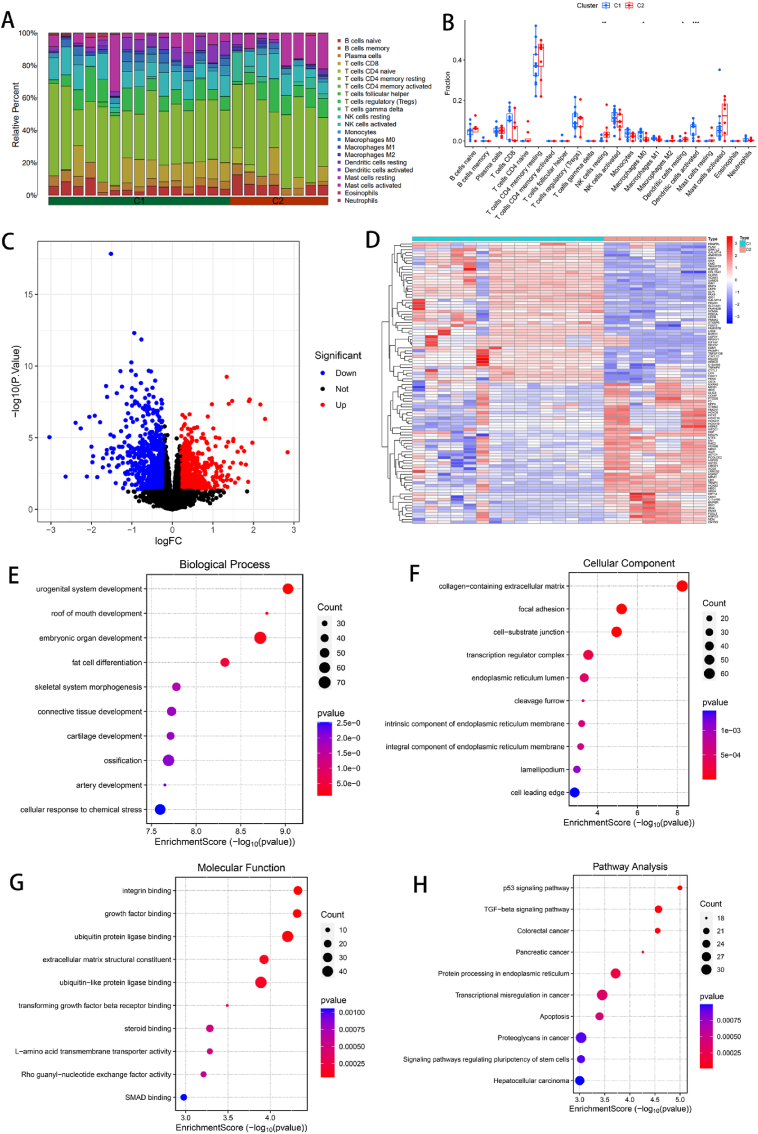
Types of immune infiltration and differences between types and functional enrichment analysis. (A) Stacked histogram of the proportions of 22 types of immune cells in different classifications. (B) Box plot of differences in immune cell infiltration among different classifications. (C) Volcano plot for differential expression analysis between different subtypes. Red represents upregulated genes, blue represents downregulated genes, and black represents genes whose expression did not significantly differ. (D) Heatmap of the top 100 DEGs; red indicates high expression, and blue indicates low expression. (E, F, G) Bubble plots of the GO analysis results for the DEGs, where the size of the dots represents the number of genes and the color represents the p value. (H) Bubble plot of the KEGG analysis for DEGs, where the size of the dots represents the number of genes and the color represents the p value. (∗P < 0.05, ∗∗P < 0.01, ∗∗∗P < 0.001).

To elucidate the molecular differences between these subtypes, we first evaluated the differential expression of diagnostic markers between the different subtypes. SMURF2 was more highly expressed in the C1 subtype, whereas CCDC80 was more highly expressed in the C2 subtype. The expression of these genes is shown in Fig. 6F. Immune infiltration analysis revealed significant differences between the C1 and C2 groups in terms of the infiltration status of resting dendritic cells, M0 macrophages, resting NK cells, and activated dendritic cells (Fig. 7A and B). A total of 1582 DEGs were found between the two groups, including 689 upregulated genes and 893 downregulated genes (Fig. 7C). The heatmap shows the top 100 DEGs. All DEGs could be well separated by group, with significant heterogeneity between subtypes (Fig. 7D). GO analysis revealed that the 370 DEGs were enriched mainly in the terms “urogenital system development”, “embryonic organ development”, “collagen-containing extracellular matrix”, “focal adhesion”, “integrin binding”, and “extracellular matrix structural constituent” (Fig. 7E, F, and G). In the KEGG analysis, these DEGs were significantly enriched in pathways such as the p53 signaling pathway and the TGF-beta signaling pathway (Fig. 7H).

### 3.7 ceRNA network construction

**Fig. 8:**
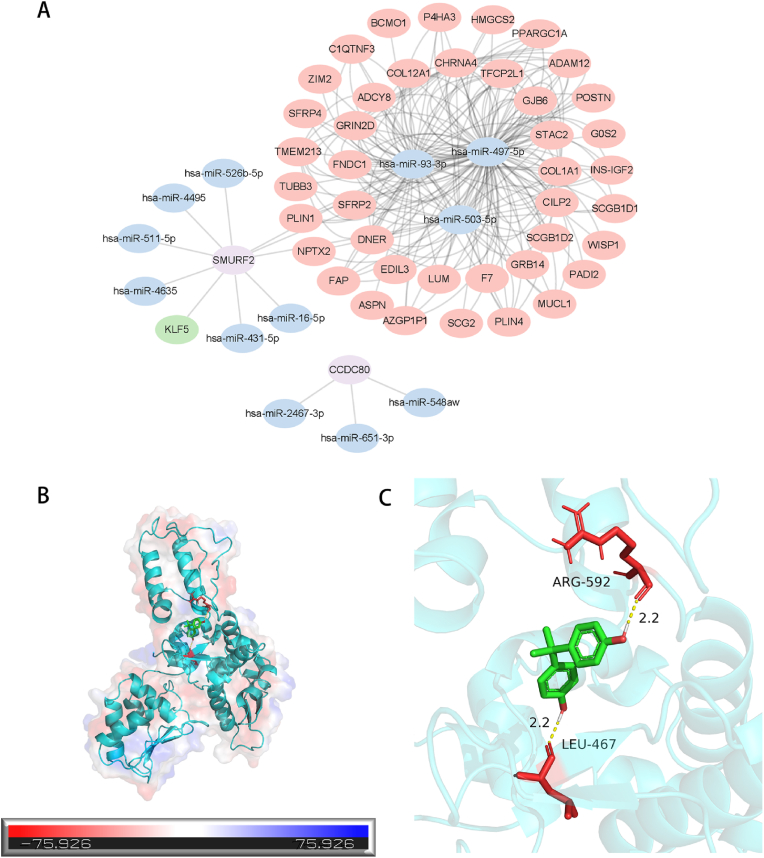
ceRNA regulatory network of SMURF2 and CCDC80 and their drug screening and molecular docking analyses. (A) lncRNA‒miRNA‒mRNA‒TF regulatory network. (B) Overall situation of the molecular docking of SMURF2 and bisphenol A. (C) Local amplification details of the molecular docking between SMURF2 and bisphenol A (the yellow dashed lines represent the two pairs of hydrogen bonds formed).

After the intersection of the lncRNAs identified via GSE83286 and the lncRNAs predicted via the miRcode database, a regulatory network consisting of lncRNAs, miRNAs, mRNAs, and TFs was constructed after 41 lncRNAs were obtained. In the ceRNA regulatory network, we identified 1 TF, 9 miRNAs and 41 lncRNAs that interact with SMURF2 mRNA. Three miRNAs interact with CCDC80 mRNA (biomarkers in purple, miRNAs in blue, TFs in green, and long noncoding RNAs (lncRNAs) in pink) (Fig. 8A).

### 3.8 Prediction and molecular docking of potential drugs related to treatment

Forty-eight compounds or small-molecule drugs related to SMURF2 were predicted via the CTD database, 96 compounds related to CCDC80 were predicted, and 24 drugs were identified after the intersection of the two. To evaluate the binding energy and interaction modes between the drug candidate and its target, we performed molecular docking analysis. Molecular docking studies of SMURF2 and bisphenol A revealed that this molecule can bind through residues with a static potential energy of approximately 75.926 (Fig. 8B). Local amplification details of the molecular docking of SMURF2 and bisphenol A revealed that bisphenol A binds to the proteins ARG-592 and LEU-467. Two pairs of hydrogen bonds were formed (yellow dotted line) (Fig. 8C).

### 3.9 qRT‒PCR and WB of clinical samples

**Fig. 9:**
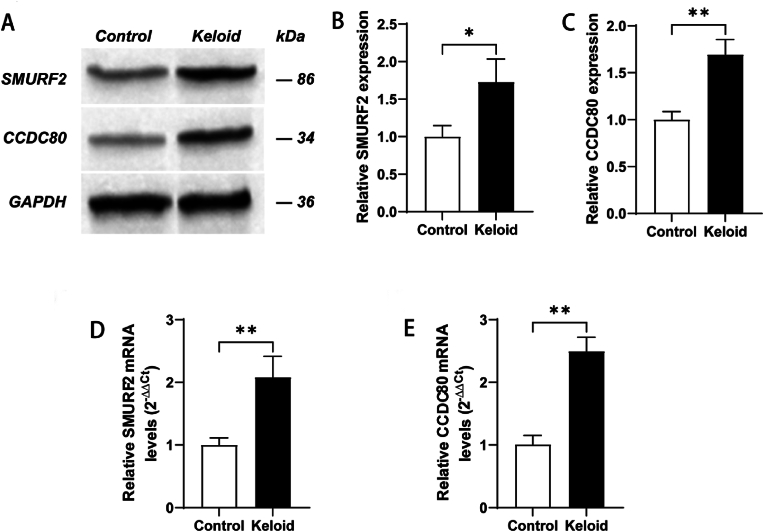
Validation of the expression of SMURF2 and CCDC80 in clinical samples. The expression of SMURF2 (A, B) and CCDC80 (A, C) in human skin tissues is shown via western blotting. The levels of SMURF2 (D) and CCDC80 (E) in the indicated groups of human skin tissues were detected via qPCR. (∗P < 0.05, ∗∗P < 0.01).

To further validate the diagnostic value of SMURF2 and CCDC80, we performed qRT‒PCR and WB on the collected clinical samples to further validate their mRNA and protein expression. Western blot analysis revealed that protein expression in KD patients was greater than that in the control group (Fig. 9A, B, and C). In addition, the mRNA levels of SMURF2 and CCDC80 were increased in KD patients compared with controls (Fig. 9D and E).

## 4 Discussion

KD is a pathological phenomenon in the tissue healing process that is often accompanied by excessive fibrosis and collagen deposition, and its mechanism of action remains unclear [31]. Traditional treatment methods have limited effects and high recurrence rates, so early diagnosis and timely and effective intervention are particularly critical [32]. Identifying genes associated with KD is helpful for identifying new diagnostic markers and drug targets. In this study, 50 DEGs were screened via difference analysis and WGCNA, and 10 candidate genes (SMURF2, CCDC80, APBA2, SAV1, EMP2, GABBR2, KRT33B, and DLX1) were identified. Two external datasets, GSE145725 and GSE7890, were used to validate the results, and two diagnostic marker genes, SMURF2 and CCDC80, whose expression was upregulated, were ultimately obtained. We subsequently used logistic regression to construct a diagnostic model using these 2 genes. ROC curves revealed that SMURF2 (AUC = 0.833) and CCDC80 (AUC = 0.871) had good predictive value in the diagnosis of KD, validating their potential as diagnostic markers. Similarly, the ROC curves of the model also proved that the model had high diagnostic value. These findings provide a new molecular target for the early diagnosis of KD.

Smad ubiquitination regulatory factor 2 (SMURF2) is an E3 ubiquitin ligase that is homologous to the E6-AP carboxyl terminus family and regulates processes such as cell proliferation, migration and apoptosis [33]. The regulation of different signaling pathways affects the occurrence and development of various diseases [34]. In fibrosis-related diseases, SMURF2 inhibits the expression of junctional tissue growth factors and exerts its antifibrotic effect by regulating the cAMP–PKA–CREB signaling pathway [35]. SMURF2 is a ubiquitin ligase of the Smad protein. The Smad protein is an important intracellular mediator of TGF-β1 signaling. Compared with those in normal fibroblasts, SMURF2 protein and mRNA levels in hypertrophic scar fibroblasts are increased, and SMURF2 expression [36] is increased after TGF-β1 stimulation. SMURF2 inhibits the TGF-β signaling pathway and affects ECM synthesis and degradation by promoting Smad7 ubiquitination [37] and can also affect inflammation and collagen synthesis by regulating the TGF-β/Smad3 signaling pathway [38]. In the study of KD, continuous activation of the TGF-β/Smad signaling pathway was the main cause of the excessive proliferation of fibroblasts and myofibroblasts, and therapeutic strategies targeting this pathway may relieve the development of KD [39]. SMURF2, as a regulator, is expected to become a new target for KD treatment.

Coiled-coil domain containing 80 (CCDC80) belongs to the CCDC family. This family contains approximately 180 genes. The encoded proteins are involved in a variety of biological processes, such as intercellular signal transduction and gene transcription, as well as the regulation of the growth, invasion, and metastasis of various cancers. CCDC58 plays an important role in and is also involved in the regulation of the TGF-β, MAPK, and PI3K/Akt-related signaling pathways [40]. For example, CCDC58 was found to be a potential pancancer biomarker, and its expression closely related to the diagnosis, prognosis and level of immune infiltration of various tumors [41]. Studies have shown that CCDC80 expression is downregulated in ovarian cancer and may function as a tumor suppressor [42]. CCDC80 is associated with immune cell infiltration in highly expressed gastric cancer subtypes. CCDC80 silencing can inhibit M2 polarization and the JAK-STAT pathway in gastric cancer cells and tumor tissues [43]. CCDC family genes are associated with various mechanisms and signaling pathways involved in the development of KD. The potential pathophysiological process of this family in KD remains to be further explored.

KDs are closely related to the immune microenvironment, and genes associated with immune infiltration and immune cell abnormalities may be the basis of KD [44]. Therefore, we combined the GSE44270, GSE145725, and GSE7890 datasets to study the extent of immune cell infiltration in GEO database samples. Significant differences in plasma cells, activated dendritic cells, and activated mast cells were detected between KD and normal tissues. Plasma cells may be associated with the development and progression of KD by participating in the immune response, producing cytokines, regulating the inflammatory environment and affecting signaling pathways [45]. Dendritic cells bind to corresponding receptors on the surface of T lymphocytes through the binding of costimulatory molecules on the surface to activate T lymphocytes [46]. Activated T lymphocytes then secrete a variety of cytokines, such as IFN-γ and IL-4, thereby regulating the activity of fibroblasts and collagen synthesis [47,48]. In addition, activated dendritic cells can also secrete some cytokines, which induce the expression of fibroblasts, adhesion molecules and extracellular matrix proteins, affecting the structure and components of scar tissue [49]. Mast cell activation is a key factor that causes chronic inflammation in scars. Mast cell activation causes a persistent chronic inflammatory state in scars by releasing inflammatory mediators such as histamine, IL-6, and IL-8 and induces excessive ECM synthesis and vascularization in proliferative wounds, leading to scar formation [50]. Additionally, this study elucidated the correlation between potential biomarkers and immune cells. We found that SMURF2 was positively correlated with monocytes and negatively correlated with resting dendritic cells and resting NK cells; the expression level of SMURF2 had a significant effect on the infiltration of CD8^+^ T cells, resting NK cells, and monocytes into the water. CCDC80 was positively correlated with the number of plasma cells and negatively correlated with the number of monocytes, M0 macrophages, and CD8^+^ T cells. In addition, the expression level of CCDC80 had a significant effect on the level of immune infiltration of plasma cells and monocytes. Monocytes are the precursors of macrophages, and M0 macrophages are undifferentiated macrophages that differentiate into mature macrophages after they enter tissues [51]. These two proteins play complementary roles in the immune response and tissue homeostasis [52]. In KD, macrophages secrete proinflammatory factors such as IL-1β, TNF-α, and IL-6 to promote inflammation, stimulate collagen production and angiogenesis, and initiate the healing process [53]. As the wound environment changes, M1-type macrophages may convert to M2-type macrophages, further promoting fibrosis [54]. Furthermore, studies have shown that IFN-γ produced by NK cells plays a key role in limiting the progression of KD by inducing fibroblast apoptosis and inhibiting the overproduction of the extracellular matrix, whereas NK cell-derived AREG (amphiregulin) promotes cell proliferation and survival and protects against IFN-γ [55]. The two diagnostic markers we identified were both associated with immune cell infiltration. The regulation of the disordered immune status on the basis of these two genes is expected to become a potential strategy for the treatment of KD. However, future studies need to further explore the function of biomarkers such as SMURF2 and CCDC80 in immune cell infiltration and how they affect the immune microenvironment in KD patients to provide important information for the development of new treatment strategies in the future.

Furthermore, via consensus cluster analysis, we generated two subtypes on the basis of the gene expression profiles of the two diagnostic markers. The C1 subtype exhibited increased expression of SMURF2, whereas the C2 subtype was characterized by increased expression of CCDC80. The functional enrichment analysis revealed that the DEGs were enriched mainly in biological functions such as urogenital system development, embryonic organ development, the collagen-containing extracellular matrix, focal adhesion, integrin binding, and the extracellular matrix structural constituent. KEGG analysis revealed that the DEGs were significantly enriched in the p53 signaling pathway and the TGF-beta signaling pathway. Immune infiltration analysis revealed significant differences between the C1 and C2 groups with respect to the infiltration status of resting dendritic cells, M0 macrophages, resting NK cells, and activated dendritic cells. We further confirmed that immune cell dysregulation is associated with the development of KD from different angles.

To further elucidate the regulatory mechanism of gene expression, we constructed a lncRNA‒miRNA‒mRNA‒TF regulatory network based on the SMURF2 and CCDC80 genes, which revealed the complex interactions of related genes in the regulatory pathway. In the network, miRNAs were used as the core nodes, connecting upstream lncRNAs and downstream mRNAs and TFs. Among them, SMURF2 was significantly associated with various miRNAs (such as hsa-miR-4635 and hsa-miR-16-5p) and had a potential regulatory relationship with KLF5, a key TF. The regulatory network of CCDC80 is relatively simple and is regulated mainly by hsa-miR-548aw, hsa-miR-2467-3p and hsa-miR-651-3p. The overall network revealed that hsa-miR-93-3p, hsa-miR-497-5p and hsa-miR-503-5p formed a highly connected regulatory module by targeting multiple downstream mRNAs (e.g., COL1A1 and FNDC1), reflecting their <br> important role in signaling pathways. In addition, as an important TF downstream of SMURF2, KLF5 may play a key role in the regulation of signaling pathways. The construction of this network provided important clues for exploring the regulatory mechanisms of SMURF2 and CCDC80 and their potential roles in diseases.

Next, we performed drug sensitivity analysis on the two diagnostic markers and screened 24 small-molecule drugs or compounds for molecular docking. Two pairs of hydrogen bonds were formed between bisphenol A and ARG-592 and LEU-467 of the SMURF2 protein. Bisphenol A (BPA) is a synthetic organic compound with the chemical formula C_15_H_16_O_2_. BPA is used mainly to manufacture polycarbonate plastics and epoxy resins. These materials are widely used in food packaging, electronic products, automobiles, and construction materials [56]. Previous studies have shown that BPA is associated with a variety of health problems, including premature birth, allergic diseases, kidney disease, metabolic syndrome, polycystic ovary syndrome, obesity, type 2 diabetes, and cardiovascular diseases [57]. BPA destroys the function of the human endocrine system when it is administered through the diet [58]. However, a study by Xue et al. [59] BPA has dual effects on wound healing, which may provide a new method for pathological wound healing treatment. In the future, the effect of BPA on wound healing needs to be further verified. While evaluating its safety from multiple aspects, corresponding experiments are needed to verify its effects on keloids as well as the specific molecular mechanisms and regulatory targets involved.

In addition, to further verify the expression of the two diagnostic genes, we performed qRT‒PCR and WB experiments on clinical samples. The results revealed that the mRNA and protein expression levels of SMURF2 and CCDC80 were significantly greater than those in the control group. These findings further confirmed our results and provided intuitive evidence for the use of these two markers as diagnostic markers of KD.

This study has several limitations. First, our analysis is based on a limited number of clinical samples in public databases, which may contain errors due to the small sample size. In addition, although we have experimentally validated the differences in the expression of the genes identified as biomarkers, many clinical samples are still needed to further explore their expression status, and larger datasets are needed for further study and validation of the markers and molecular mechanisms we identified. Finally, although we successfully performed molecular docking, the safety and specific effects of BPA on KD need to be further evaluated in the future.

## 5 Conclusion

Through bioinformatics analysis and experimental validation, this study proposed for the first time that SMURF2 and CCDC80 could be used as potential diagnostic markers of KD. This study not only provides a new molecular target for the early diagnosis and treatment of KD but also helps to elucidate the molecular mechanisms involved in the development of KD. In addition, we identified potential interactions between BPA and SMURF2 at the molecular level. These finding provides a new direction for subsequent in-depth exploration of the potential application value of BPA and are expected to lead to the development of new ideas and methods for the prevention and treatment of KD. In the future, we will continue to carry out relevant studies to provide more means for the clinical treatment of KD.

## CRediT authorship contribution statement

**Ze Wang:** Writing – original draft, Validation, Formal analysis, Data curation. **Bo Hu:** Software, Investigation. **Wenfei Li:** Software, Investigation. **Tengxiao Ma:** Writing – review & editing, Supervision. **Lei Li:** Writing – review & editing, Supervision.

## Informed consent statement

Informed consent was obtained from all subjects involved in the study.

## Ethical approval

The studies involving human tissue specimens were reviewed and approved by the Ethical Review Committee of Hainan Affiliated Hospital of Hainan Medical University and was performed in compliance with the Declaration of Helsinki. The patients/participants provided their written informed consent to participate in this study.

## Funding

This research was funded by 10.13039/501100004761Natural Science Foundation of Hainan Province, grant number 823RC561.

## Declaration of competing interest

The authors declare that they have no known competing financial interests or personal relationships that could have appeared to influence the work reported in this paper.

## Data Availability

Data will be made available on request.
