# Supplementary figures and images for: Screening and validation of diagnostic markers for keloids via bioinformatics analysis
Source: Biochem Biophys Rep. 2025 Aug 22;43:102219. doi: 10.1016/j.bbrep.2025.102219 (PMC12420520; doi:10.1016/j.bbrep.2025.102219)

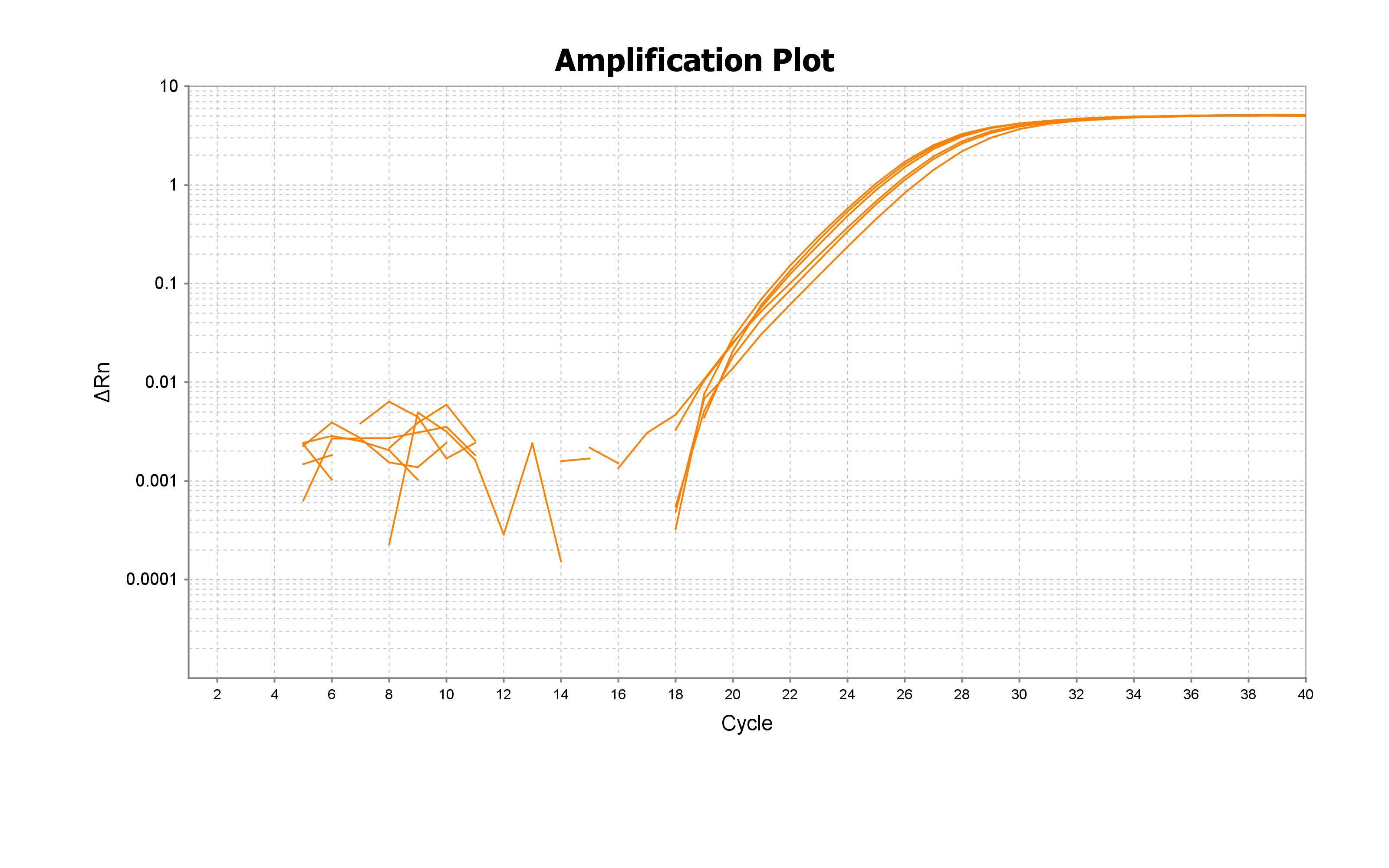

Supplement: Multimedia component 1 [file mmc1.zip › Western Blot and PCR raw data/Original/pcr/CCDC80/Amplification Plot-CCDC80.jpg]

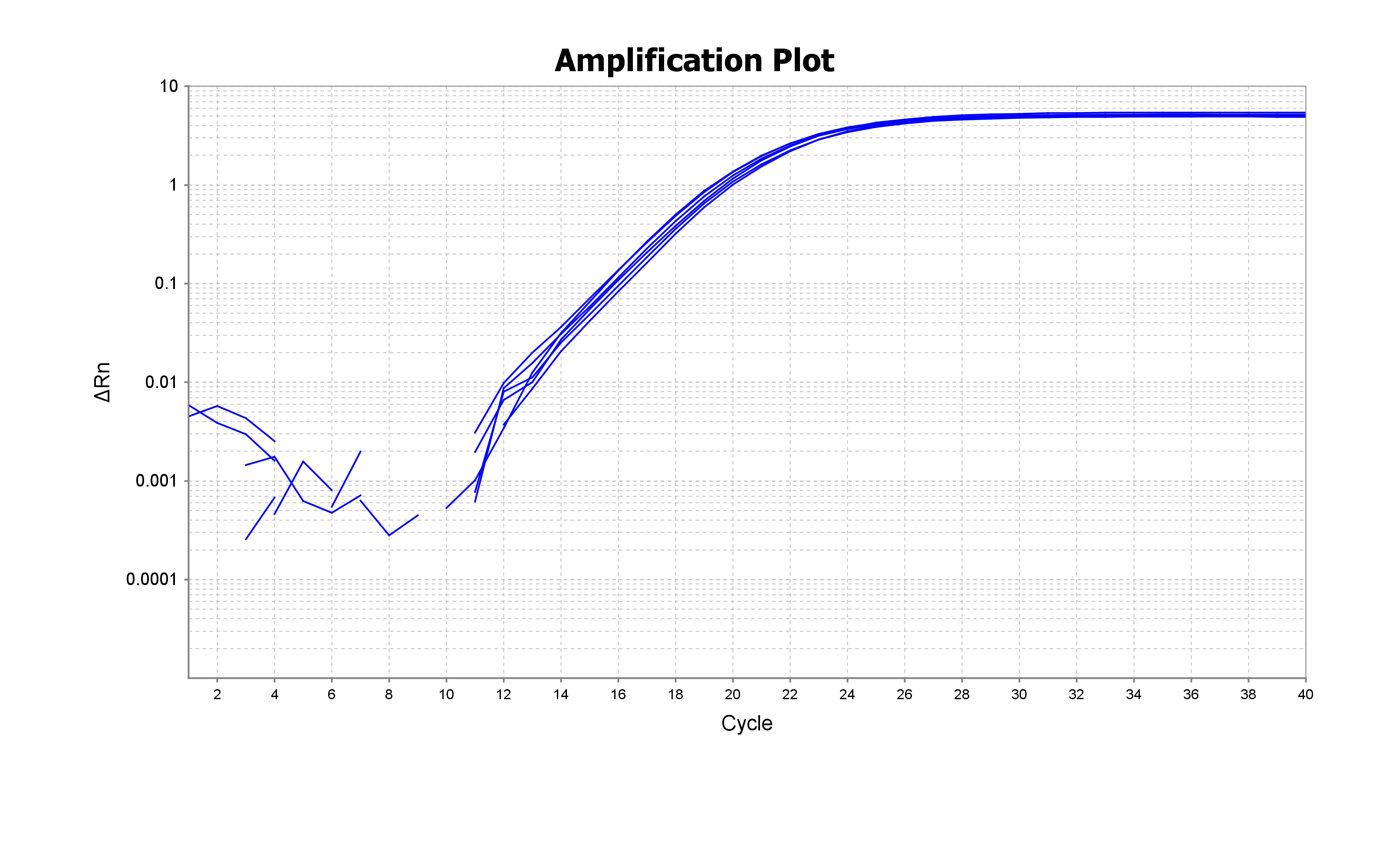

Supplement: Multimedia component 1 [file mmc1.zip › Western Blot and PCR raw data/Original/pcr/CCDC80/Amplification Plot-GAPDH.jpg]

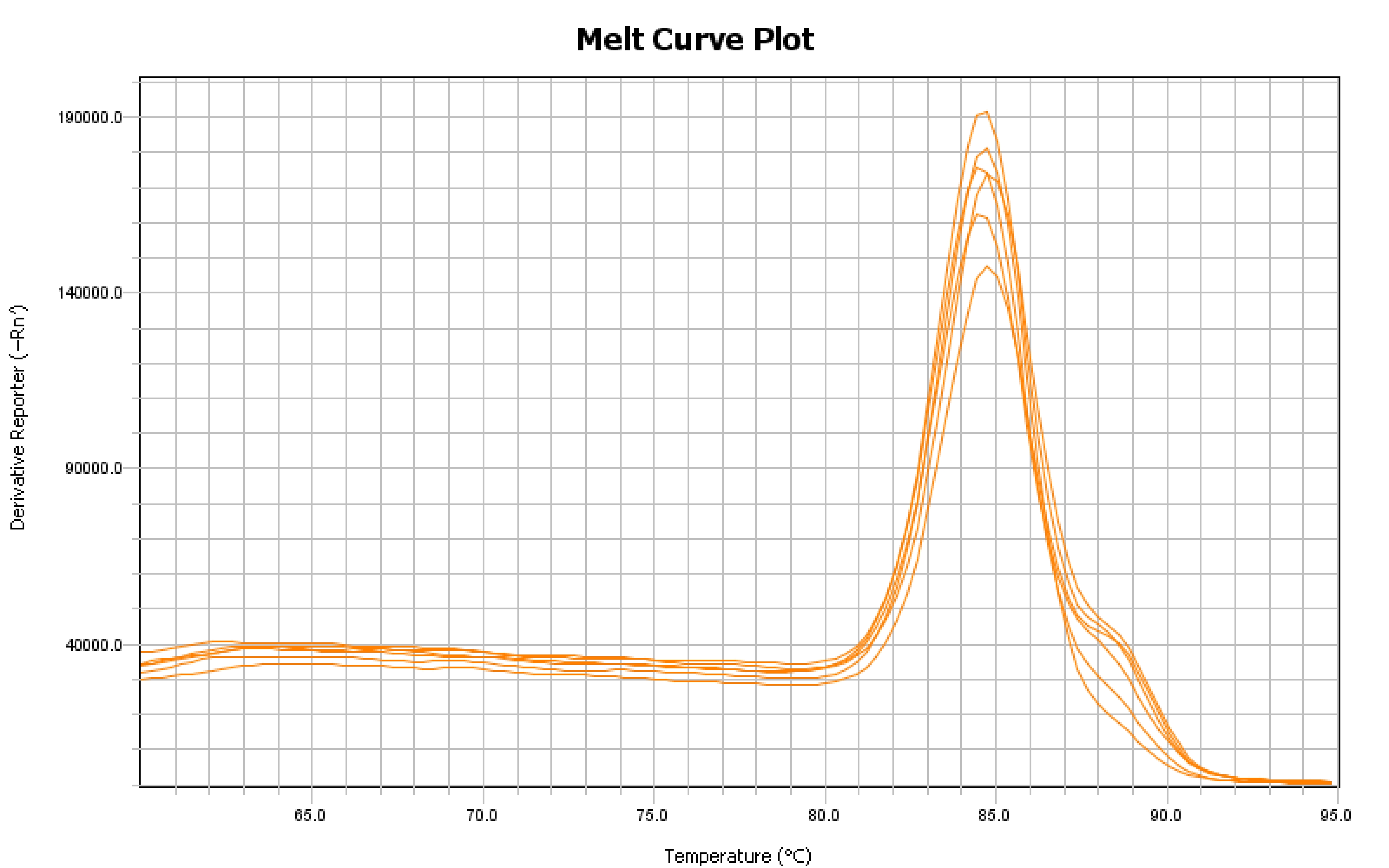

Supplement: Multimedia component 1 [file mmc1.zip › Western Blot and PCR raw data/Original/pcr/CCDC80/Melt Curve Plot-CCDC80.jpg]

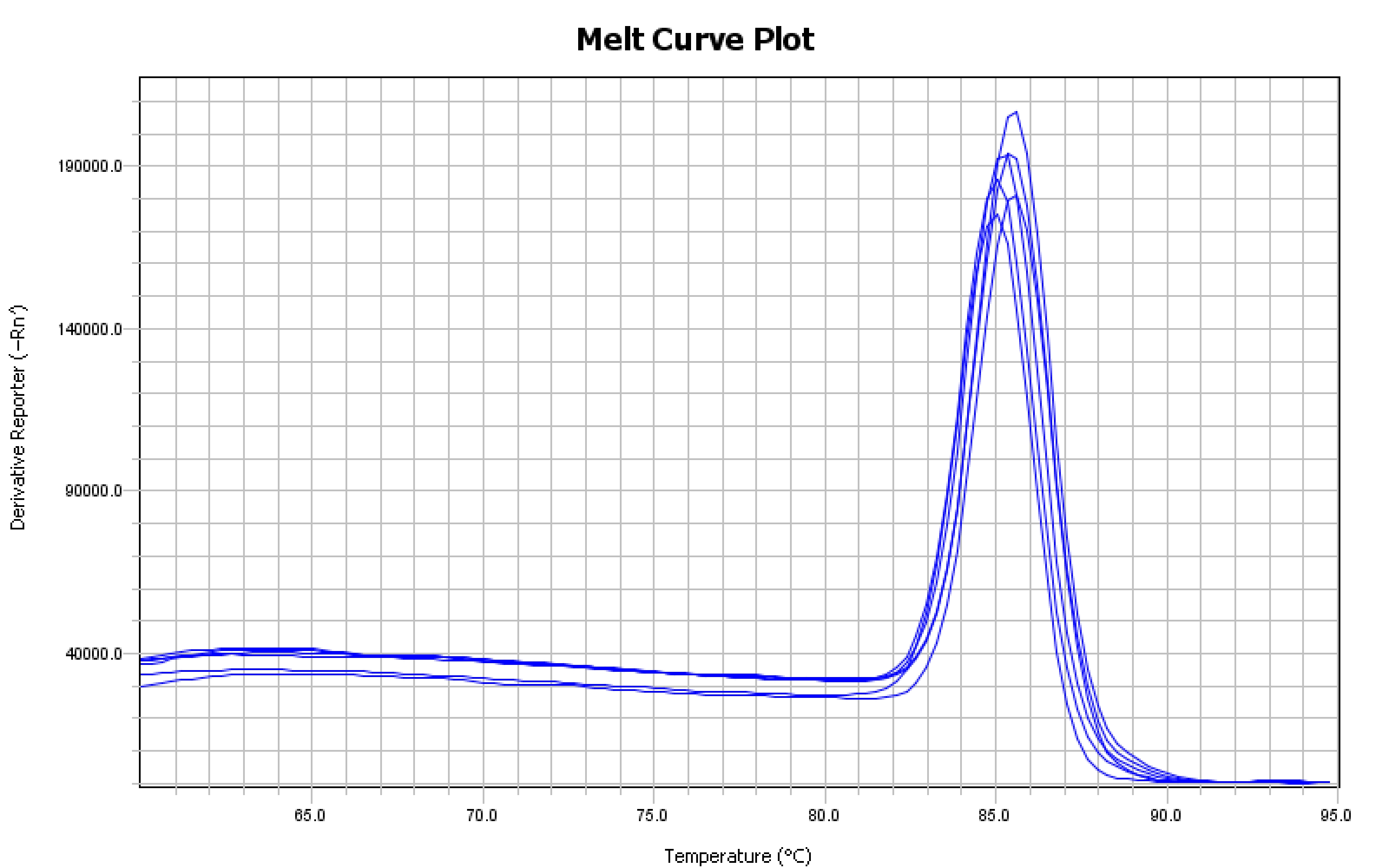

Supplement: Multimedia component 1 [file mmc1.zip › Western Blot and PCR raw data/Original/pcr/CCDC80/Melt Curve Plot-GAPDH.jpg]

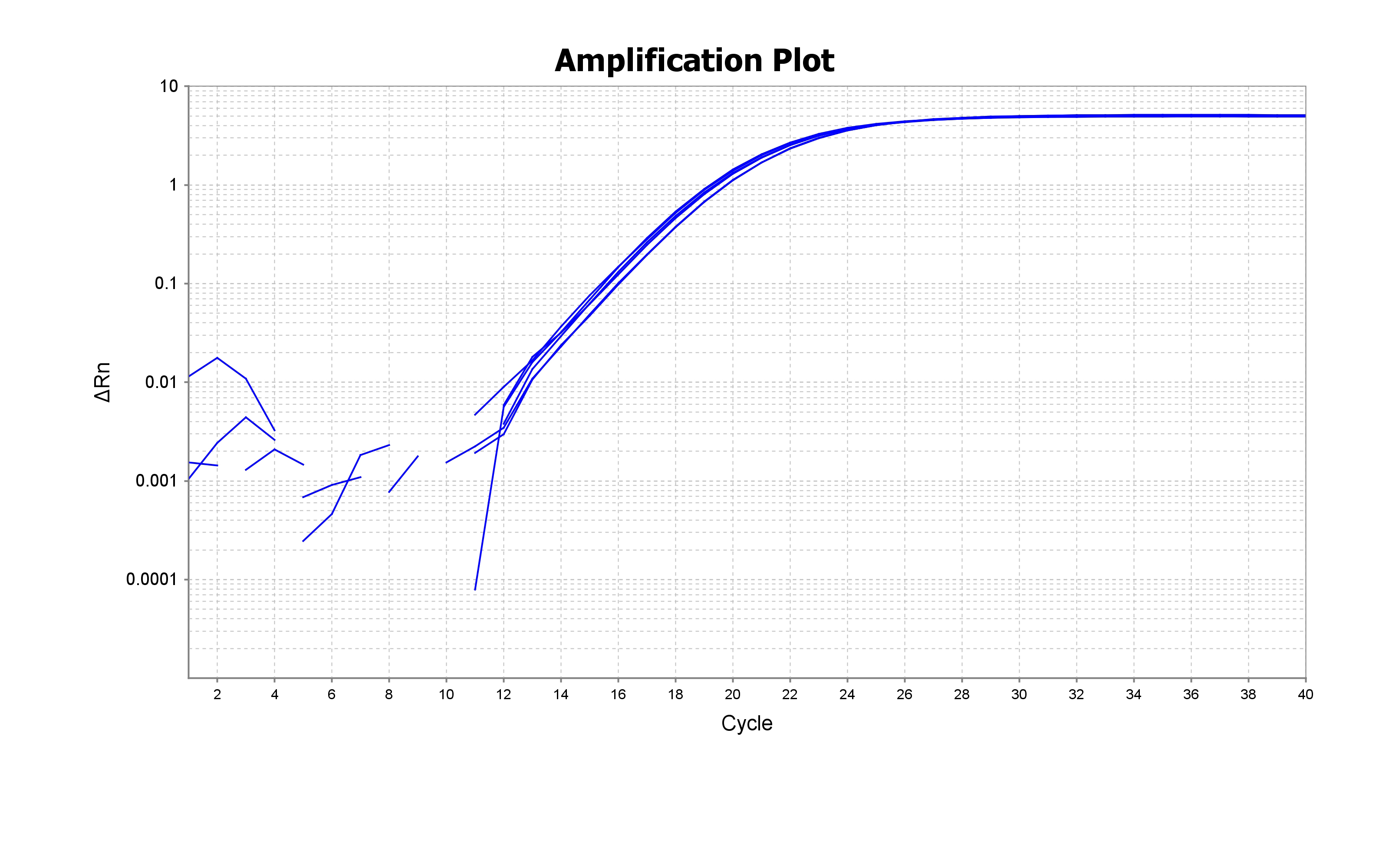

Supplement: Multimedia component 1 [file mmc1.zip › Western Blot and PCR raw data/Original/pcr/SMURF2/Amplification Plot-GAPDH.jpg]

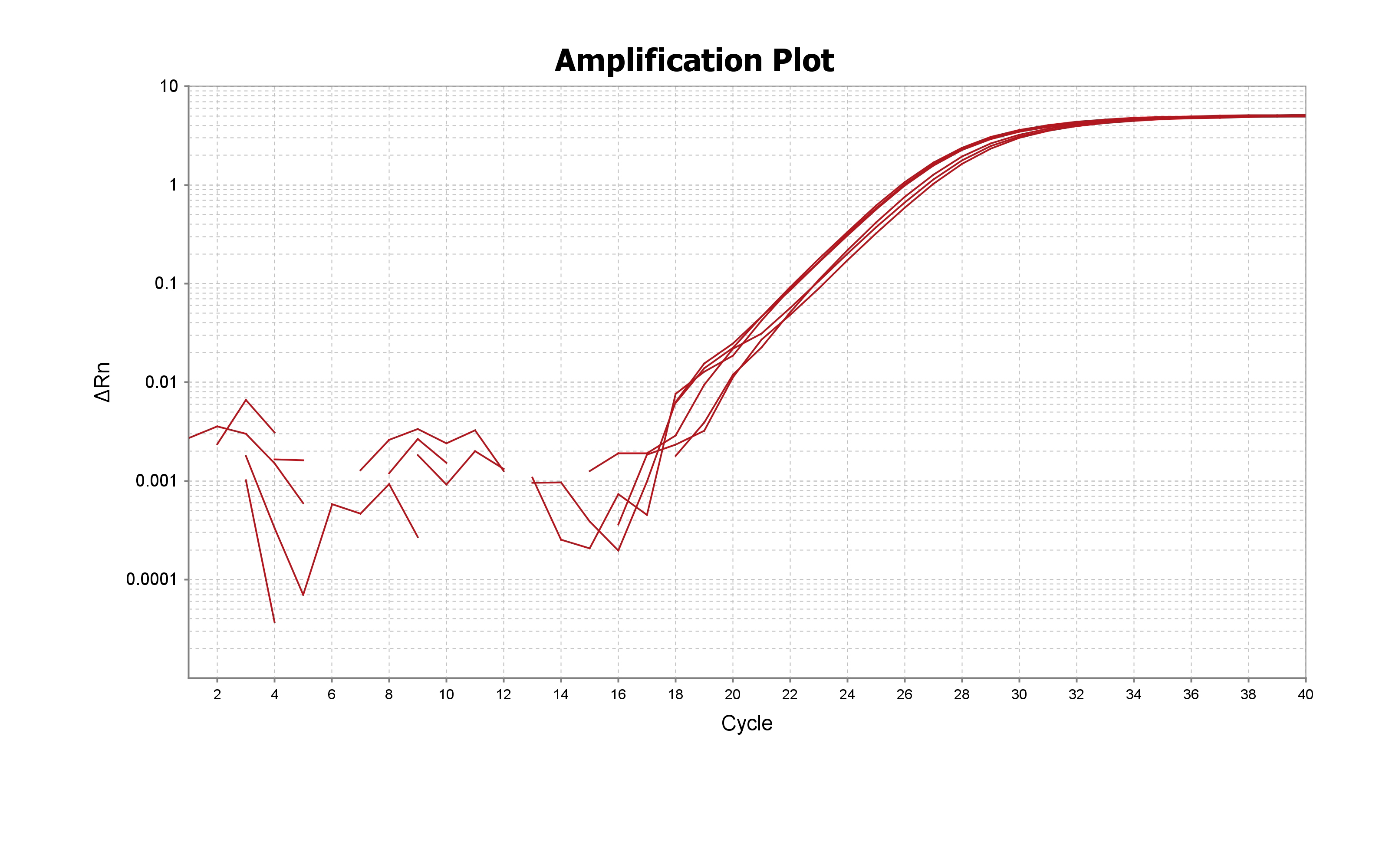

Supplement: Multimedia component 1 [file mmc1.zip › Western Blot and PCR raw data/Original/pcr/SMURF2/Amplification Plot-SMURF2.jpg]

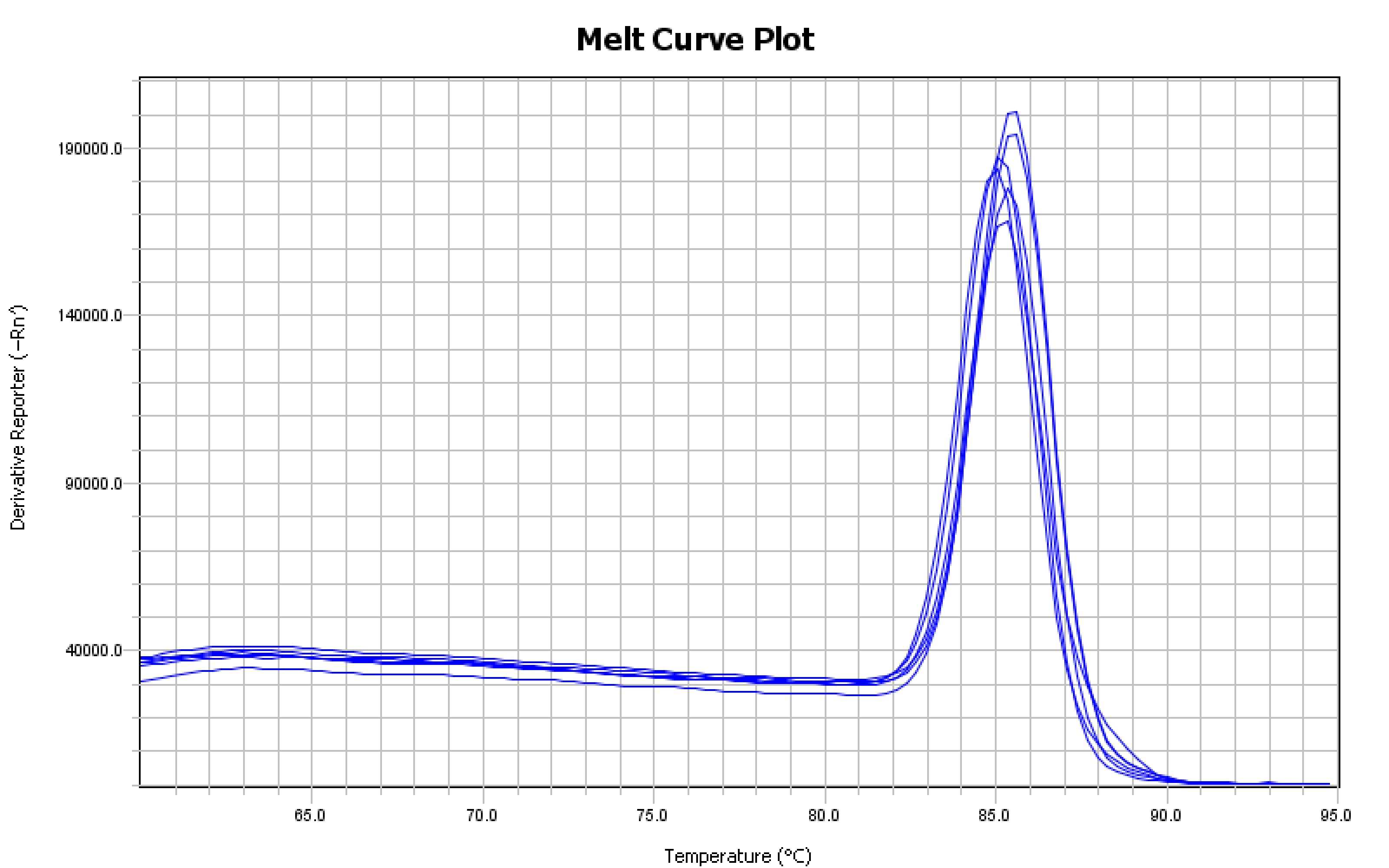

Supplement: Multimedia component 1 [file mmc1.zip › Western Blot and PCR raw data/Original/pcr/SMURF2/Melt Curve Plot-GAPDH.jpg]

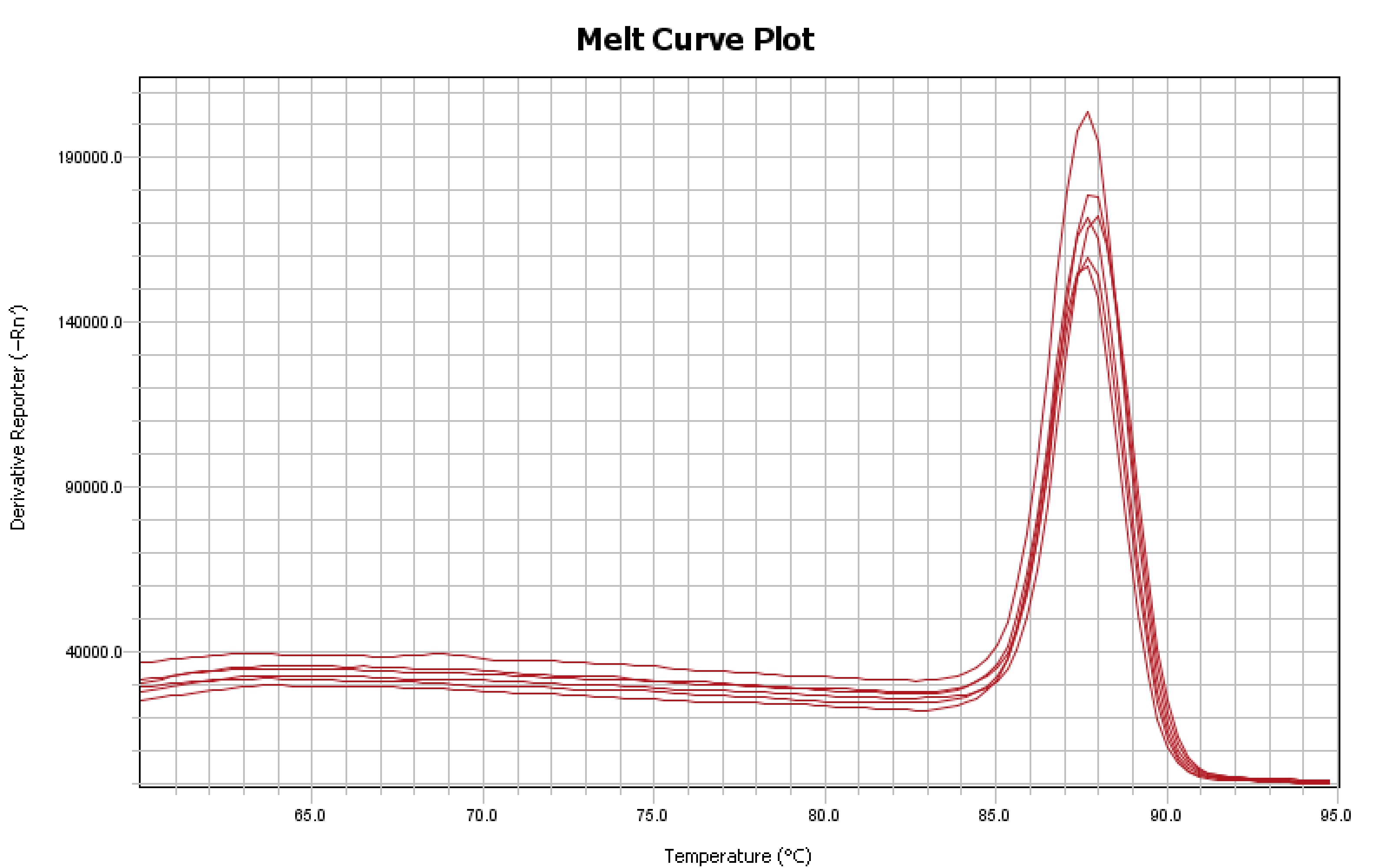

Supplement: Multimedia component 1 [file mmc1.zip › Western Blot and PCR raw data/Original/pcr/SMURF2/Melt Curve Plot-SMURF2.jpg]

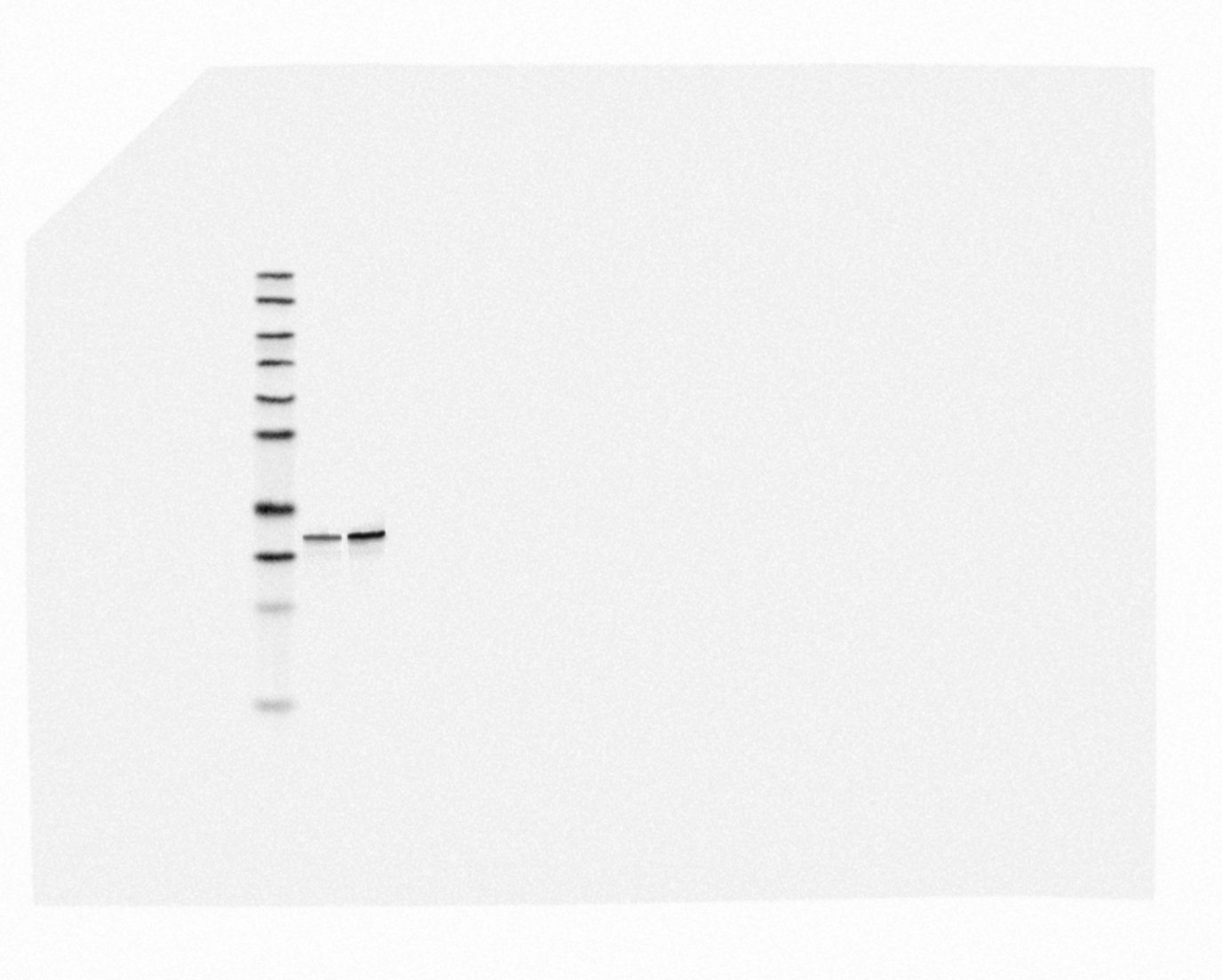

Supplement: Multimedia component 1 [file mmc1.zip › Western Blot and PCR raw data/Original/WB/原图/CCDC80-1.jpg]

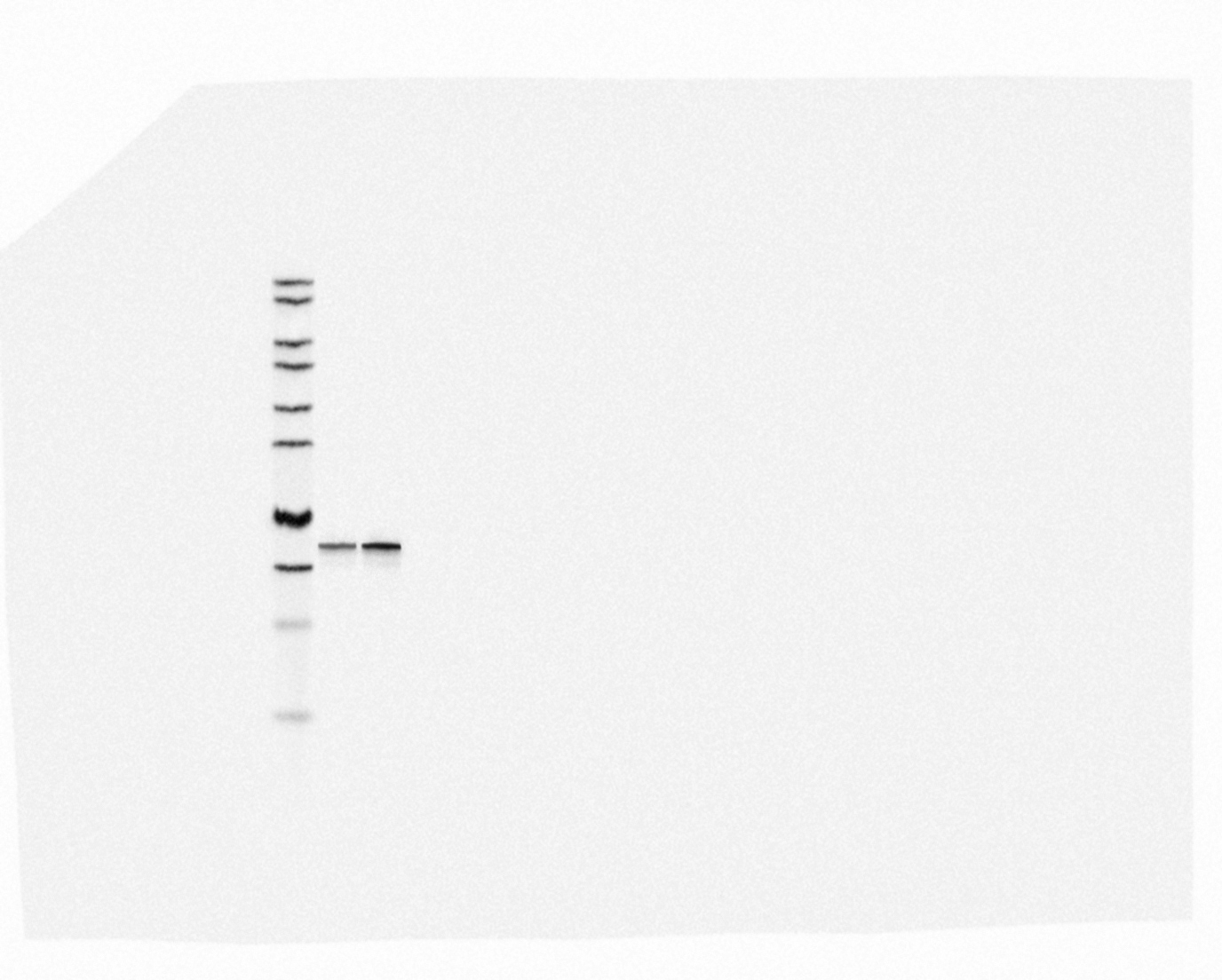

Supplement: Multimedia component 1 [file mmc1.zip › Western Blot and PCR raw data/Original/WB/原图/CCDC80-2.jpg]

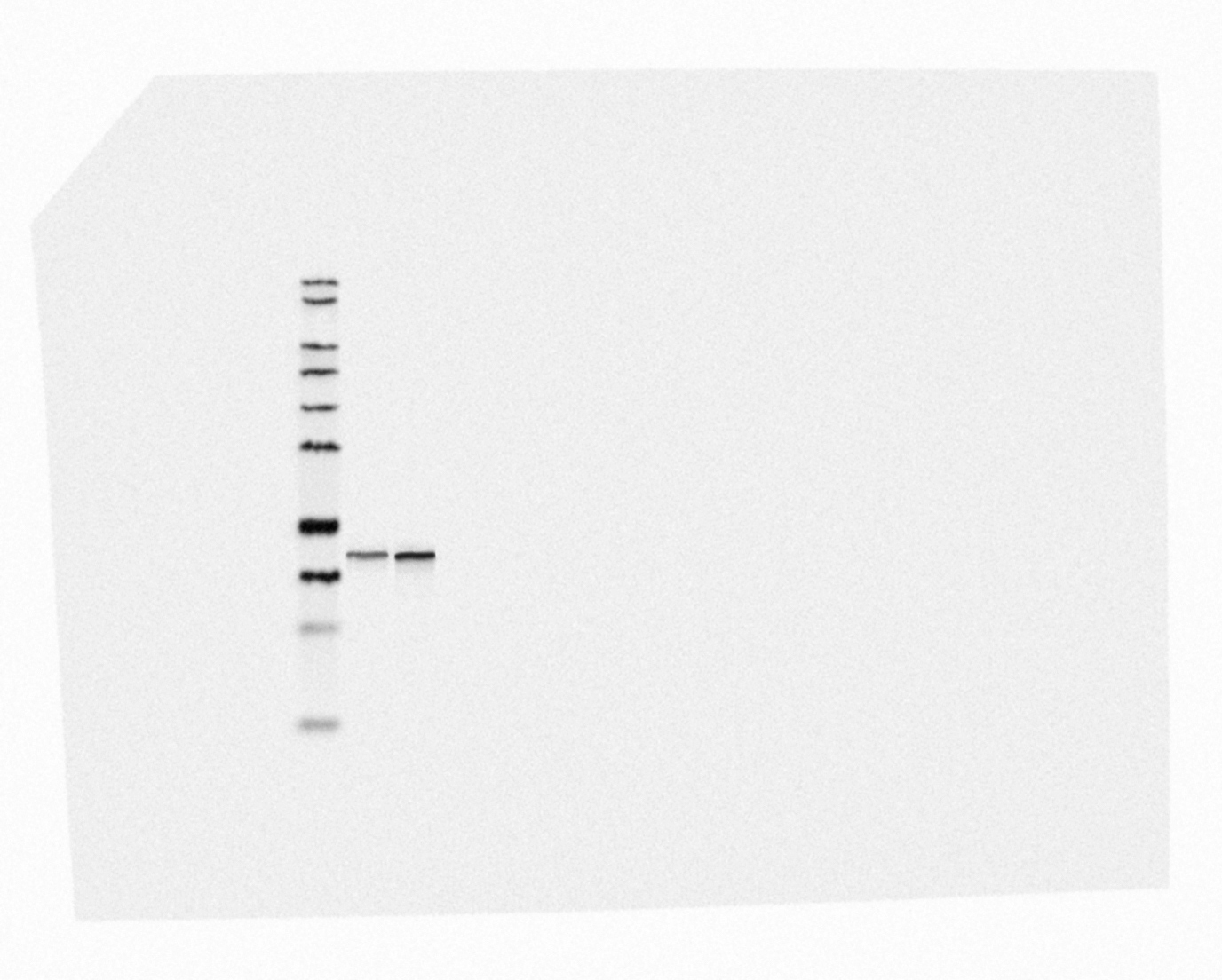

Supplement: Multimedia component 1 [file mmc1.zip › Western Blot and PCR raw data/Original/WB/原图/CCDC80-3.jpg]

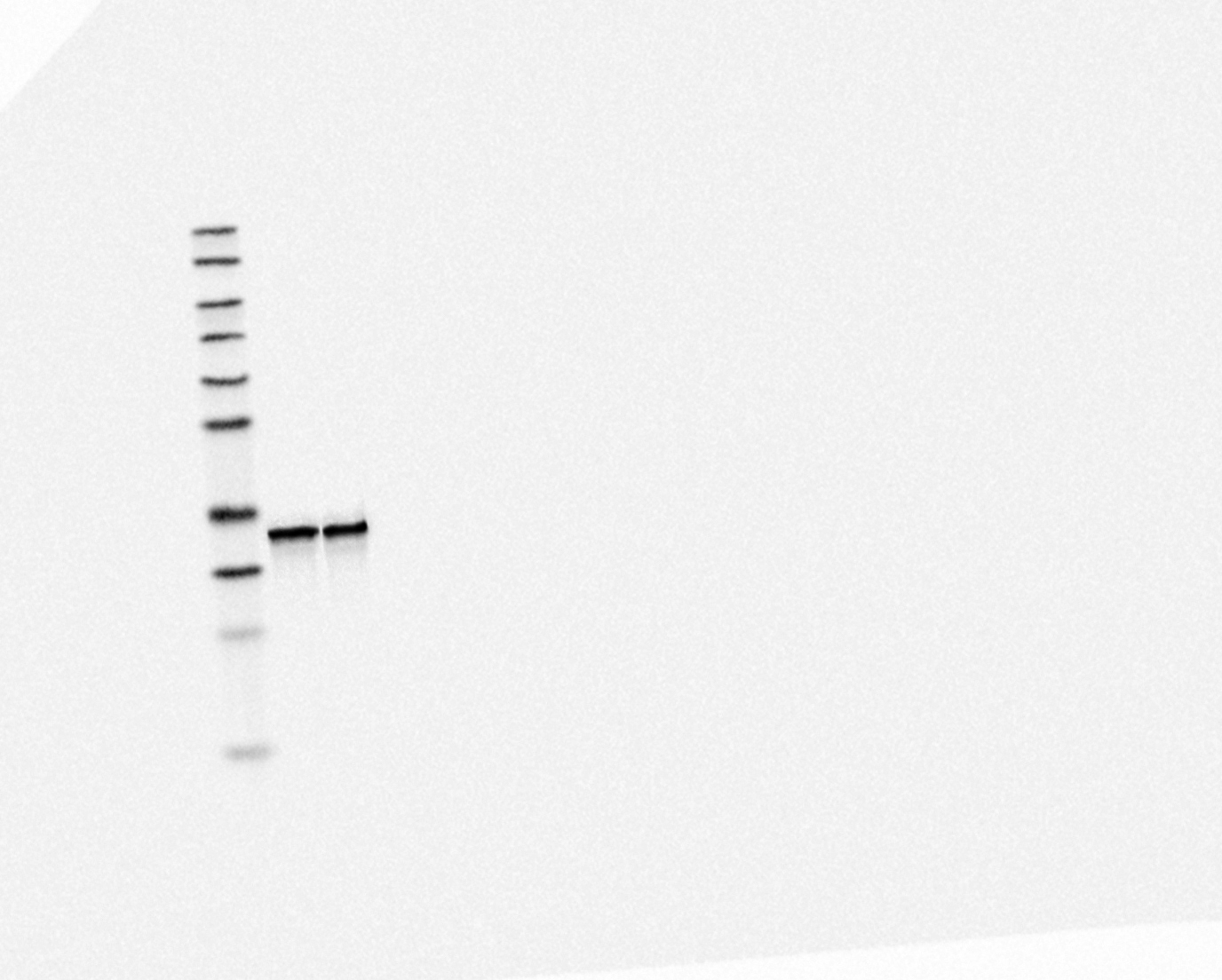

Supplement: Multimedia component 1 [file mmc1.zip › Western Blot and PCR raw data/Original/WB/原图/GAPDH-1.jpg]

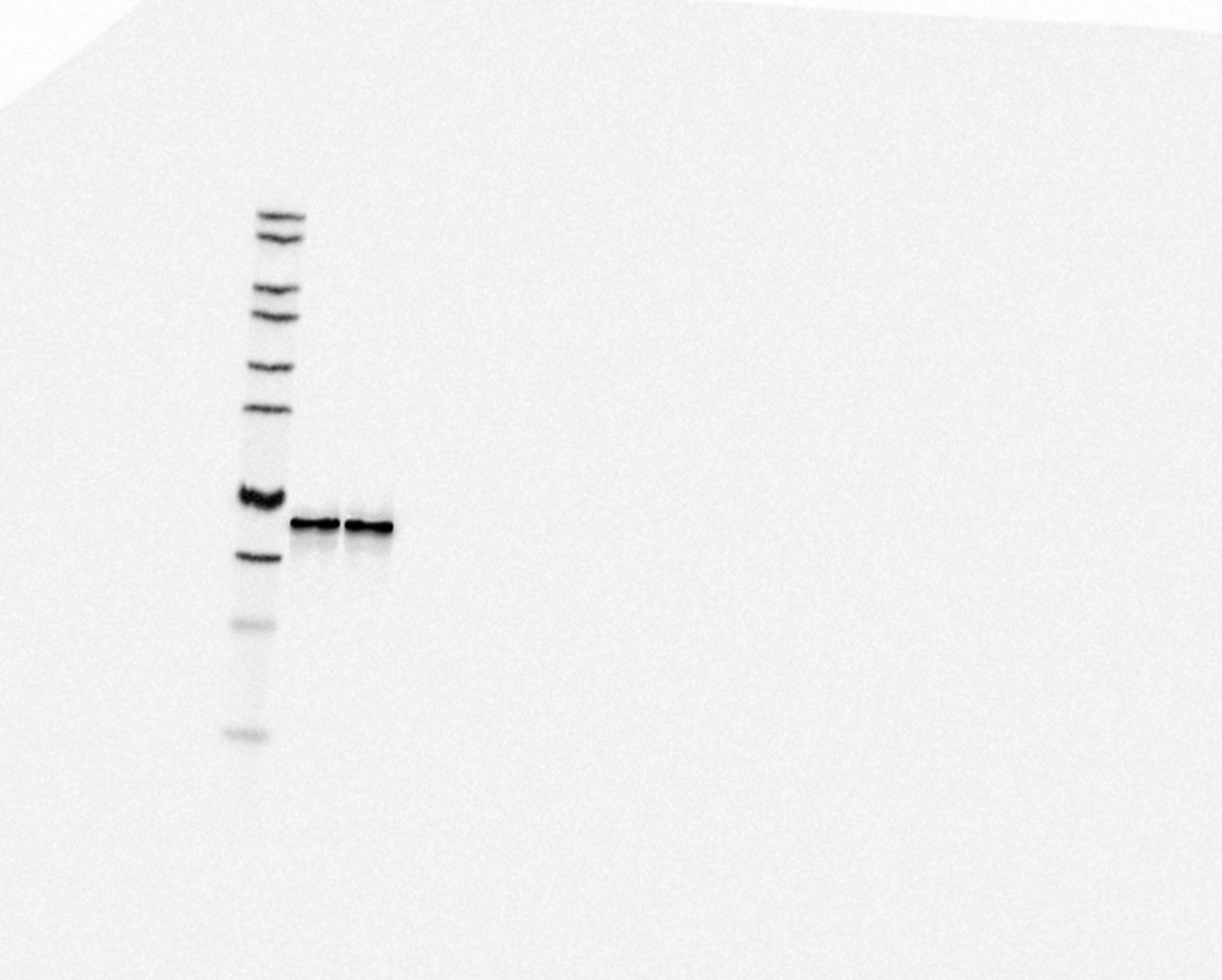

Supplement: Multimedia component 1 [file mmc1.zip › Western Blot and PCR raw data/Original/WB/原图/GAPDH-2.jpg]

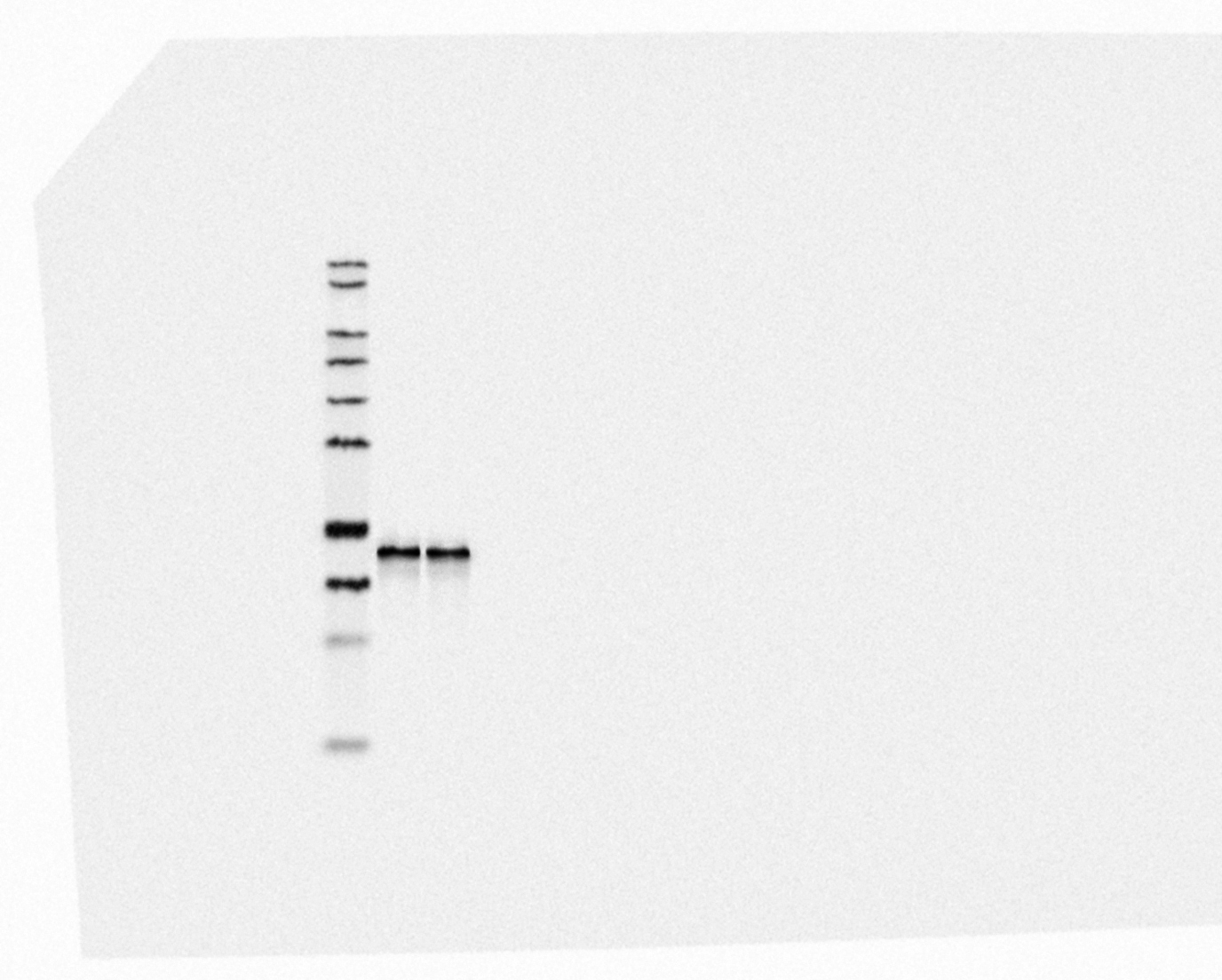

Supplement: Multimedia component 1 [file mmc1.zip › Western Blot and PCR raw data/Original/WB/原图/GAPDH-3.jpg]

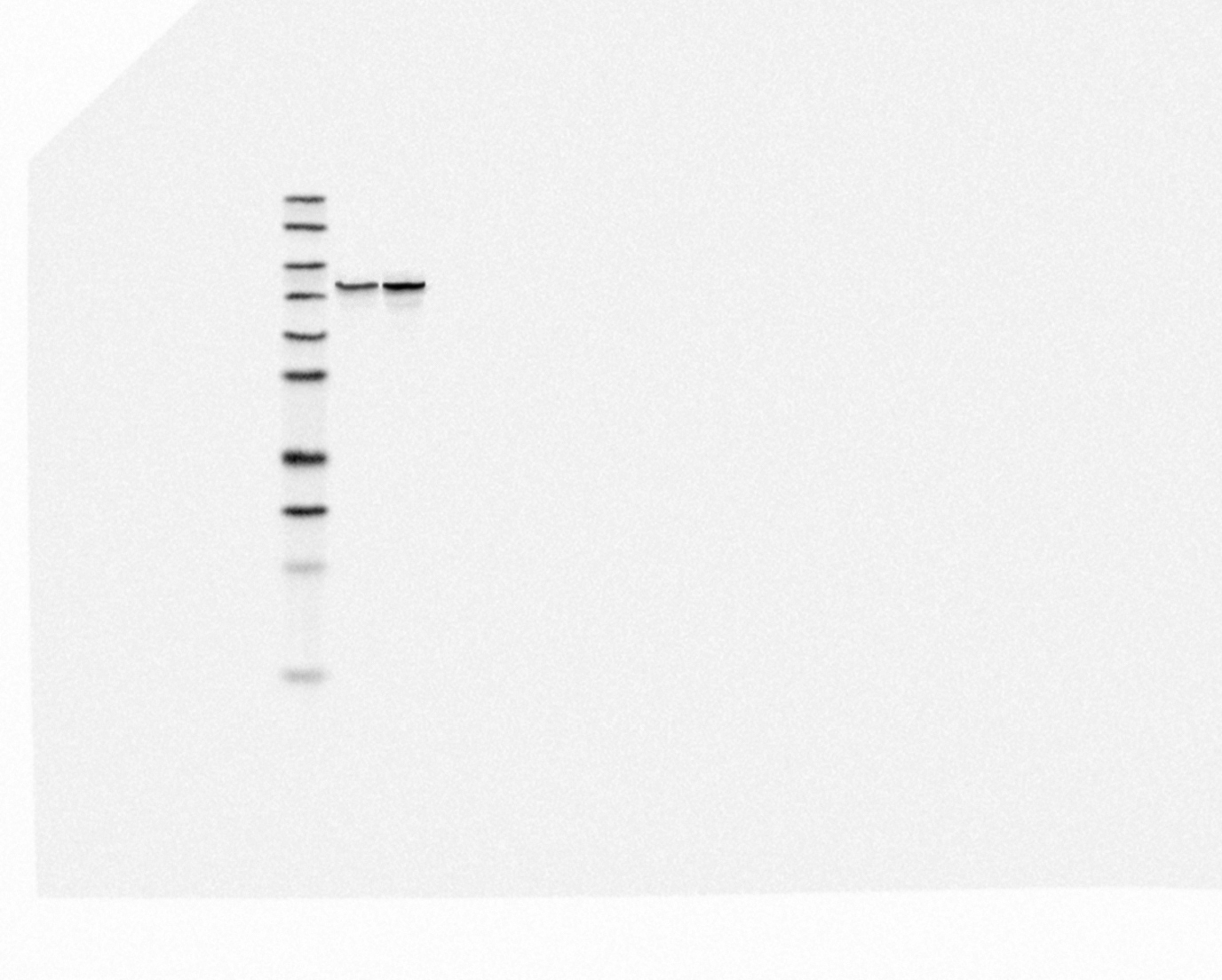

Supplement: Multimedia component 1 [file mmc1.zip › Western Blot and PCR raw data/Original/WB/原图/SMURF2-1.jpg]

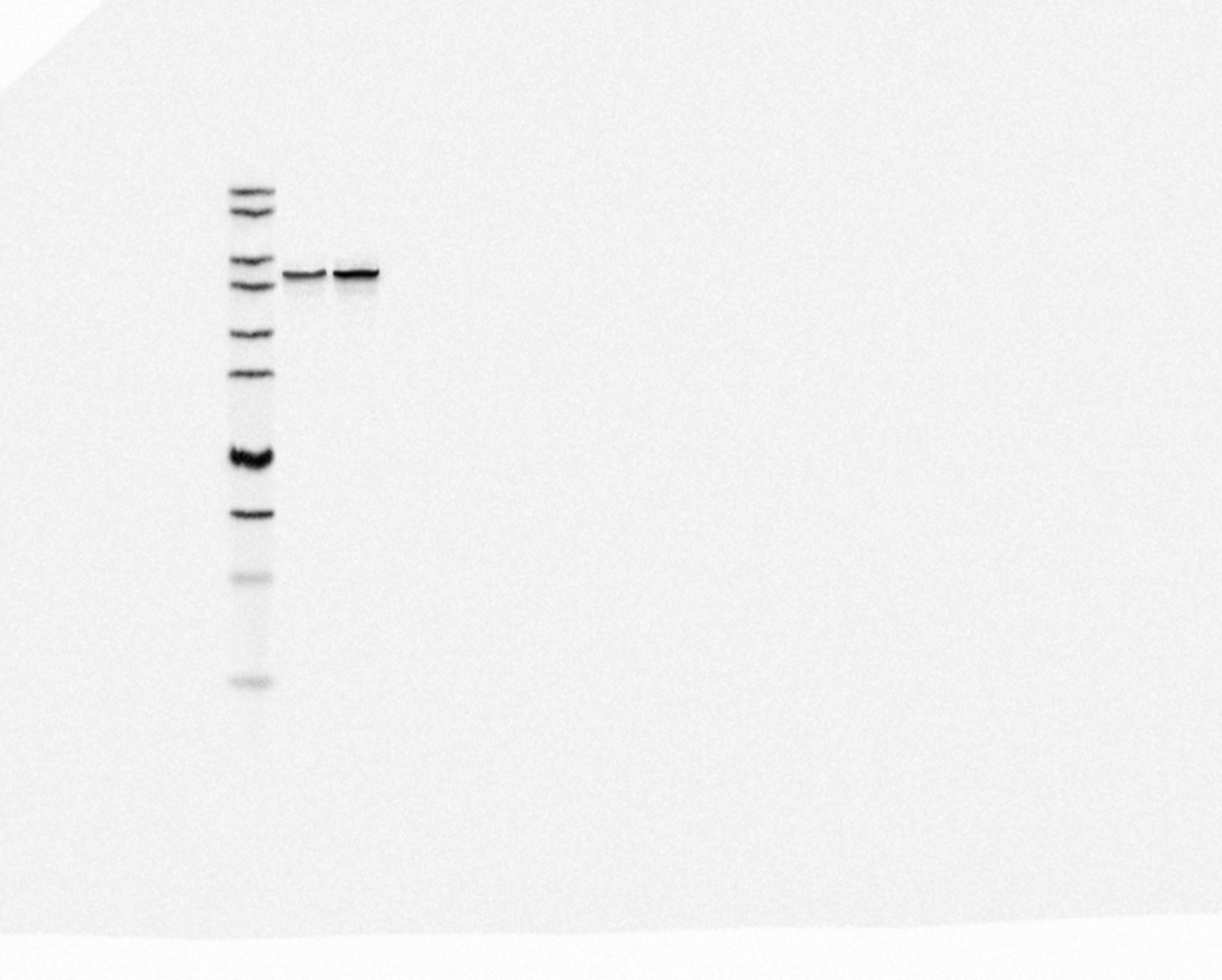

Supplement: Multimedia component 1 [file mmc1.zip › Western Blot and PCR raw data/Original/WB/原图/SMURF2-2.jpg]

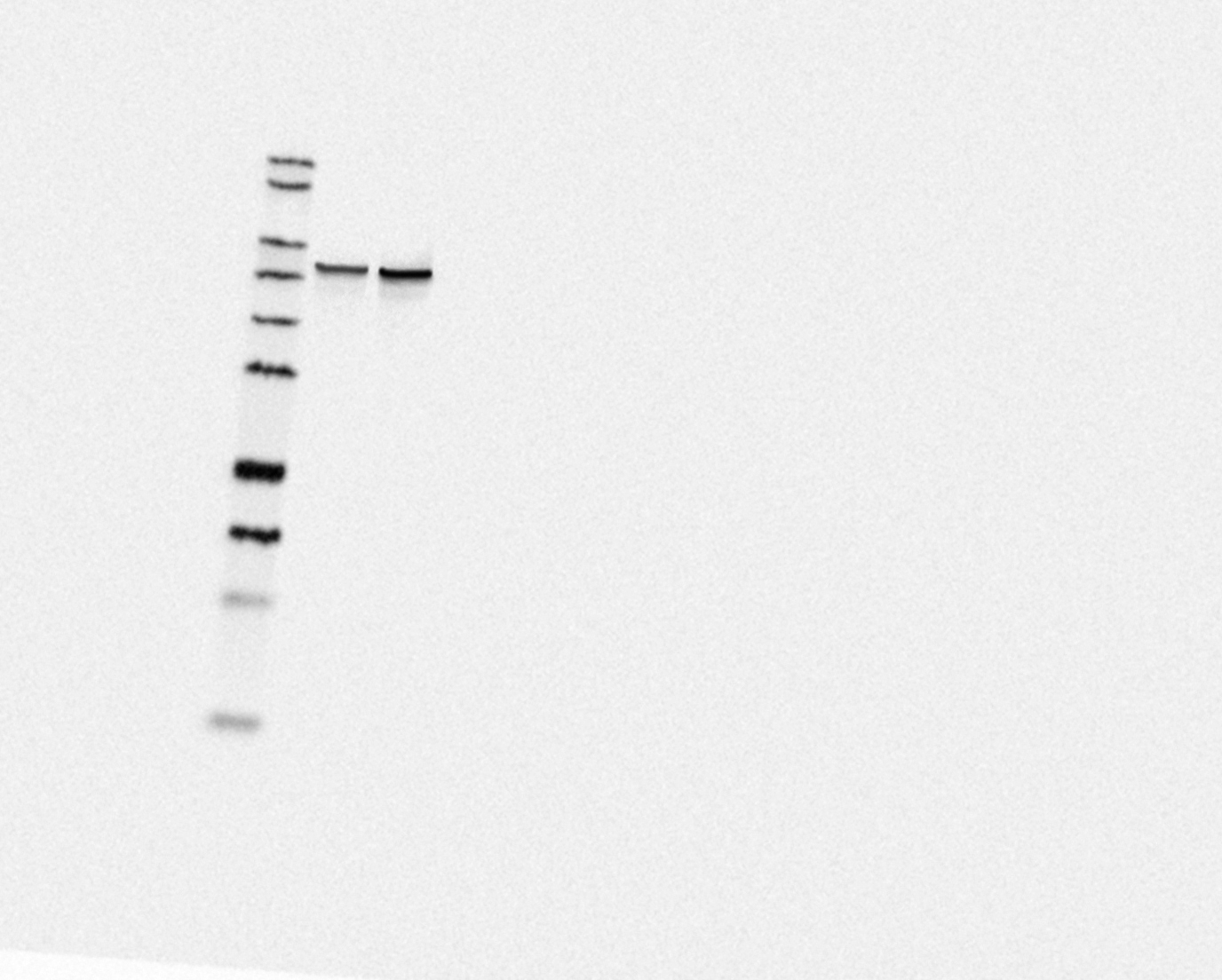

Supplement: Multimedia component 1 [file mmc1.zip › Western Blot and PCR raw data/Original/WB/原图/SMURF2-3.jpg]

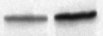

Supplement: Multimedia component 1 [file mmc1.zip › Western Blot and PCR raw data/Original/WB/裁剪/CCDC80-1.jpg]

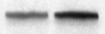

Supplement: Multimedia component 1 [file mmc1.zip › Western Blot and PCR raw data/Original/WB/裁剪/CCDC80-2.jpg]

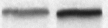

Supplement: Multimedia component 1 [file mmc1.zip › Western Blot and PCR raw data/Original/WB/裁剪/CCDC80-3.jpg]

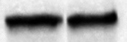

Supplement: Multimedia component 1 [file mmc1.zip › Western Blot and PCR raw data/Original/WB/裁剪/GAPDH-1.jpg]

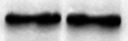

Supplement: Multimedia component 1 [file mmc1.zip › Western Blot and PCR raw data/Original/WB/裁剪/GAPDH-2.jpg]

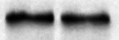

Supplement: Multimedia component 1 [file mmc1.zip › Western Blot and PCR raw data/Original/WB/裁剪/GAPDH-3.jpg]

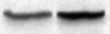

Supplement: Multimedia component 1 [file mmc1.zip › Western Blot and PCR raw data/Original/WB/裁剪/SMURF2-1.jpg]

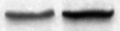

Supplement: Multimedia component 1 [file mmc1.zip › Western Blot and PCR raw data/Original/WB/裁剪/SMURF2-2.jpg]

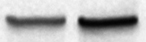

Supplement: Multimedia component 1 [file mmc1.zip › Western Blot and PCR raw data/Original/WB/裁剪/SMURF2-3.jpg]

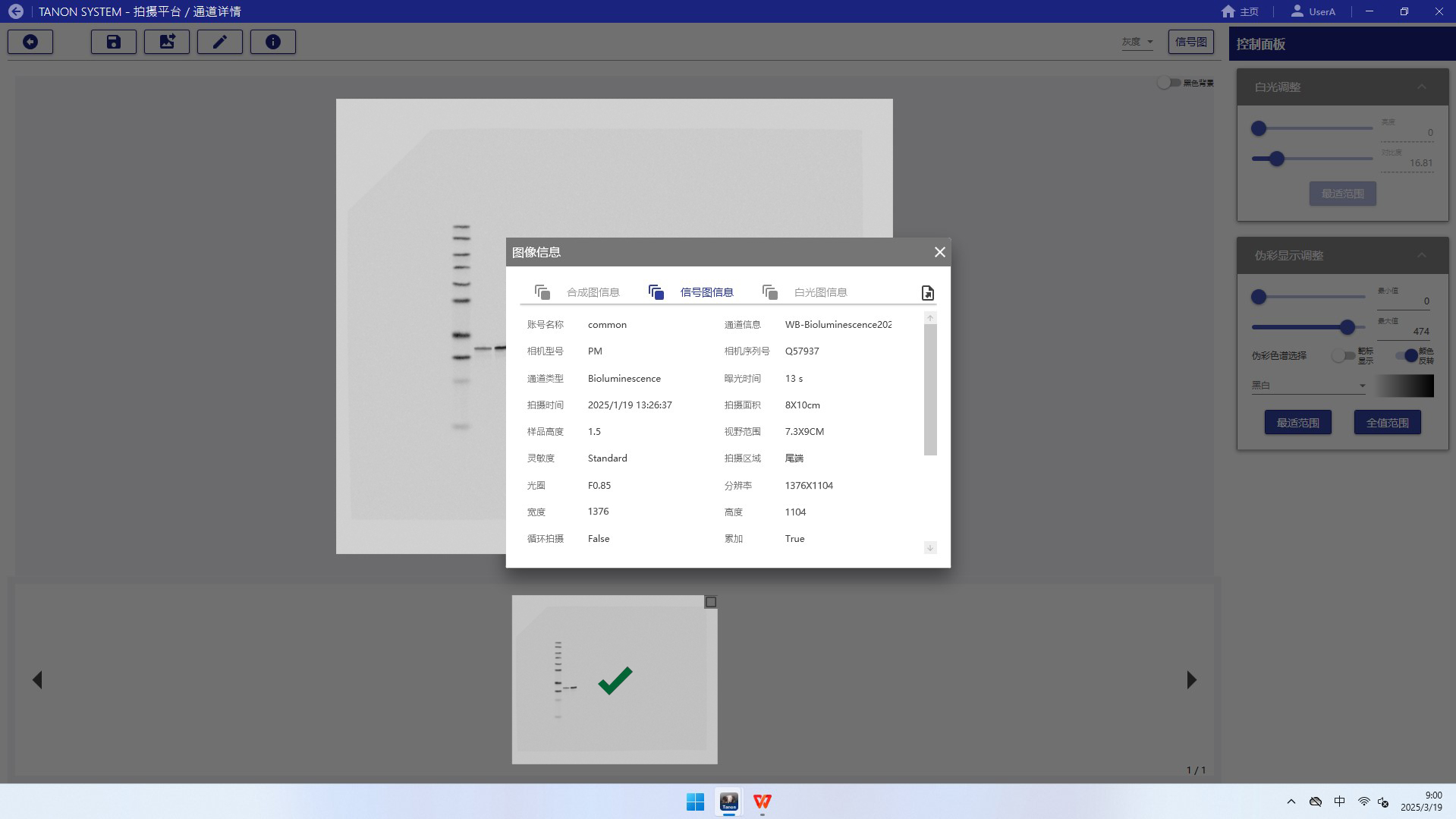

Supplement: Multimedia component 1 [file mmc1.zip › Western Blot and PCR raw data/Screen capture/CCDC80/1.jpg]

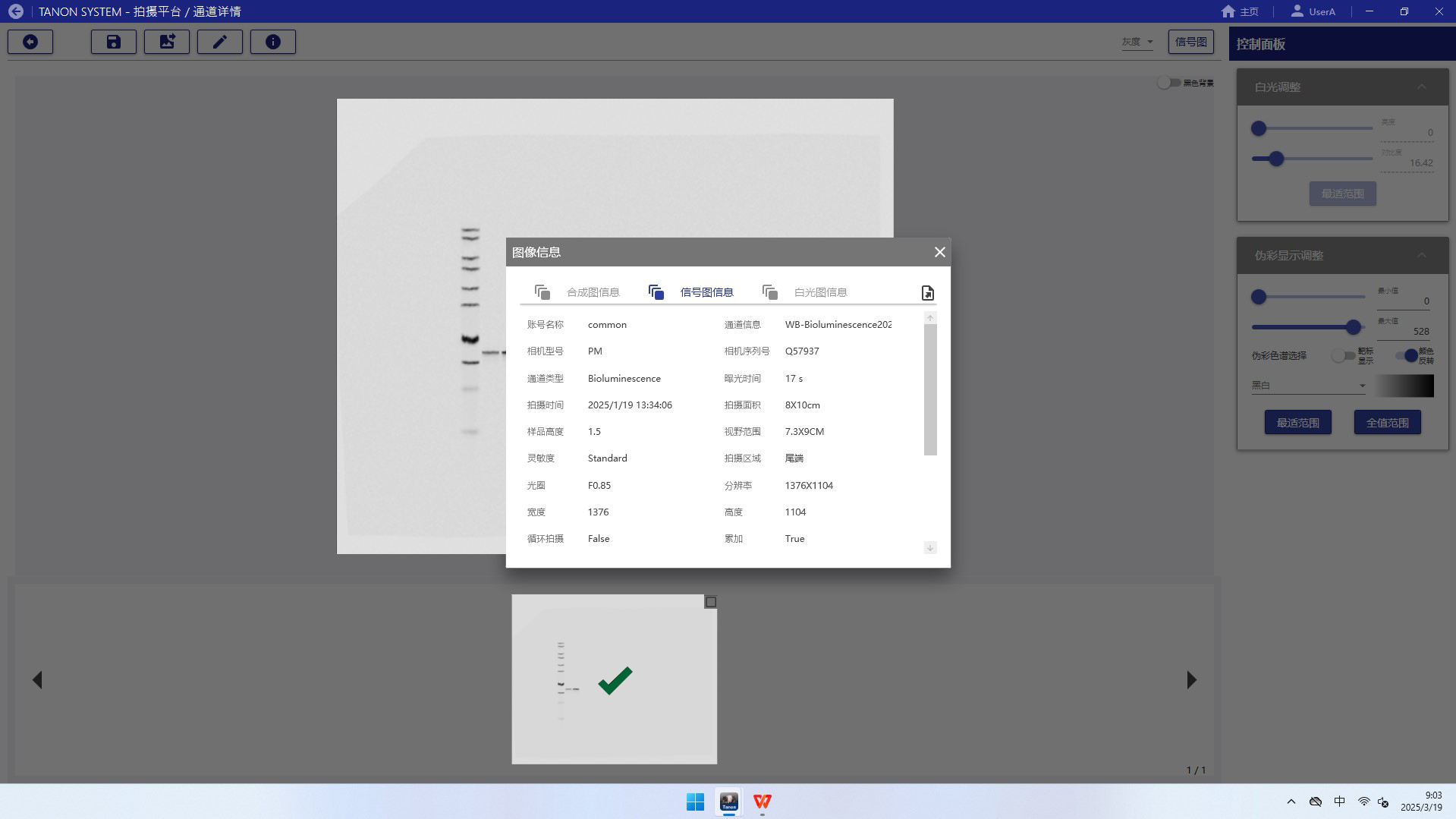

Supplement: Multimedia component 1 [file mmc1.zip › Western Blot and PCR raw data/Screen capture/CCDC80/2.jpg]

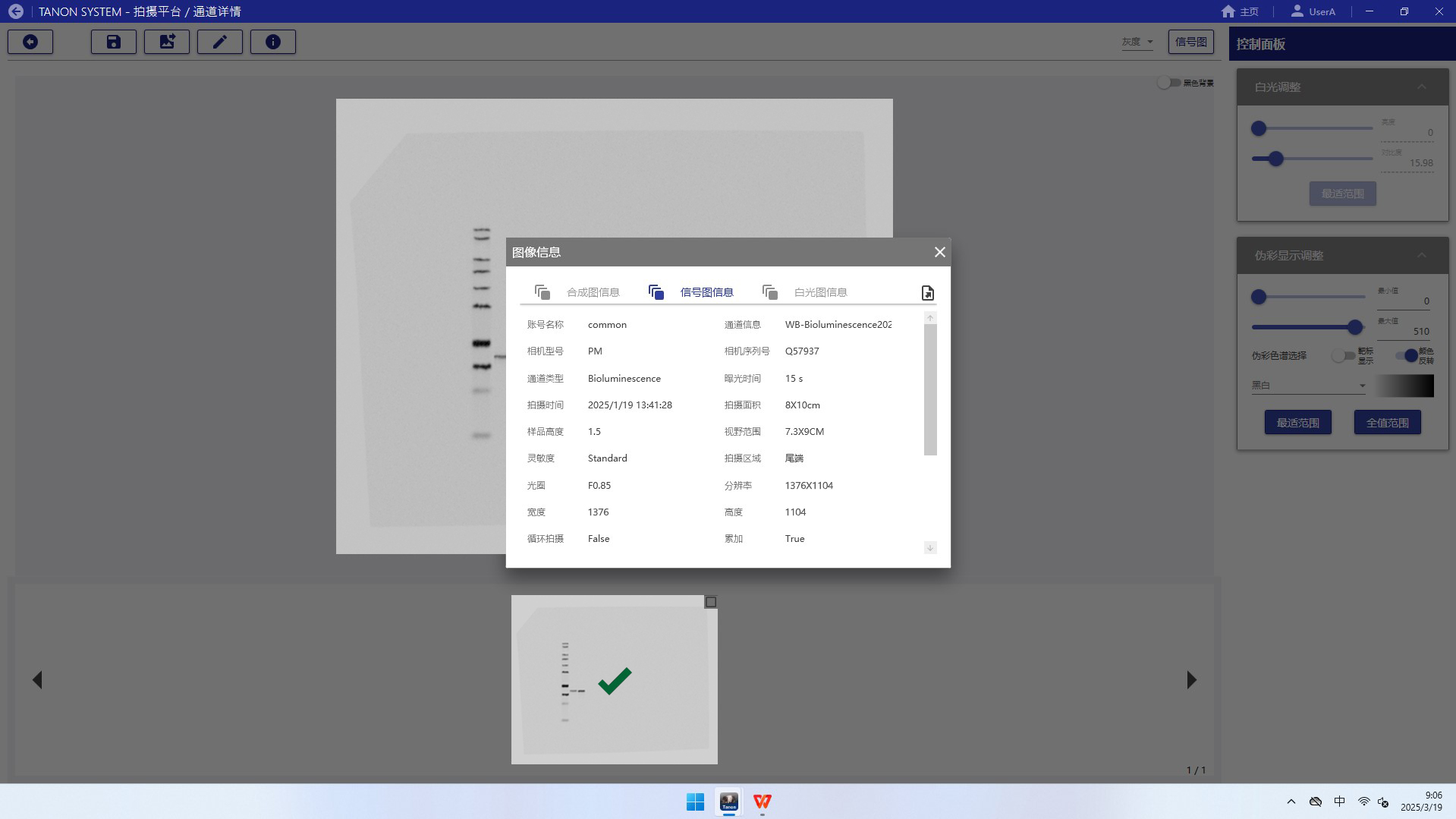

Supplement: Multimedia component 1 [file mmc1.zip › Western Blot and PCR raw data/Screen capture/CCDC80/3.jpg]

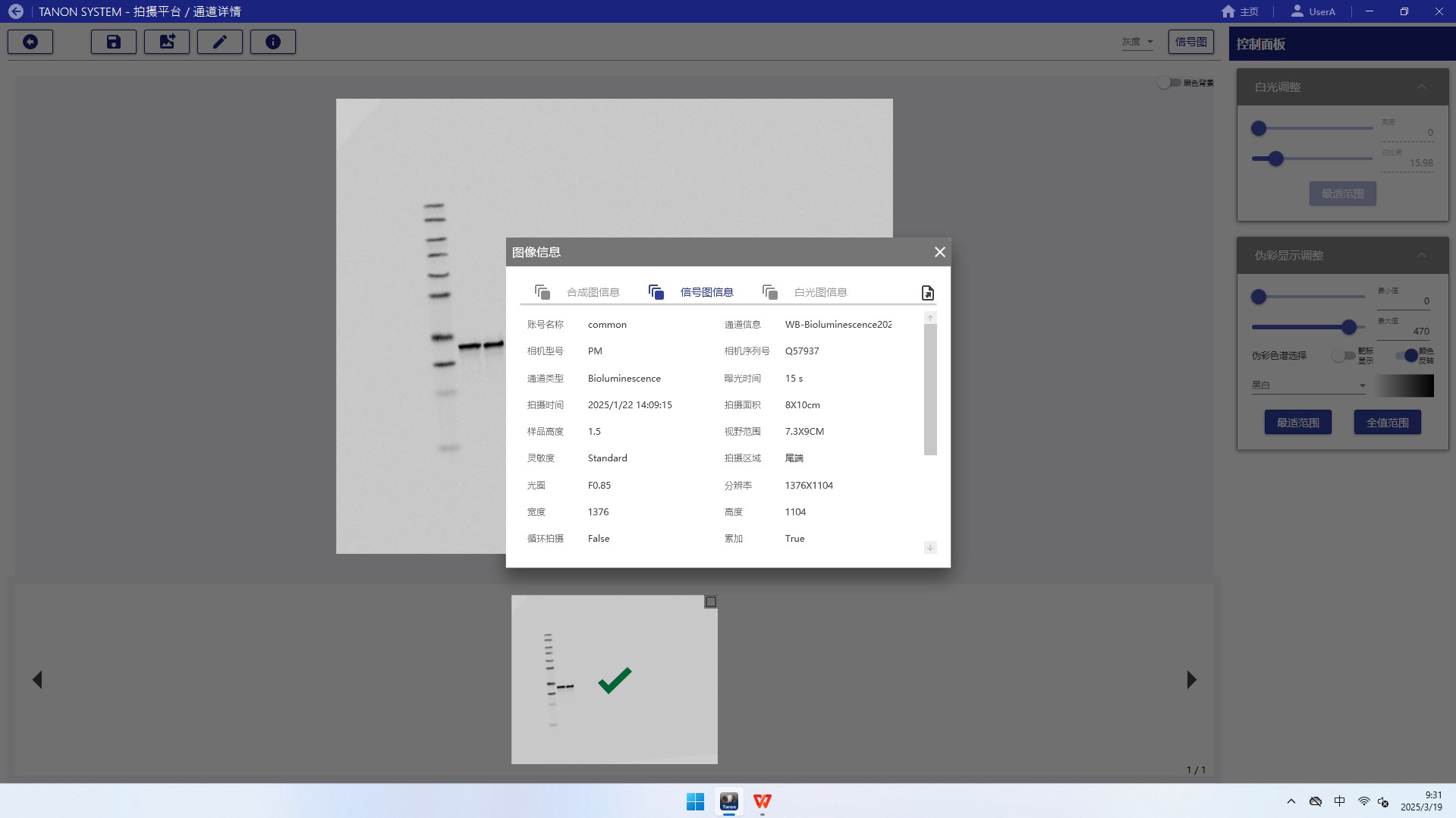

Supplement: Multimedia component 1 [file mmc1.zip › Western Blot and PCR raw data/Screen capture/GAPDH/1.jpg]

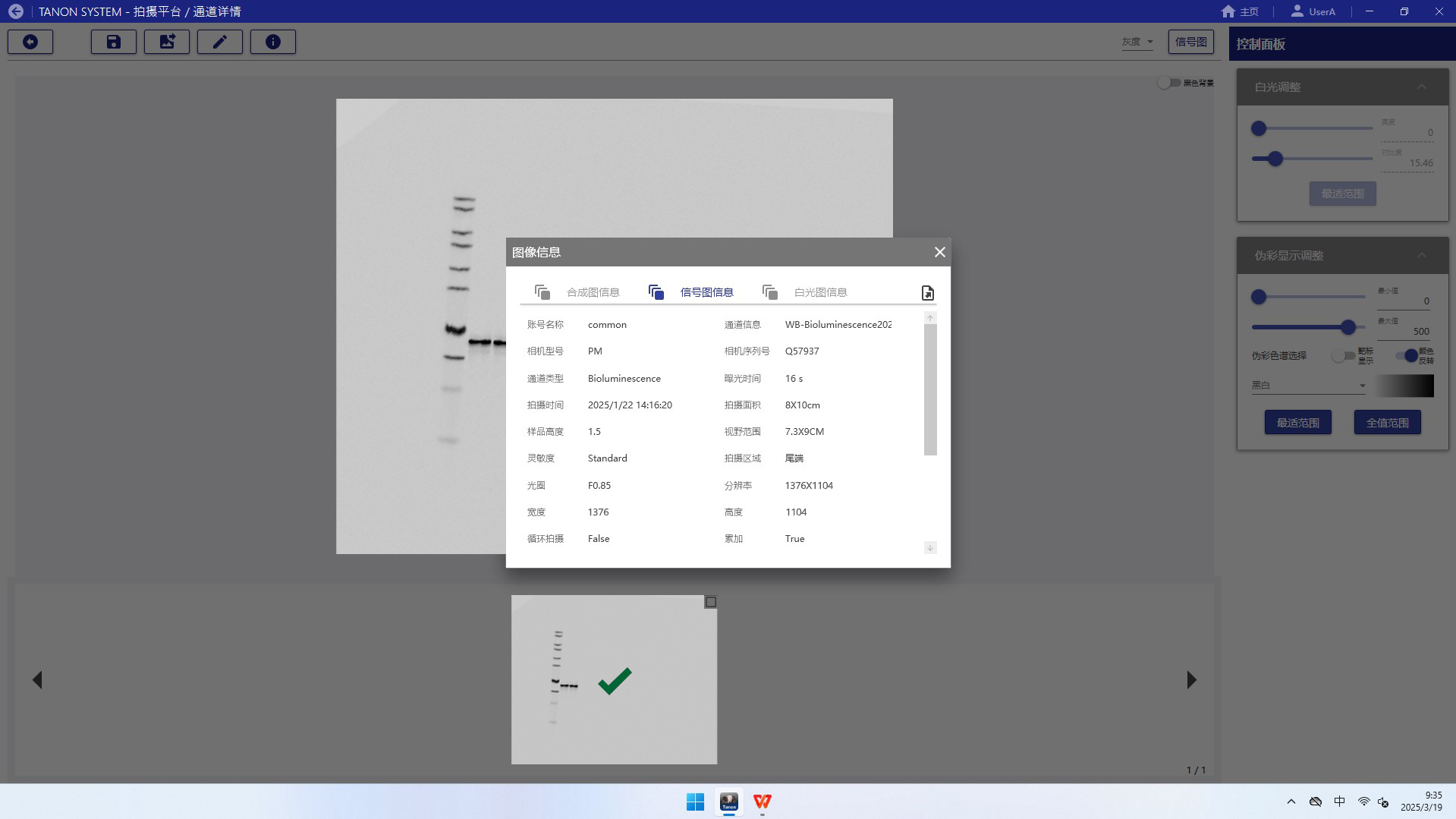

Supplement: Multimedia component 1 [file mmc1.zip › Western Blot and PCR raw data/Screen capture/GAPDH/2.jpg]

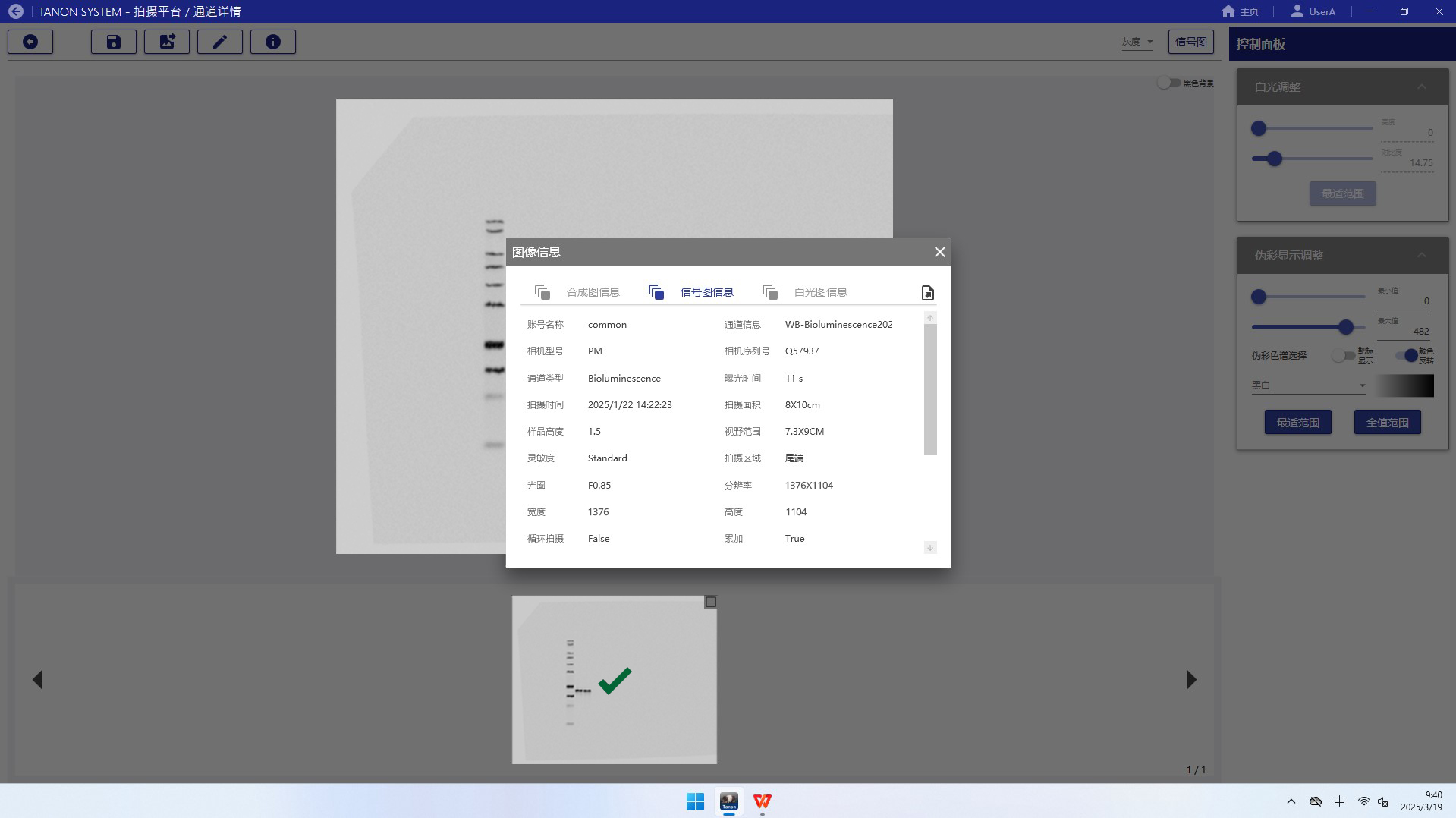

Supplement: Multimedia component 1 [file mmc1.zip › Western Blot and PCR raw data/Screen capture/GAPDH/3.jpg]

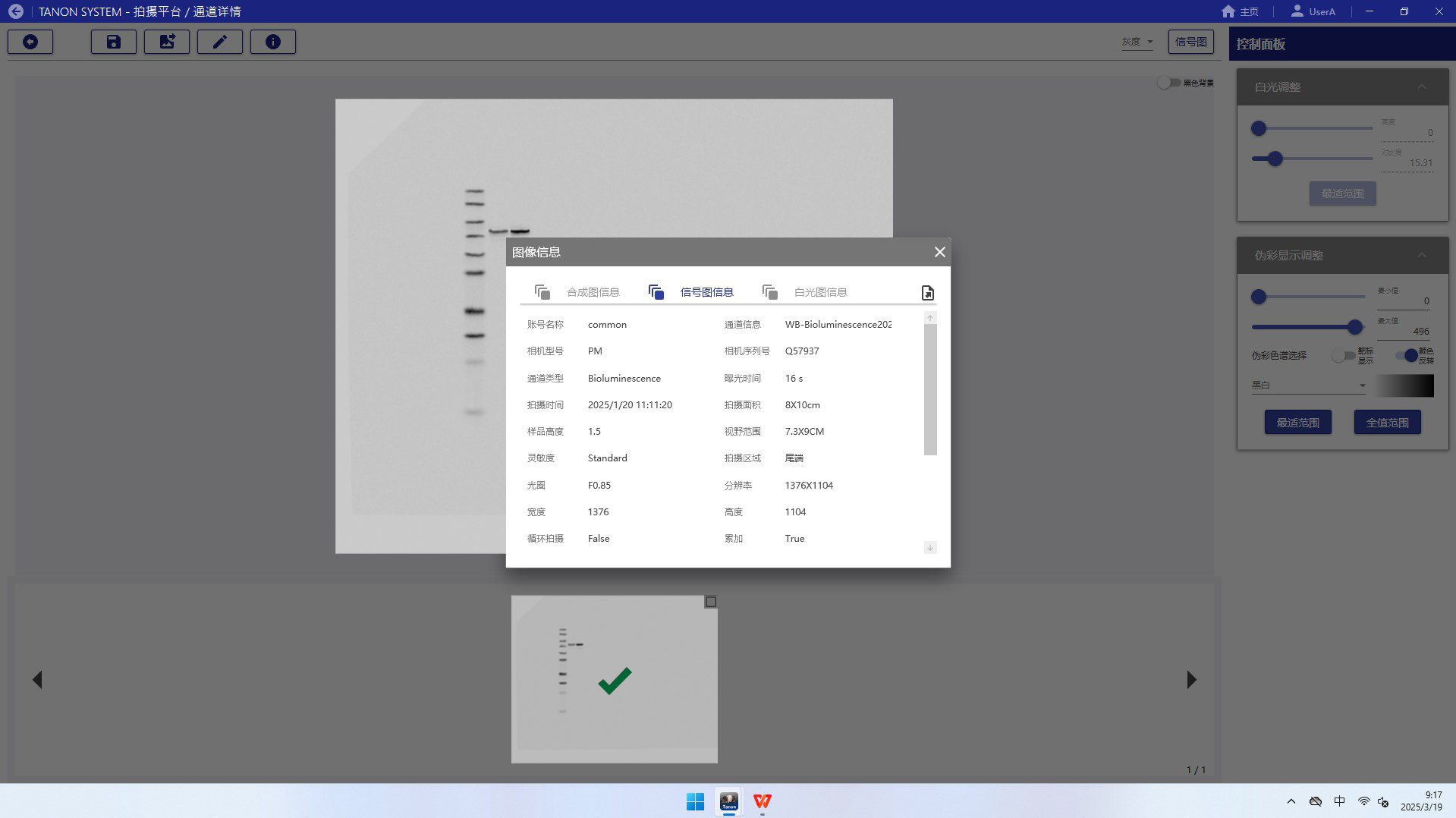

Supplement: Multimedia component 1 [file mmc1.zip › Western Blot and PCR raw data/Screen capture/SMURF2/1.jpg]

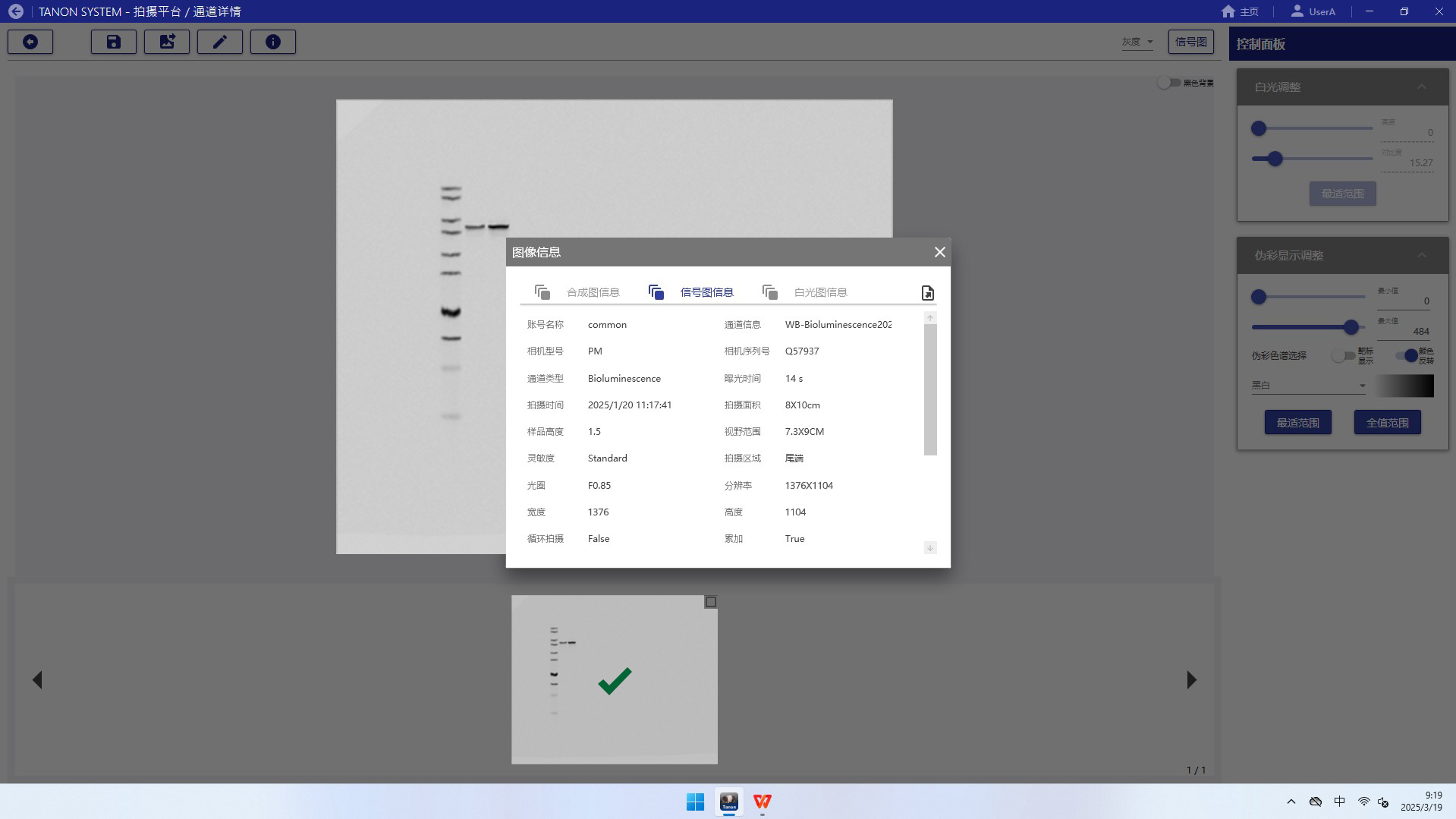

Supplement: Multimedia component 1 [file mmc1.zip › Western Blot and PCR raw data/Screen capture/SMURF2/2.jpg]

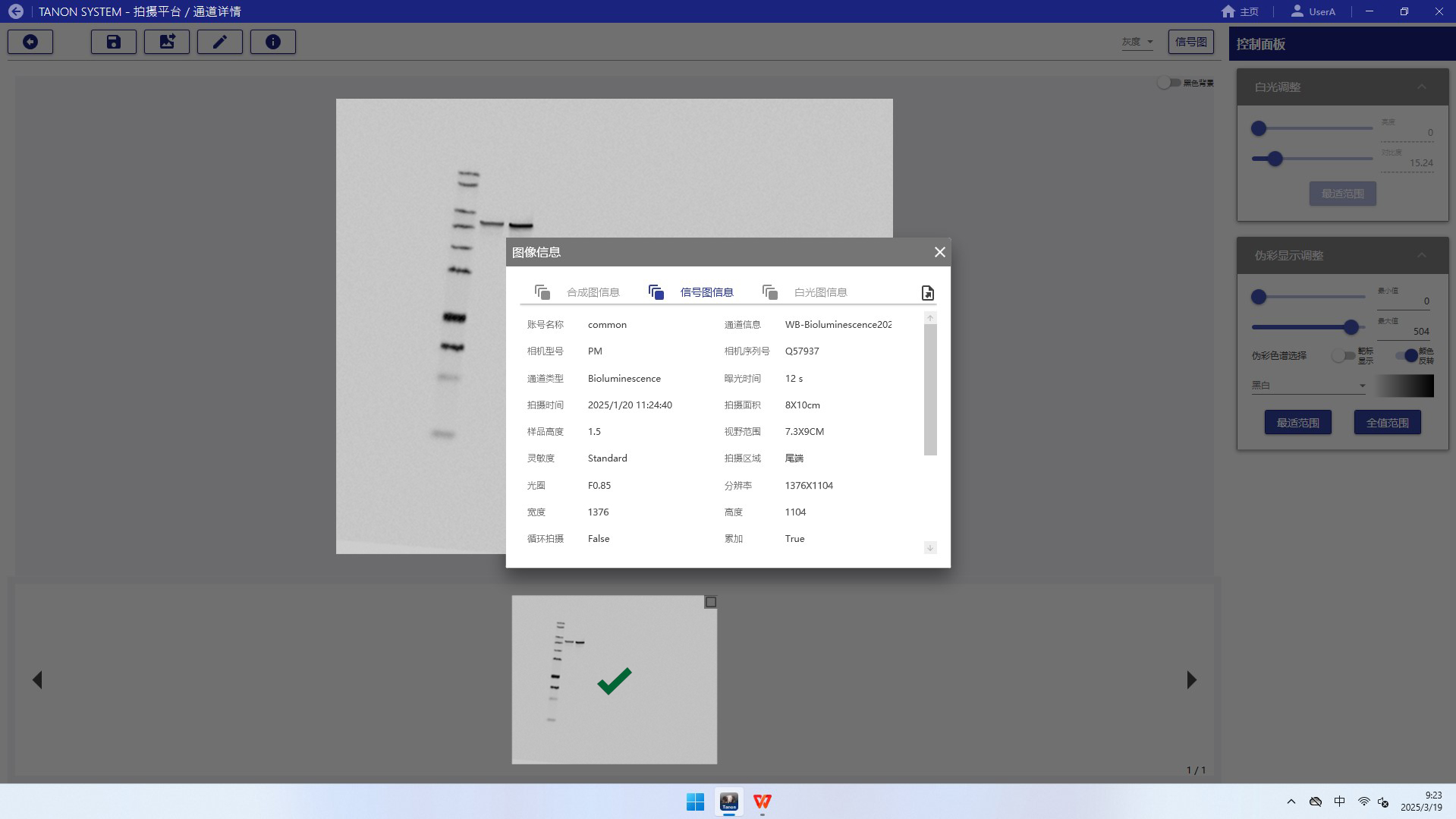

Supplement: Multimedia component 1 [file mmc1.zip › Western Blot and PCR raw data/Screen capture/SMURF2/3.jpg]

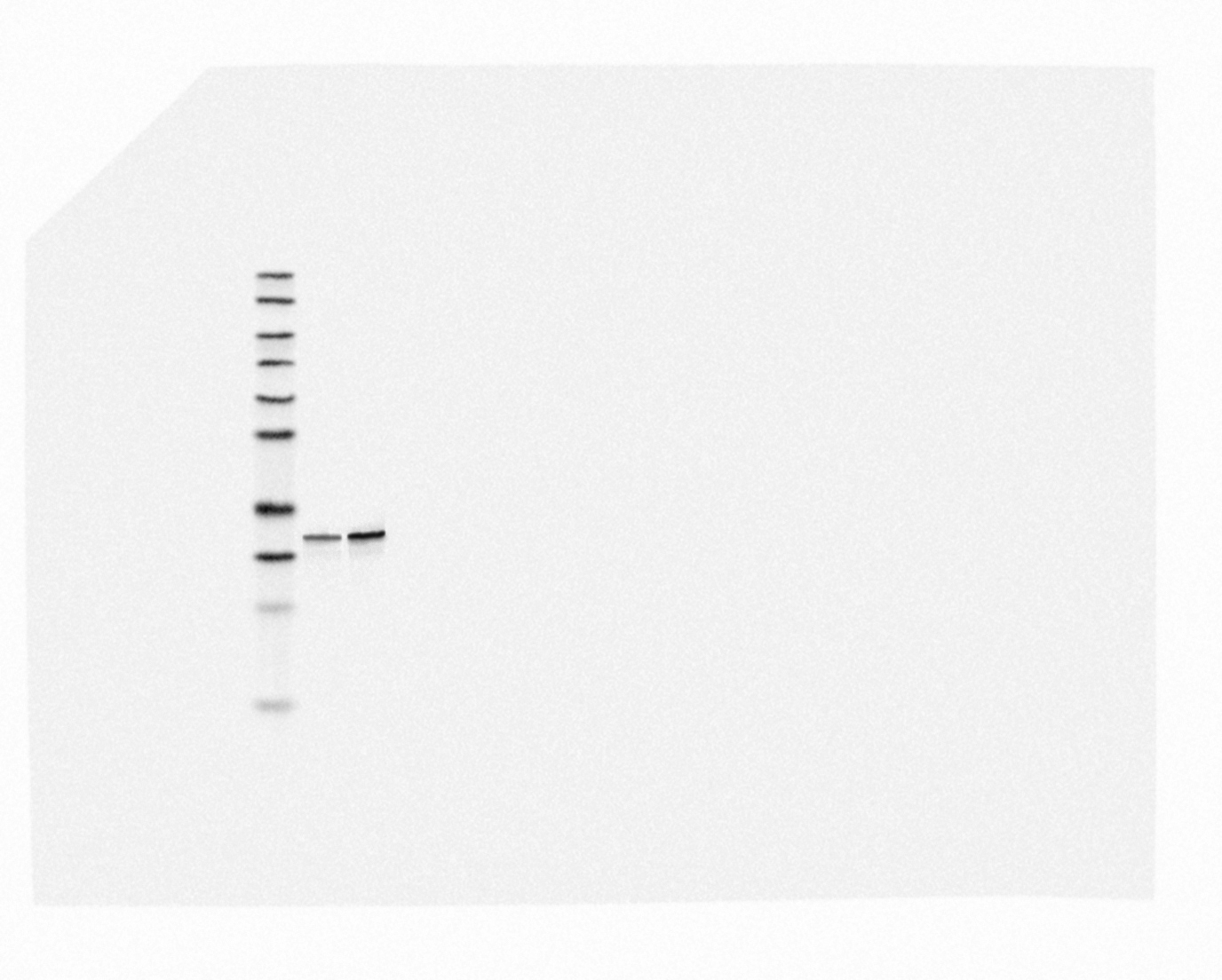

Supplement: Multimedia component 1 [file mmc1.zip › Western Blot and PCR raw data/Screen capture/TIF/CCDC80-1.tif]

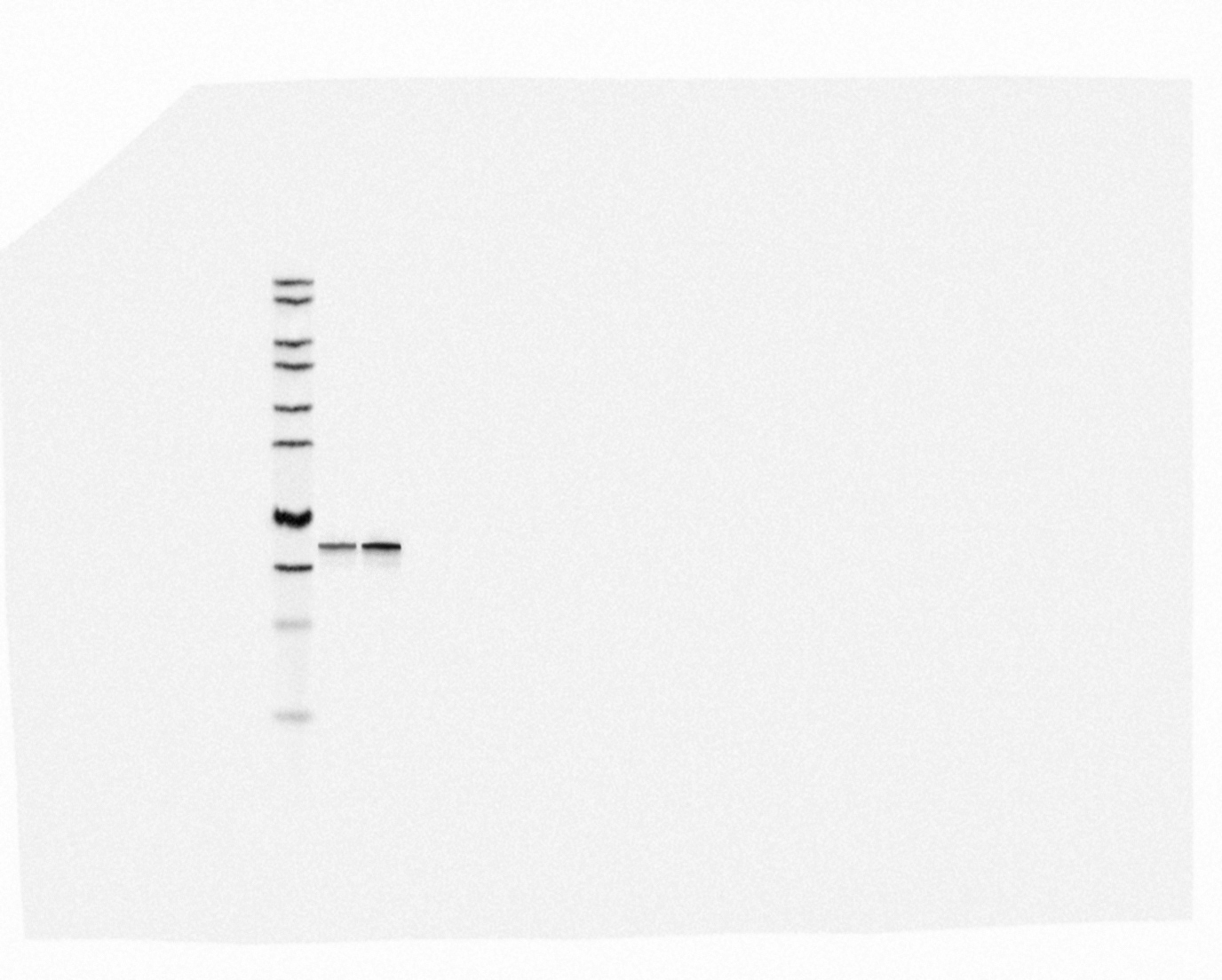

Supplement: Multimedia component 1 [file mmc1.zip › Western Blot and PCR raw data/Screen capture/TIF/CCDC80-2.tif]

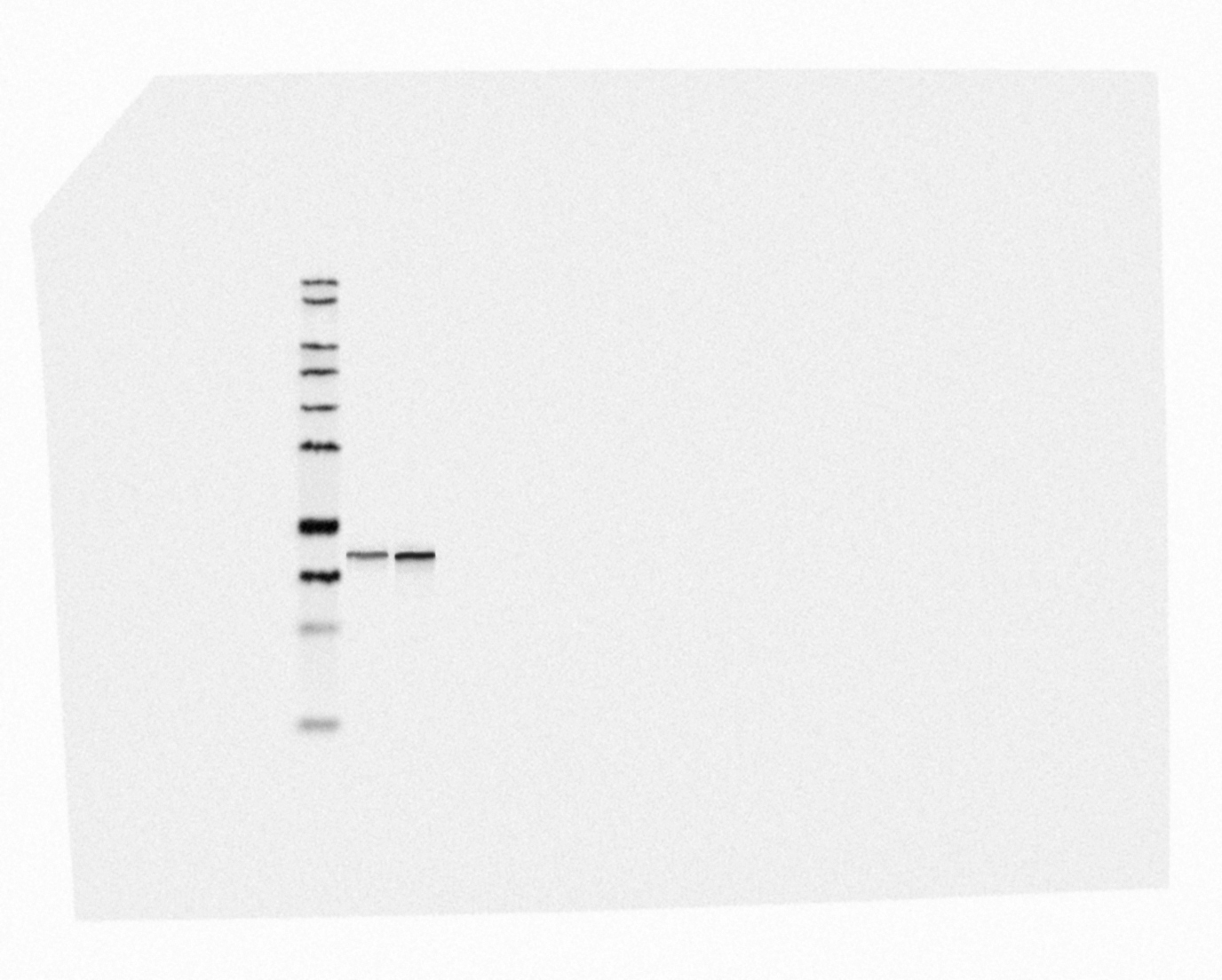

Supplement: Multimedia component 1 [file mmc1.zip › Western Blot and PCR raw data/Screen capture/TIF/CCDC80-3.tif]

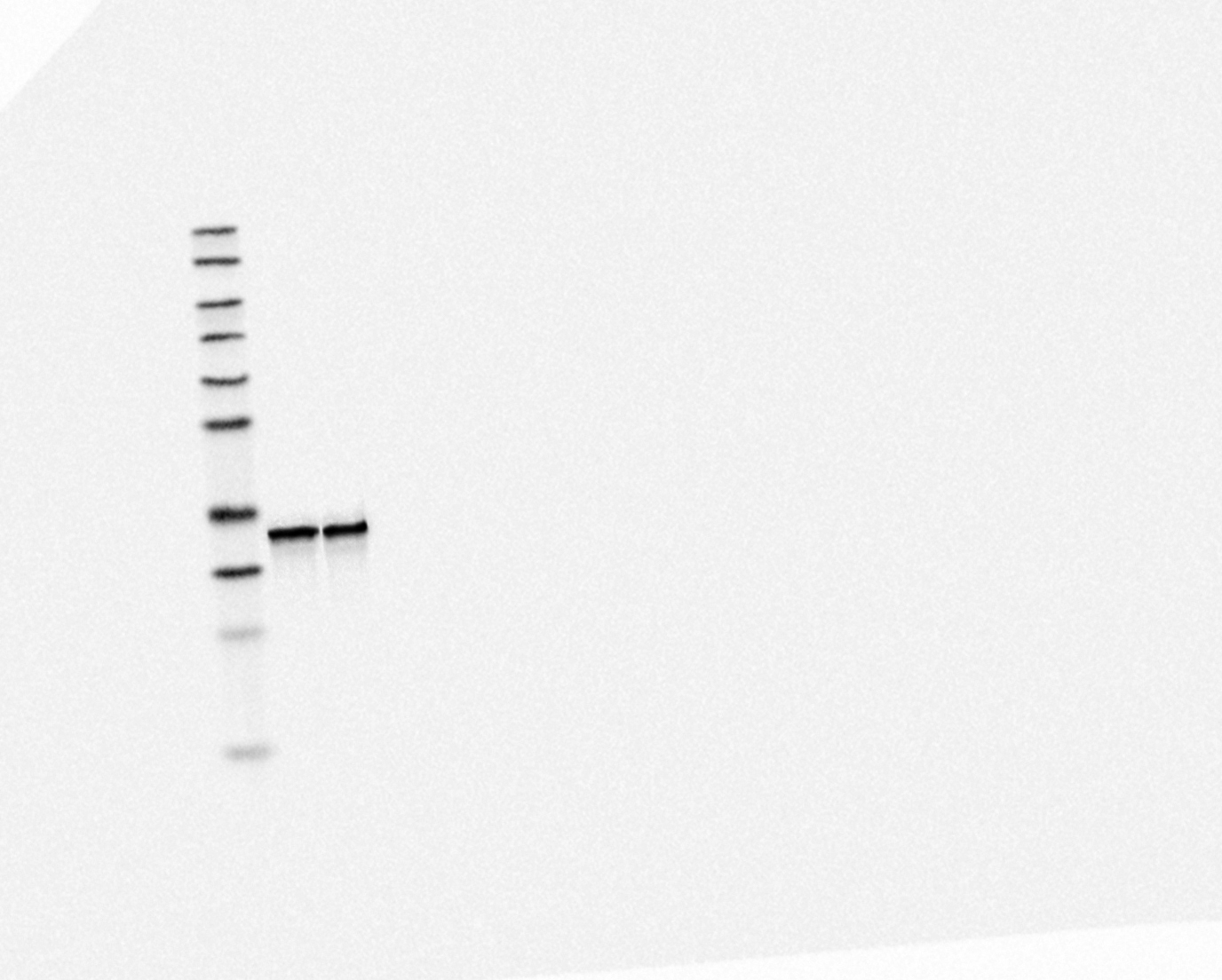

Supplement: Multimedia component 1 [file mmc1.zip › Western Blot and PCR raw data/Screen capture/TIF/GAPDH-1.tif]

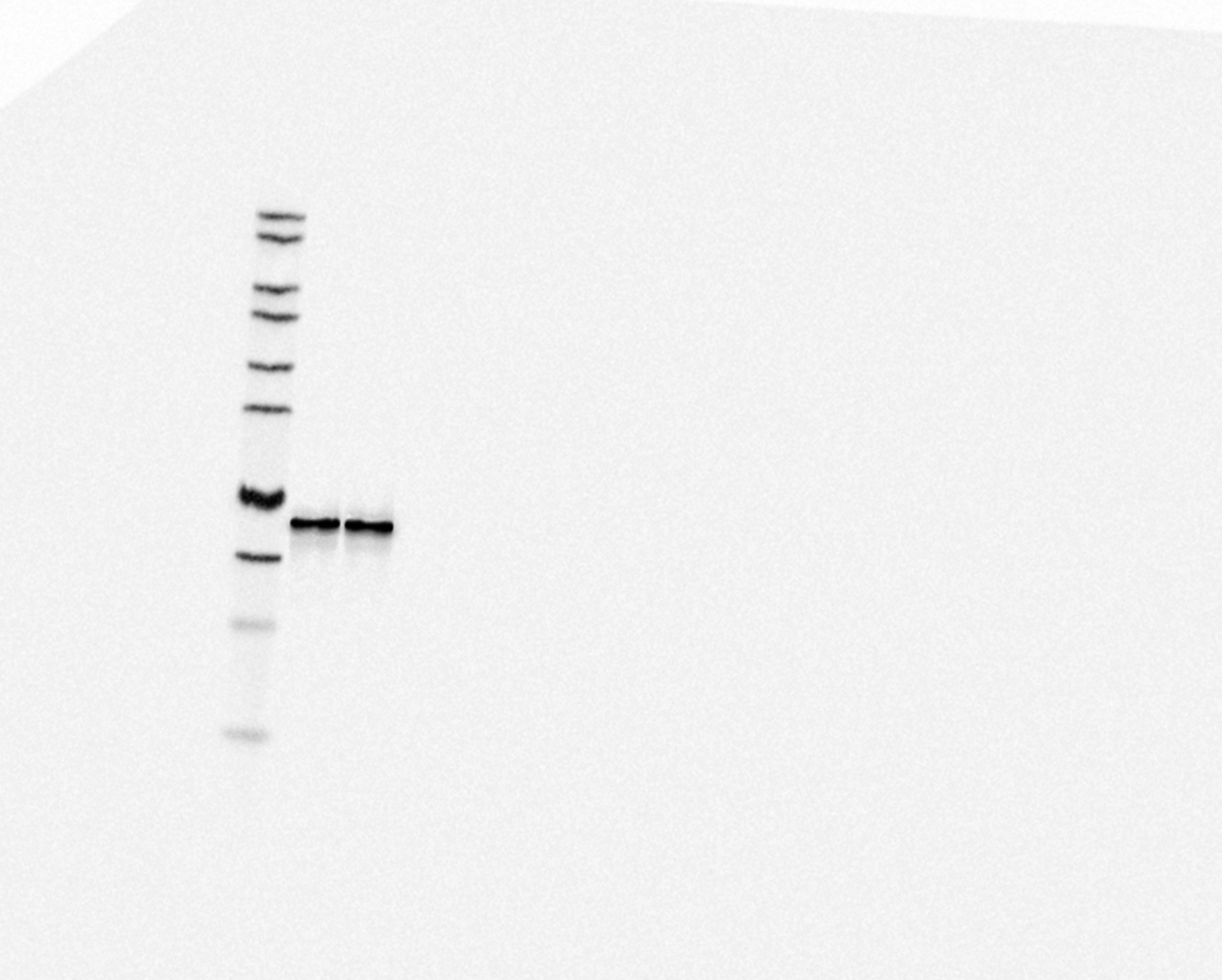

Supplement: Multimedia component 1 [file mmc1.zip › Western Blot and PCR raw data/Screen capture/TIF/GAPDH-2.tif]

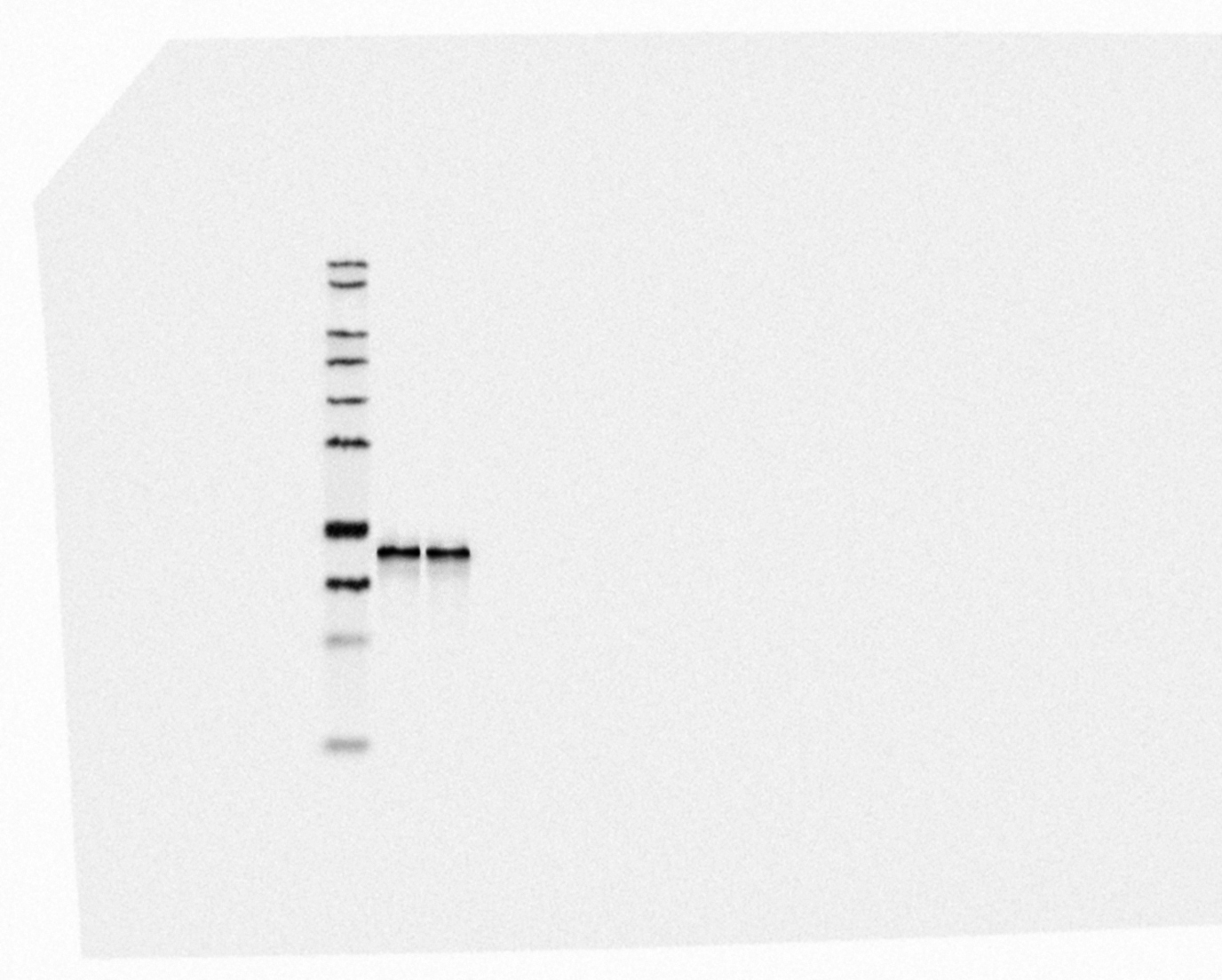

Supplement: Multimedia component 1 [file mmc1.zip › Western Blot and PCR raw data/Screen capture/TIF/GAPDH-3.tif]

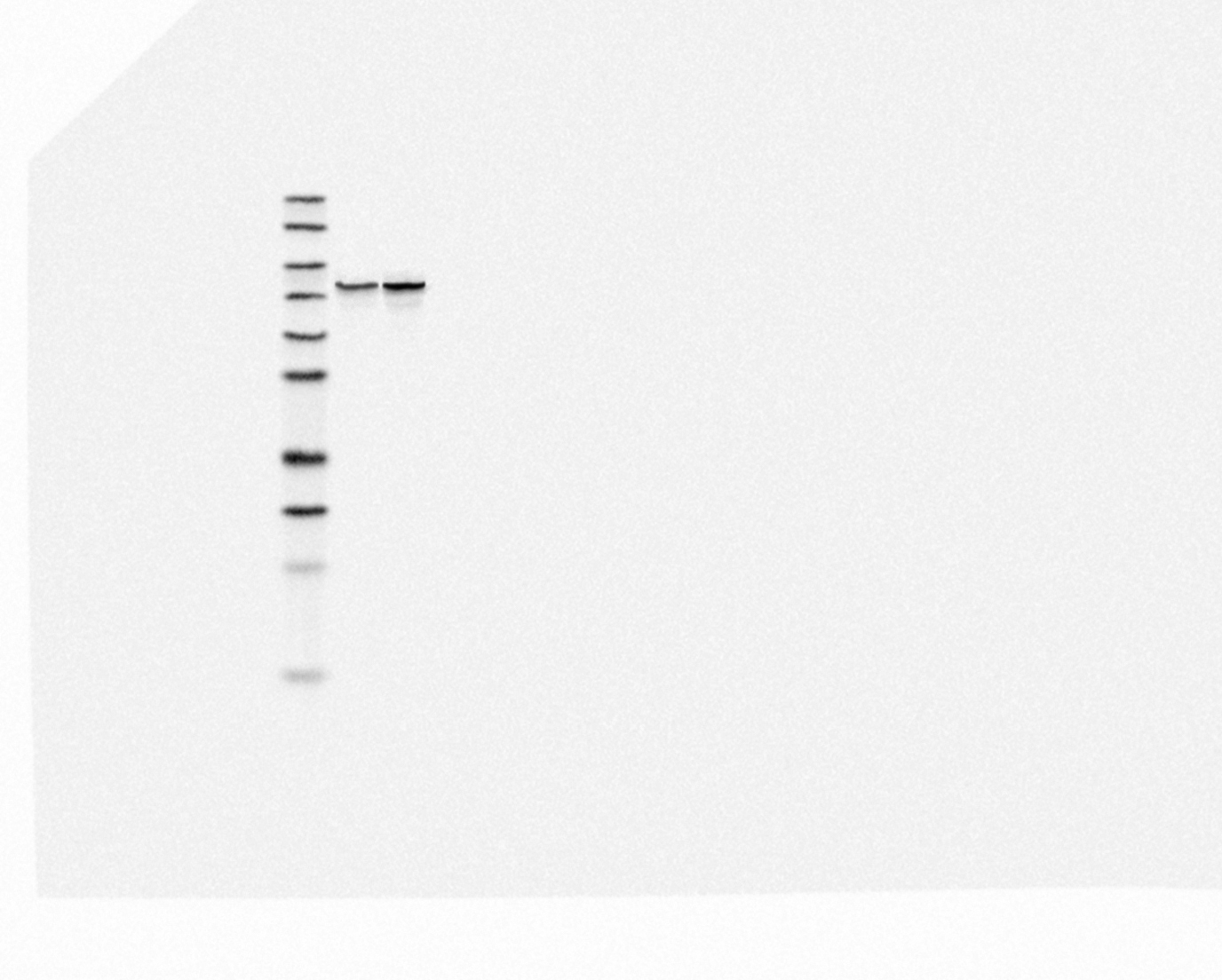

Supplement: Multimedia component 1 [file mmc1.zip › Western Blot and PCR raw data/Screen capture/TIF/SMURF2-1.tif]

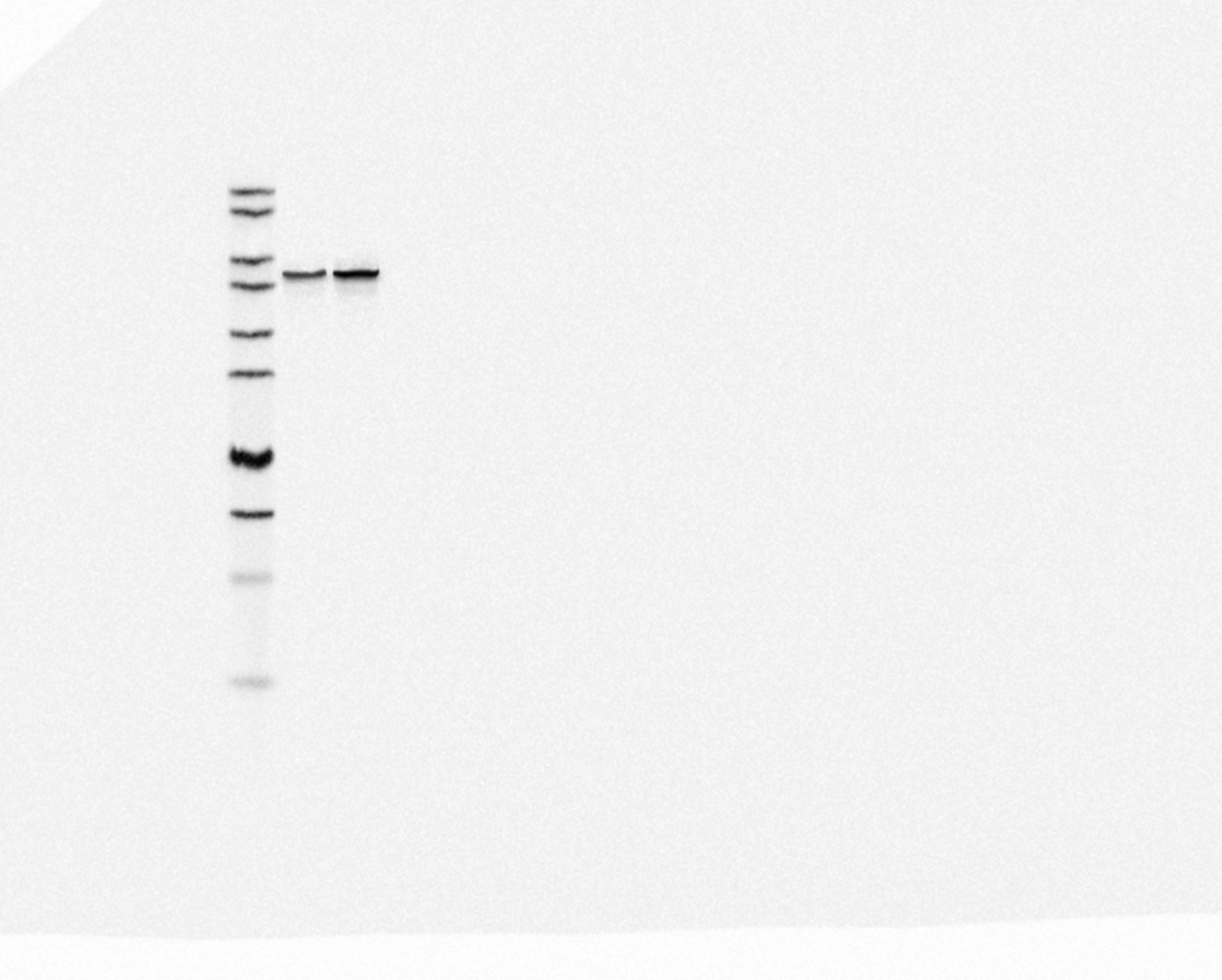

Supplement: Multimedia component 1 [file mmc1.zip › Western Blot and PCR raw data/Screen capture/TIF/SMURF2-2.tif]

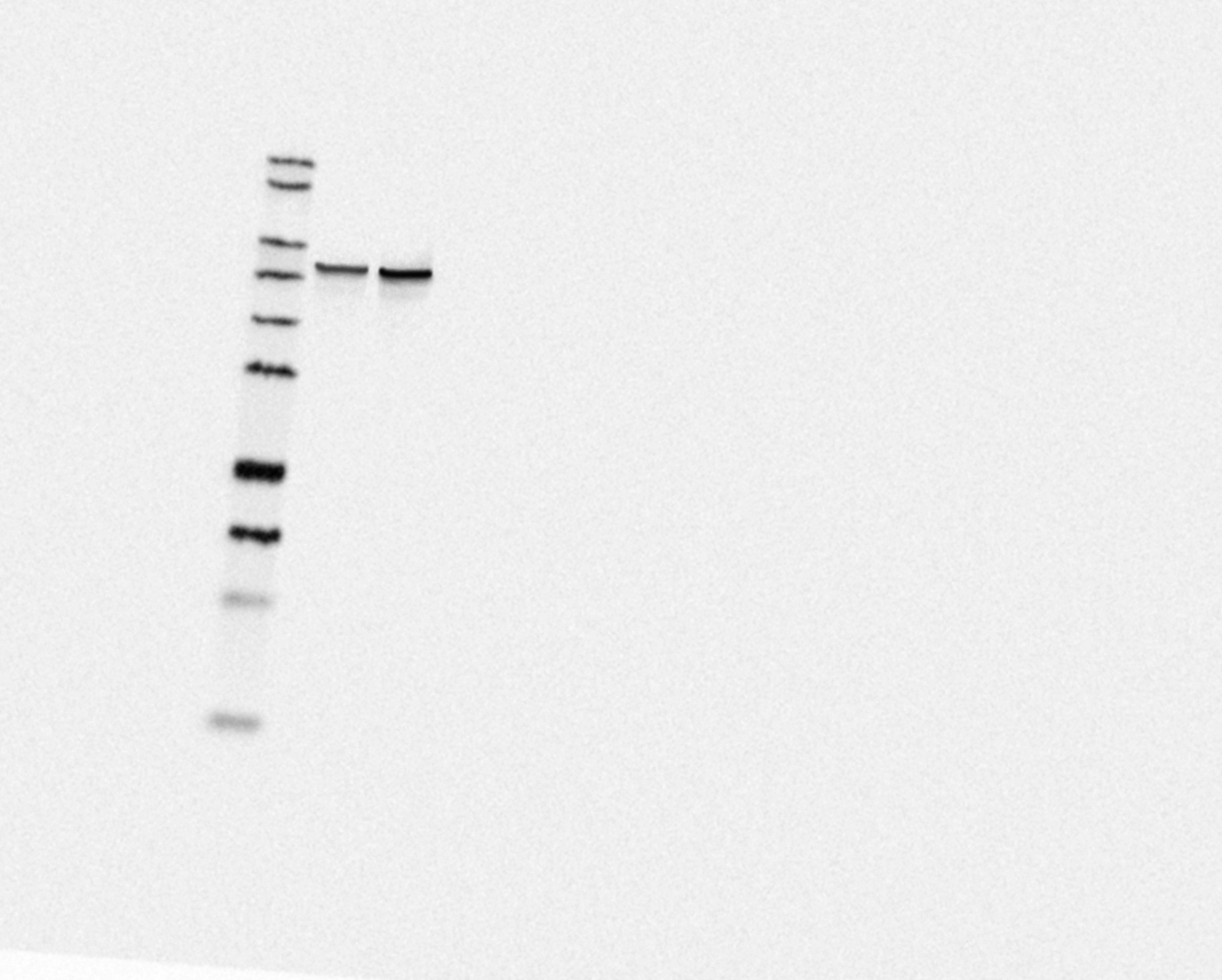

Supplement: Multimedia component 1 [file mmc1.zip › Western Blot and PCR raw data/Screen capture/TIF/SMURF2-3.tif]

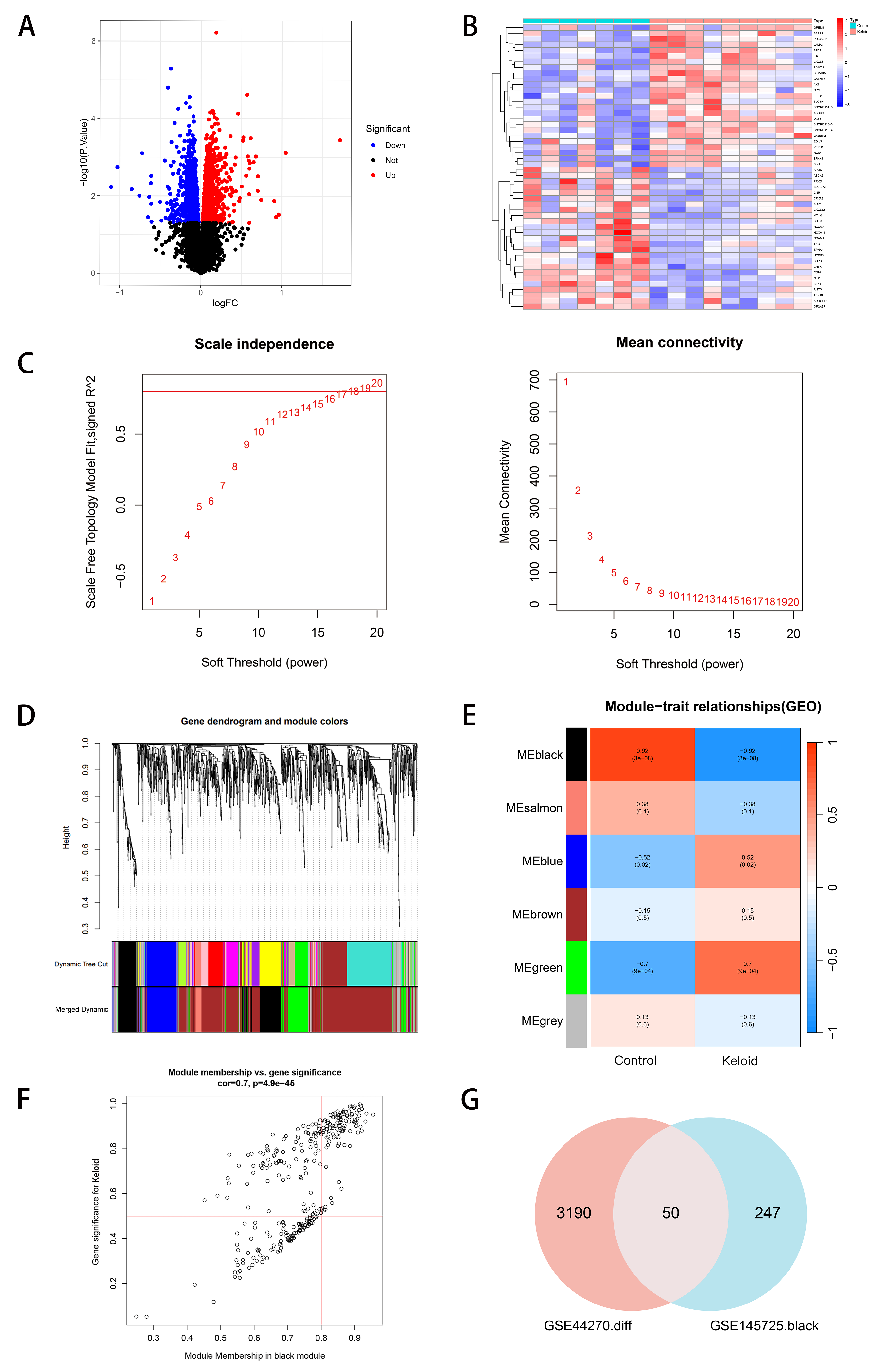

Supplement: Multimedia component 2 [file mmc2.zip › A set of pictures/Figure1.tif]

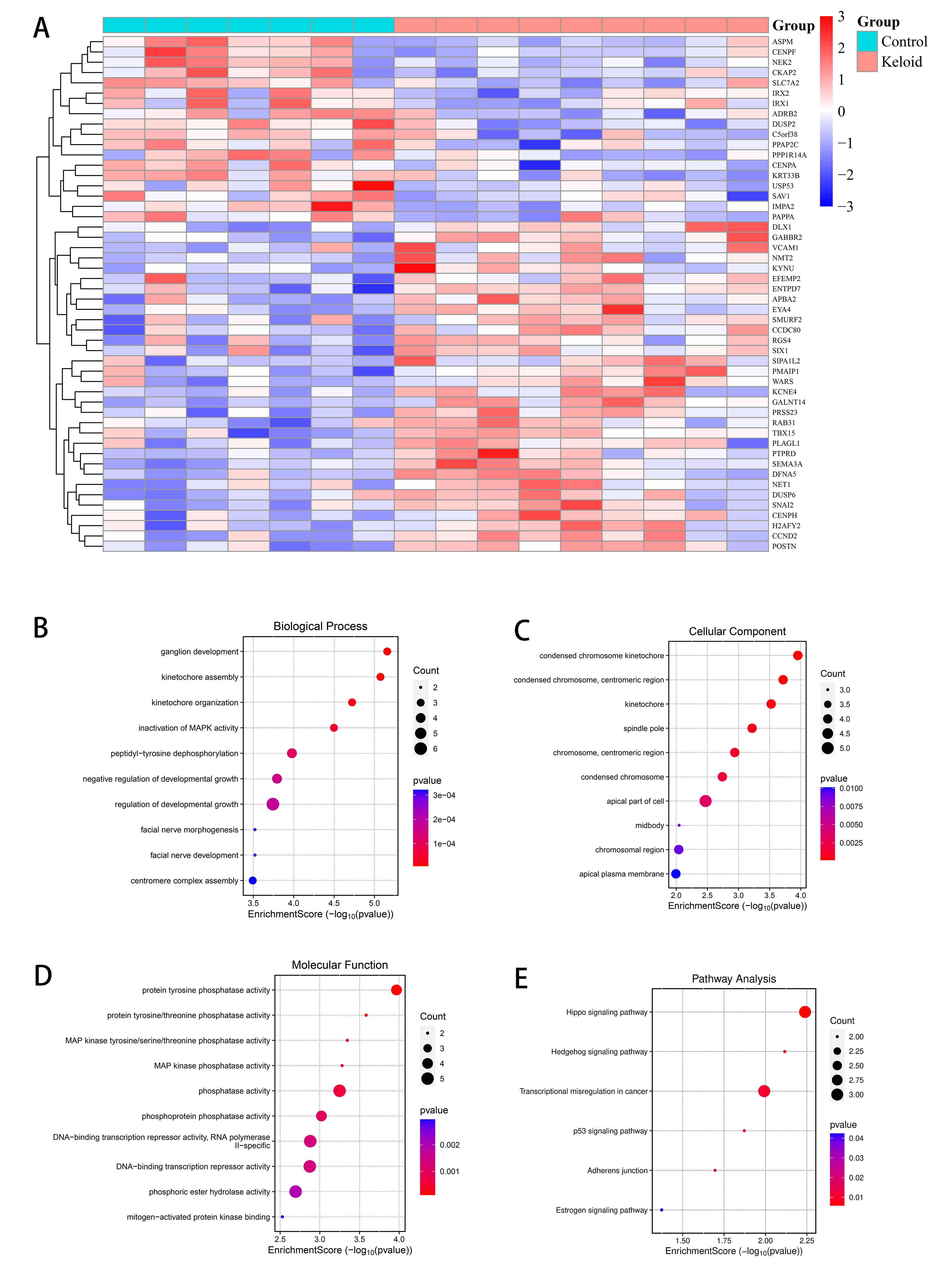

Supplement: Multimedia component 2 [file mmc2.zip › A set of pictures/Figure2.tif]

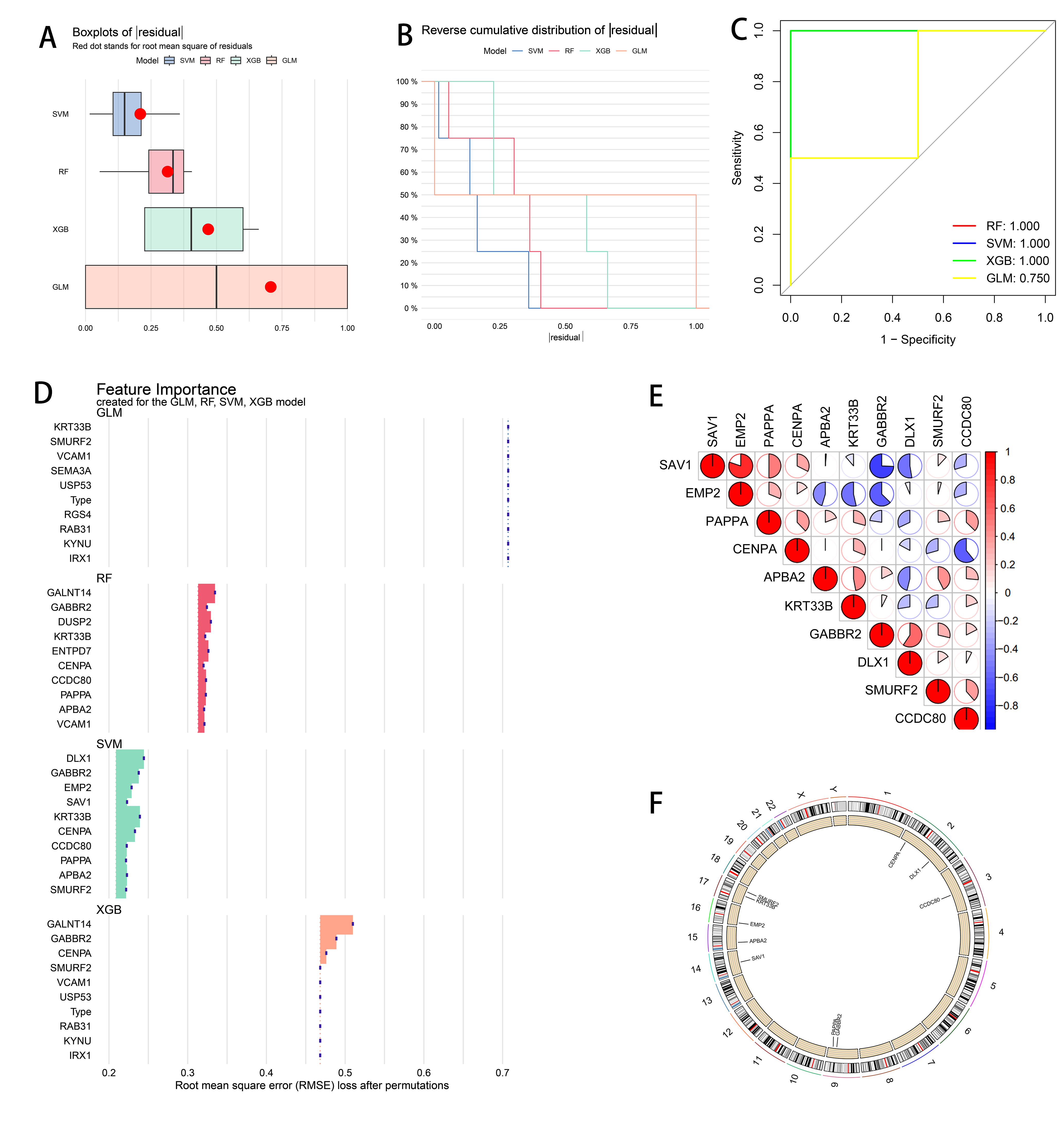

Supplement: Multimedia component 2 [file mmc2.zip › A set of pictures/Figure3.tif]

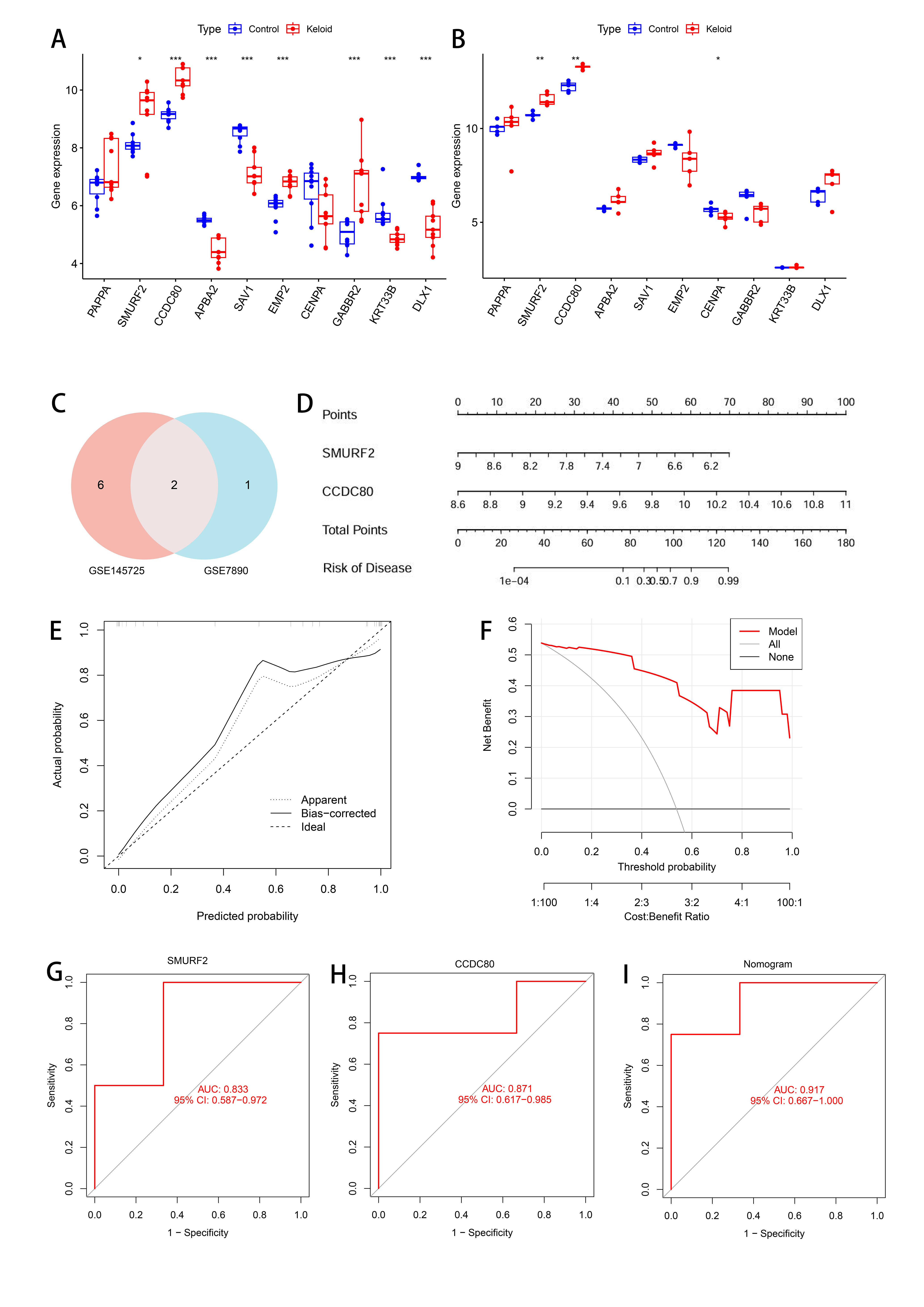

Supplement: Multimedia component 2 [file mmc2.zip › A set of pictures/Figure4.tif]

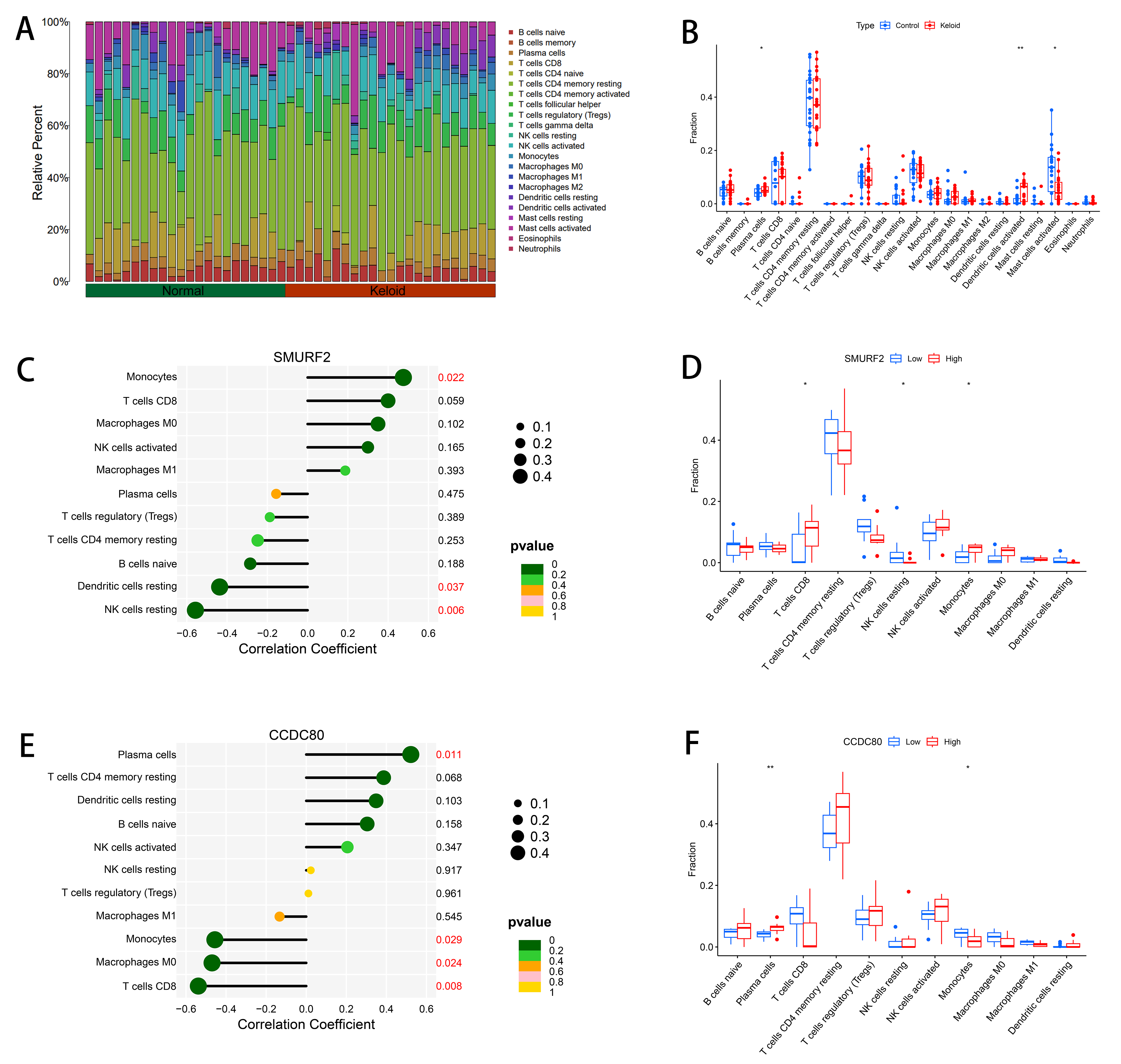

Supplement: Multimedia component 2 [file mmc2.zip › A set of pictures/Figure5.tif]

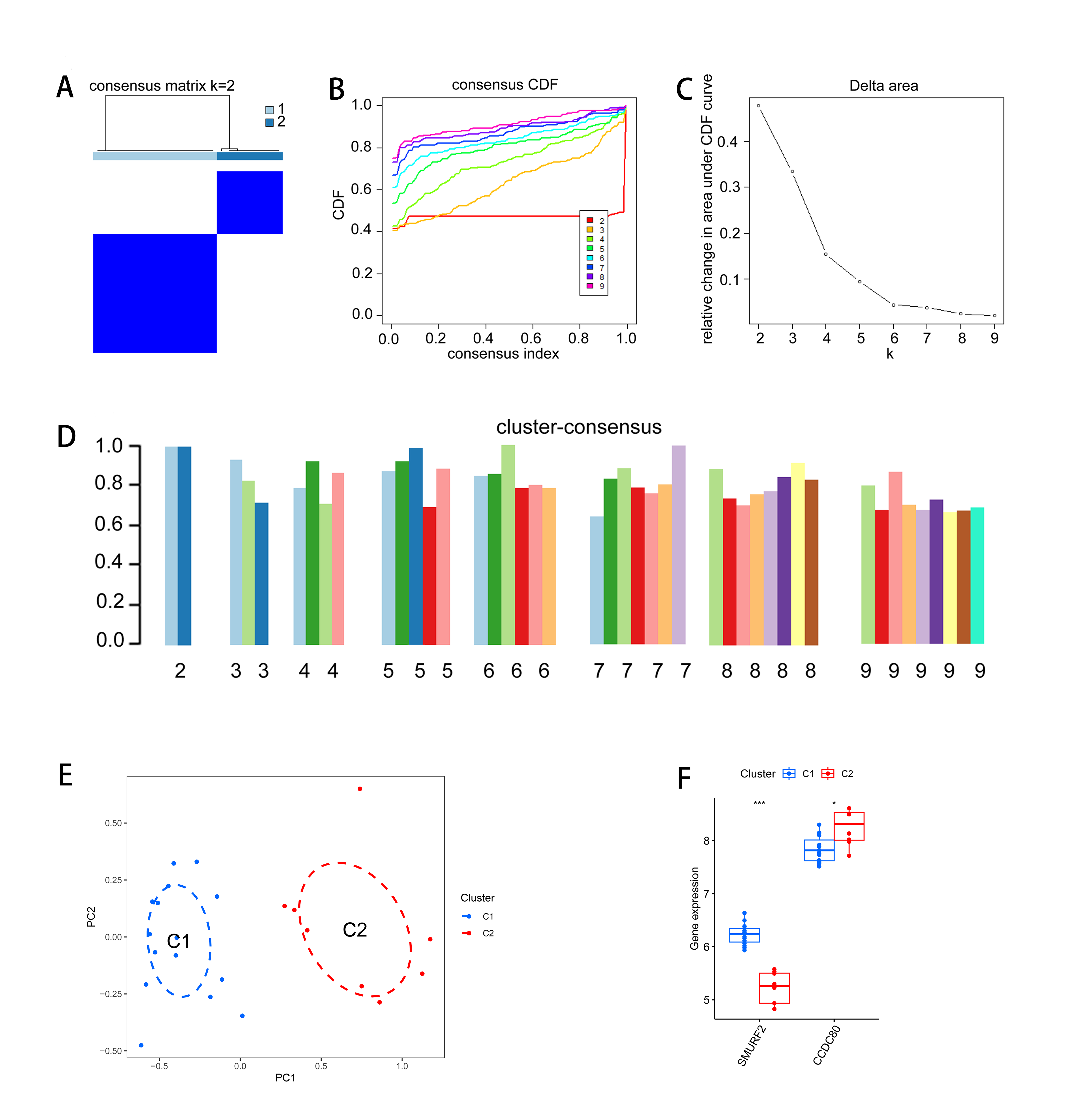

Supplement: Multimedia component 2 [file mmc2.zip › A set of pictures/Figure6.tif]

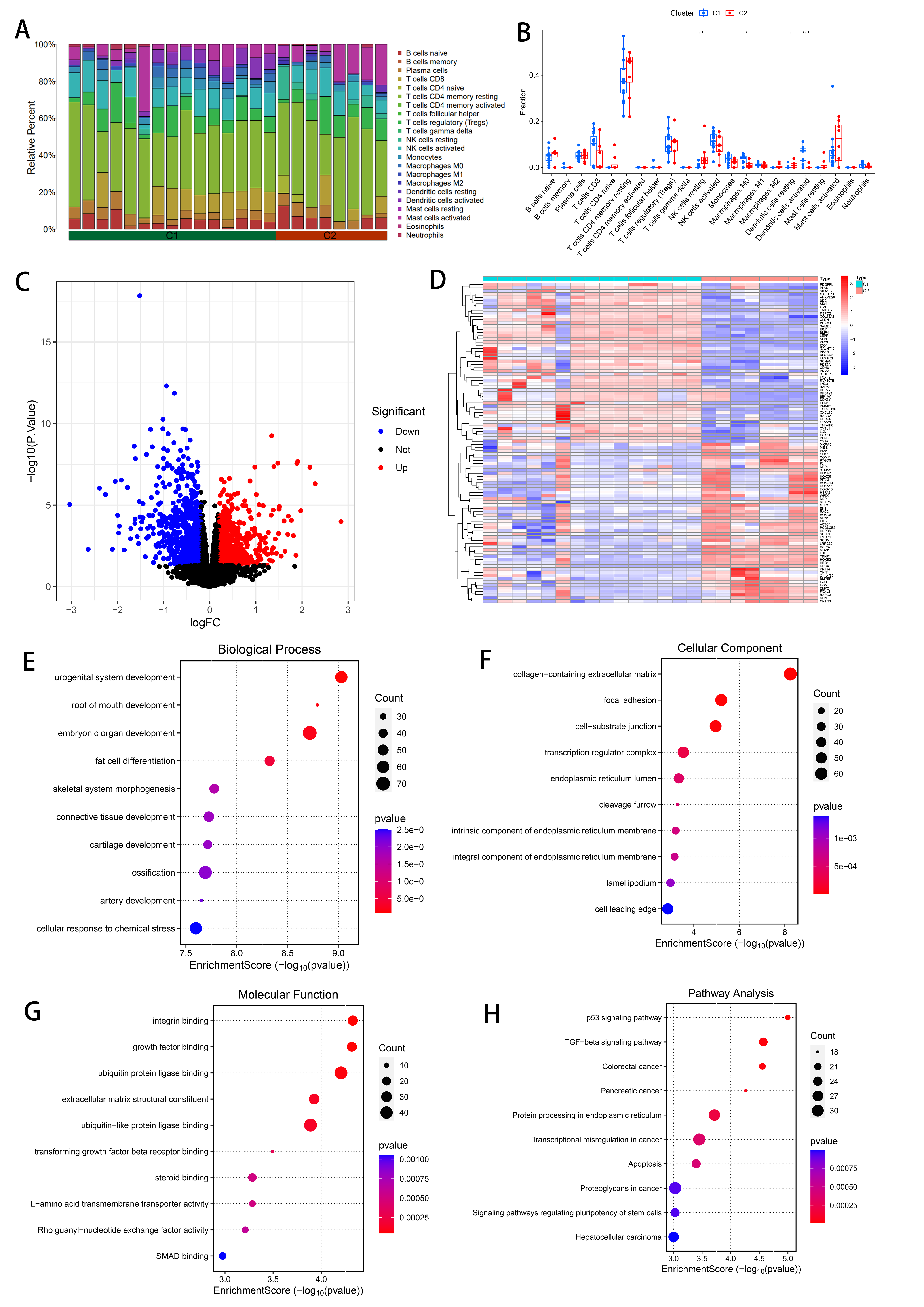

Supplement: Multimedia component 2 [file mmc2.zip › A set of pictures/Figure7.tif]

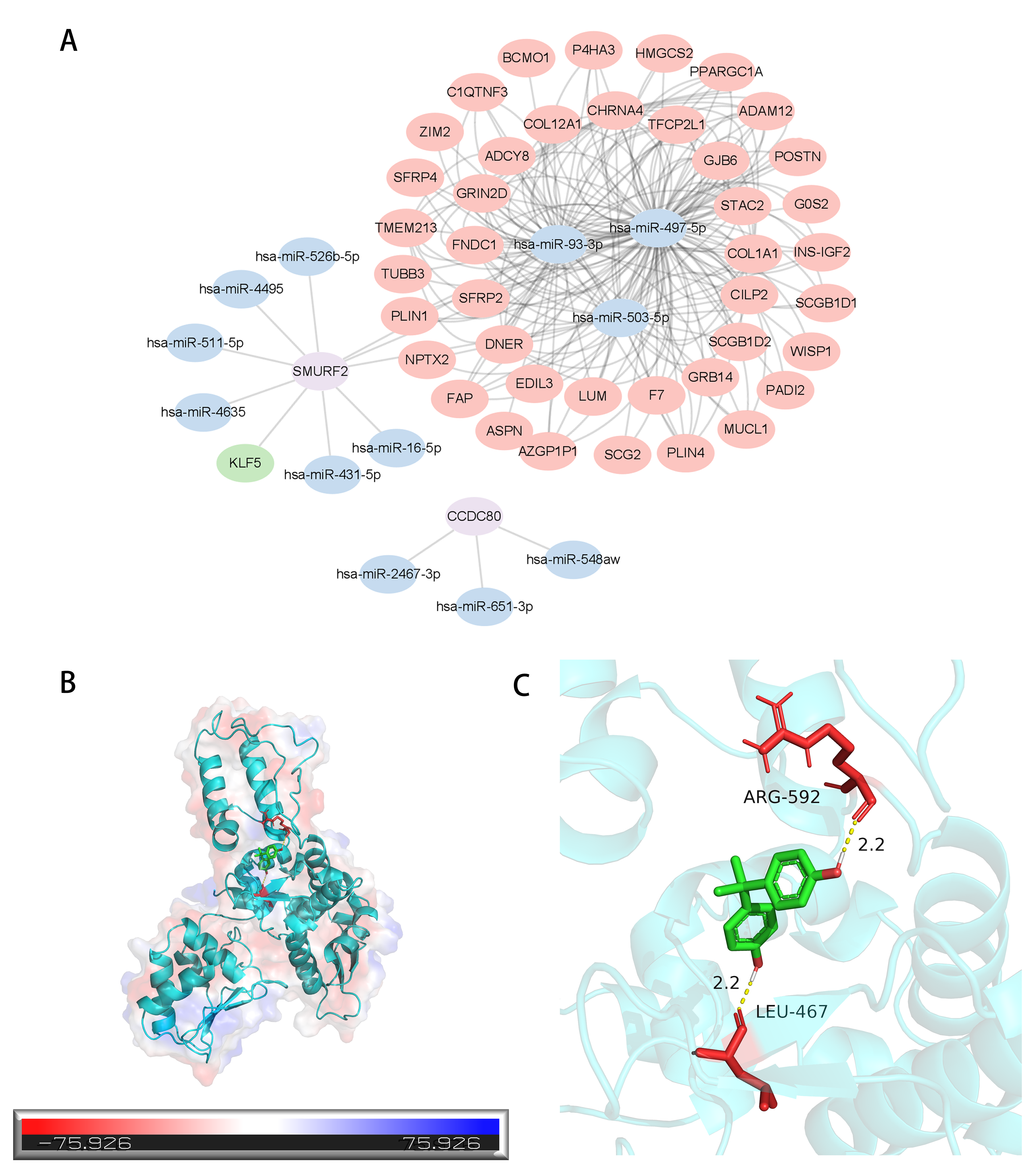

Supplement: Multimedia component 2 [file mmc2.zip › A set of pictures/Figure8.tif]

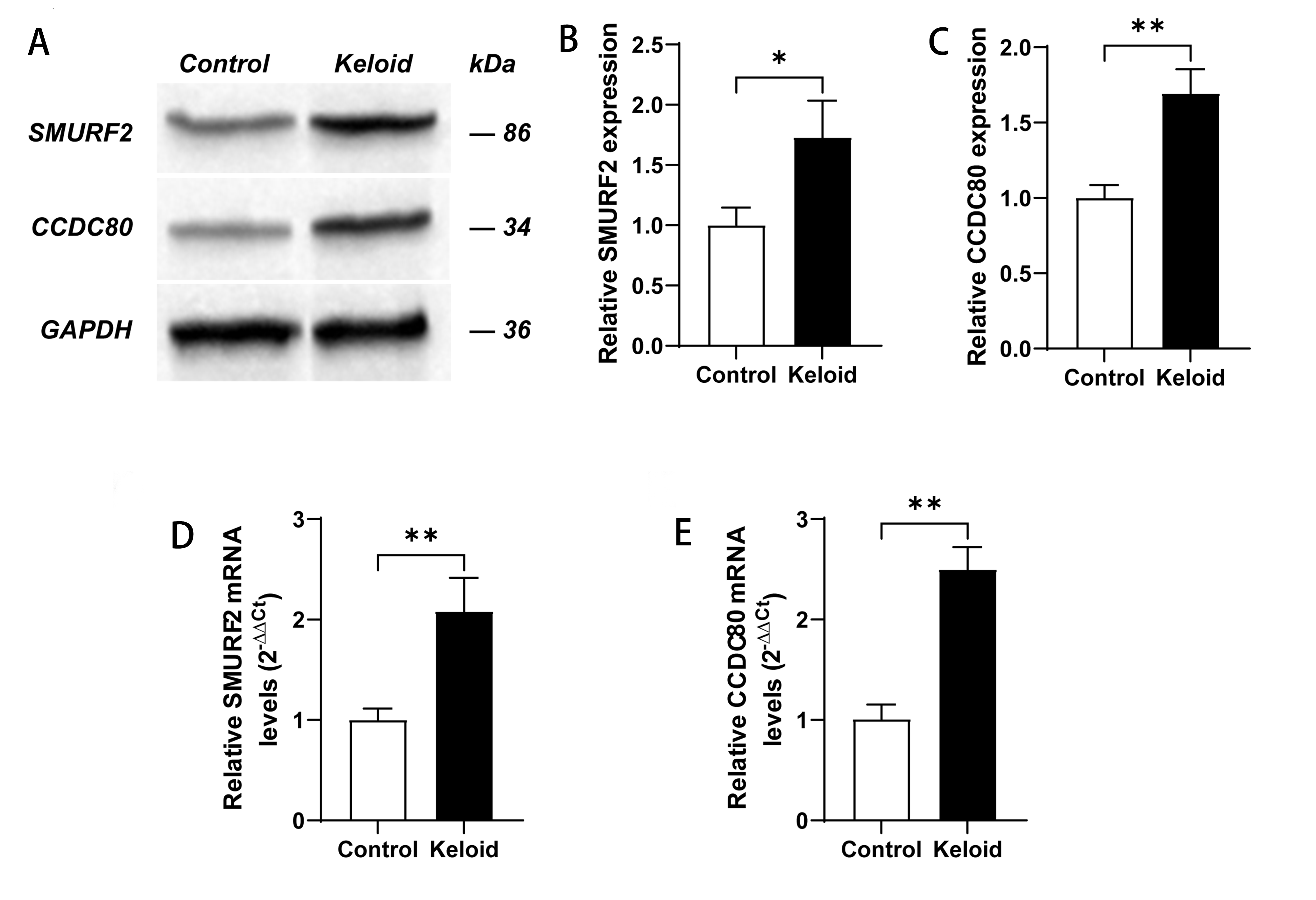

Supplement: Multimedia component 2 [file mmc2.zip › A set of pictures/Figure9.tif]

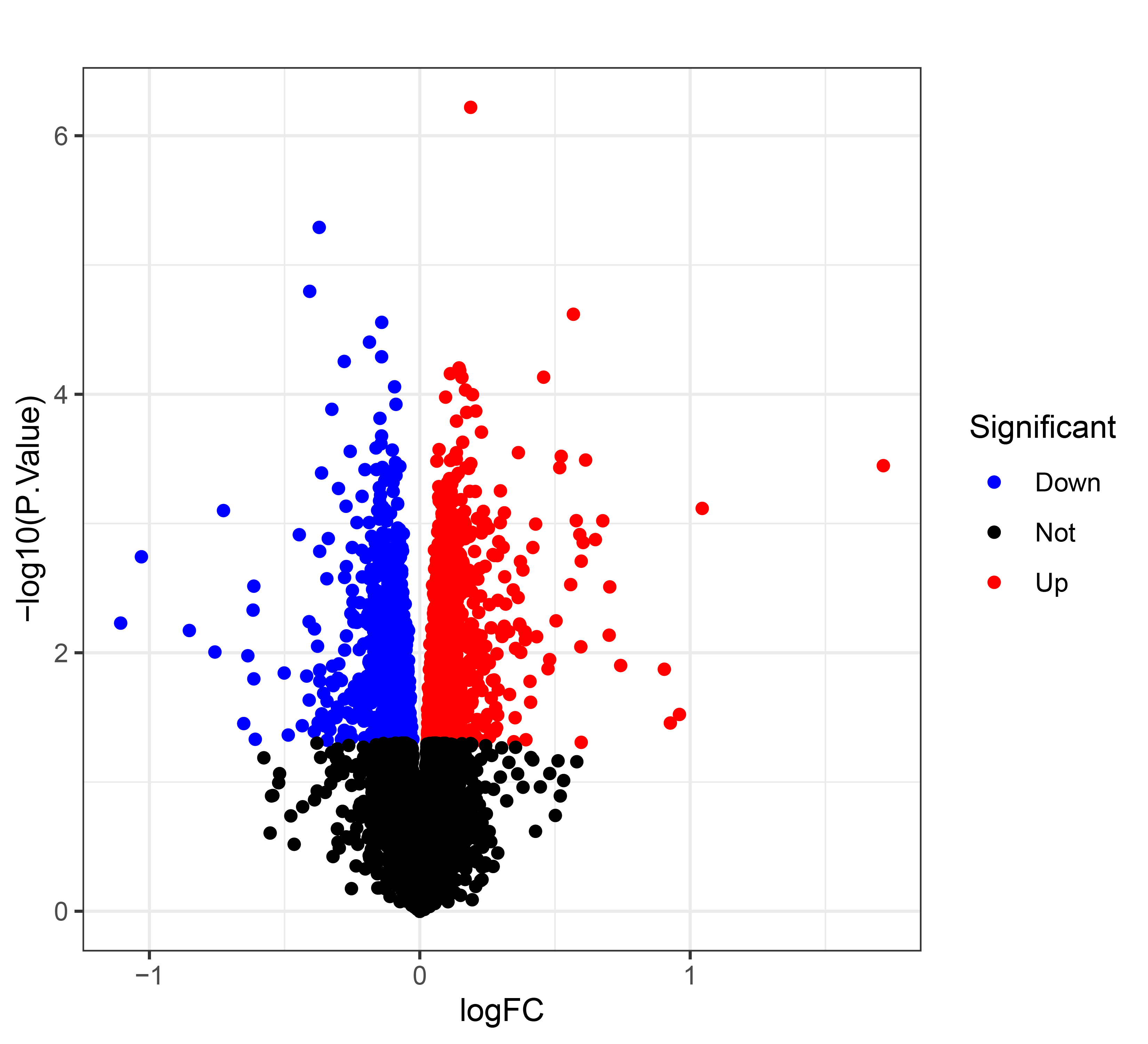

Supplement: Multimedia component 3 [file mmc3.zip › Single image/1A.tif]

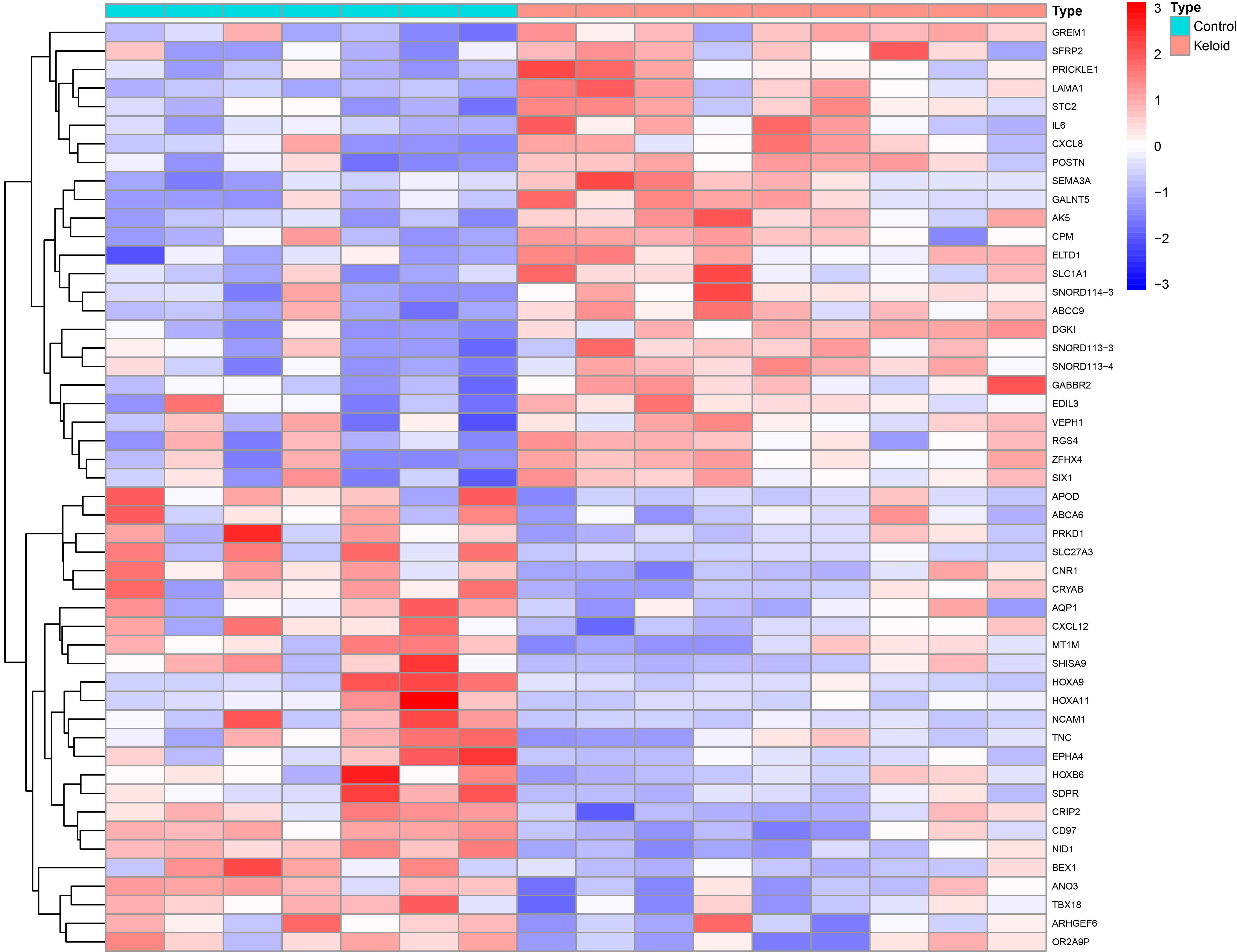

Supplement: Multimedia component 3 [file mmc3.zip › Single image/1B.tif]

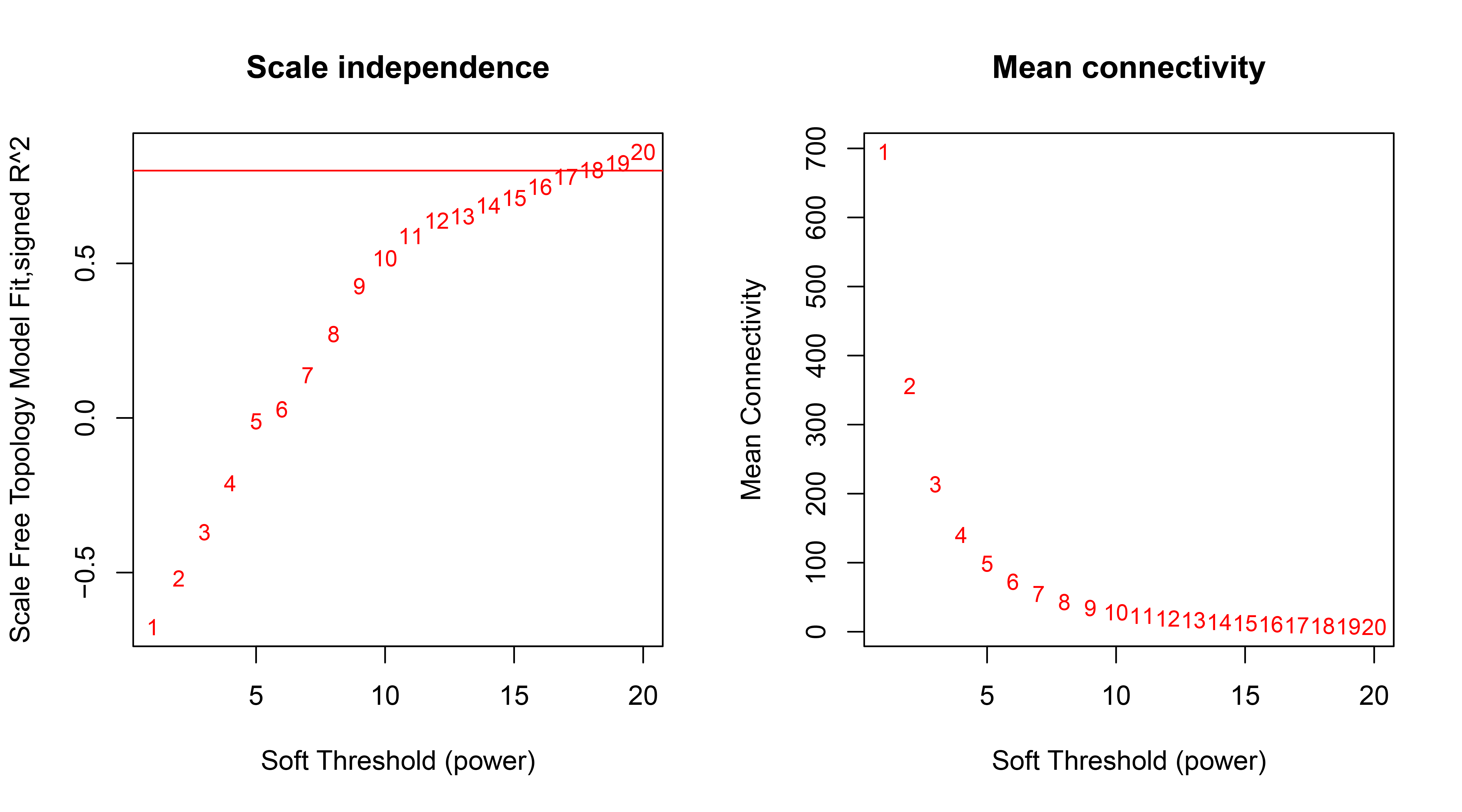

Supplement: Multimedia component 3 [file mmc3.zip › Single image/1C.tif]

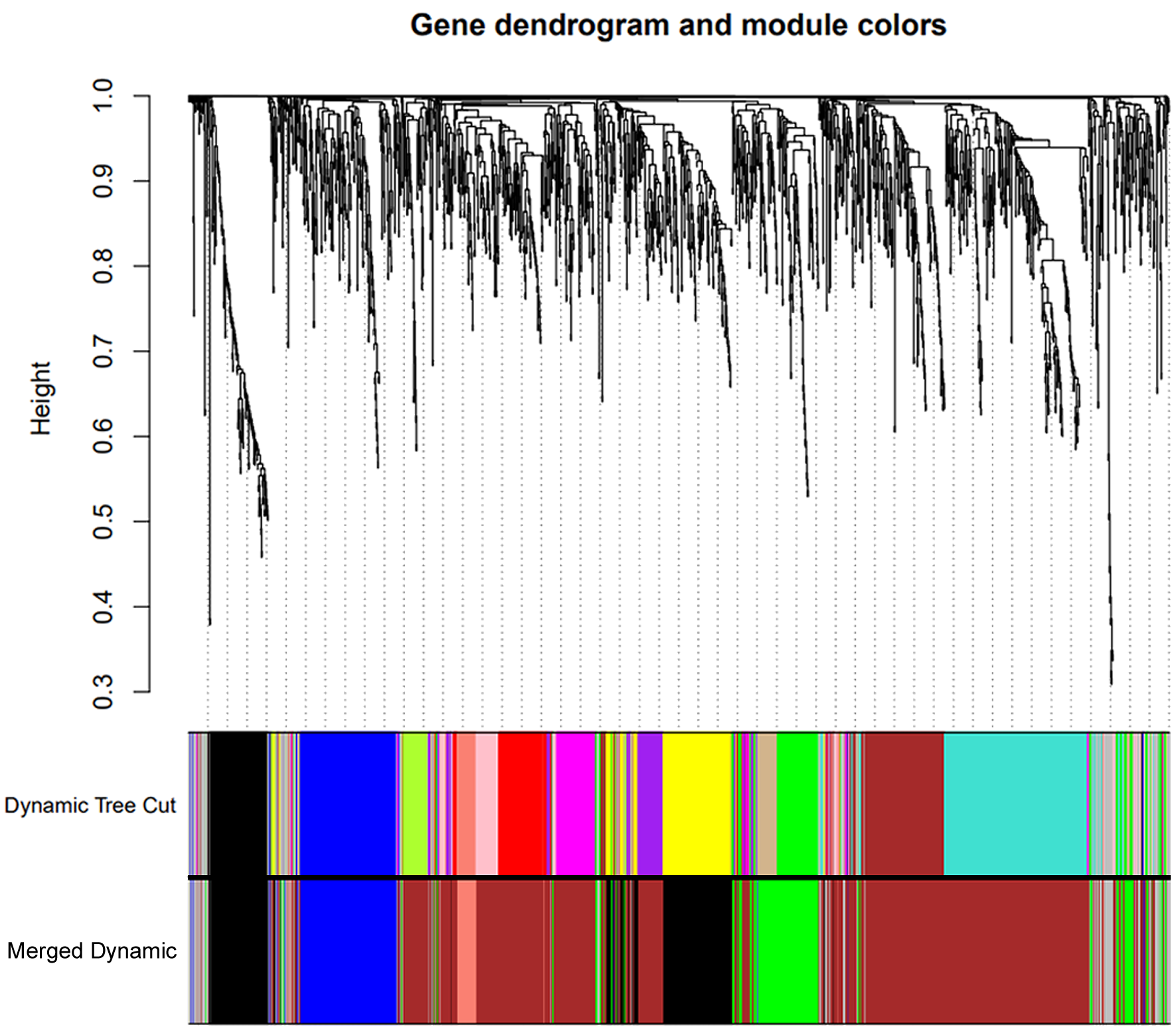

Supplement: Multimedia component 3 [file mmc3.zip › Single image/1D.tif]

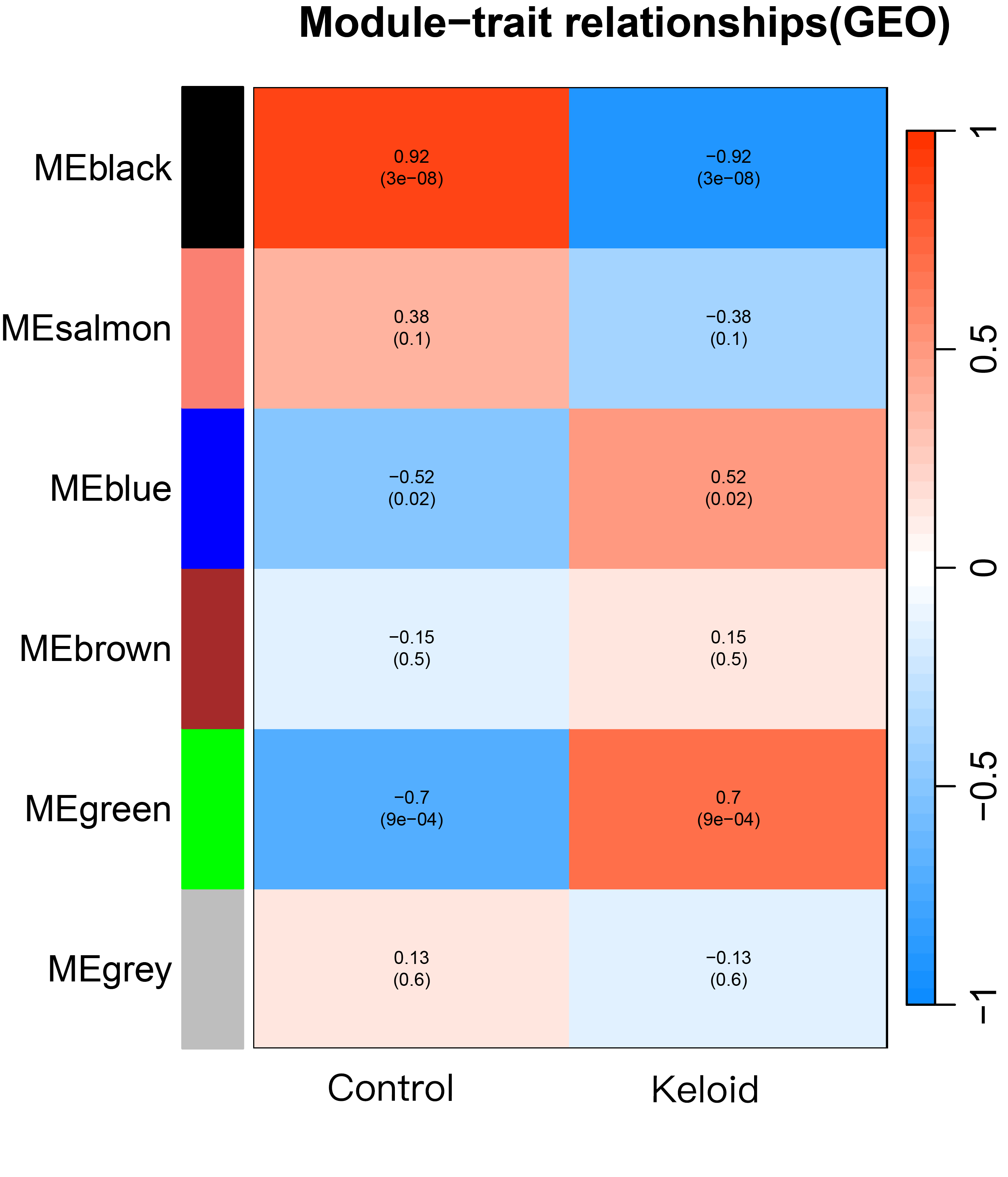

Supplement: Multimedia component 3 [file mmc3.zip › Single image/1E.tif]

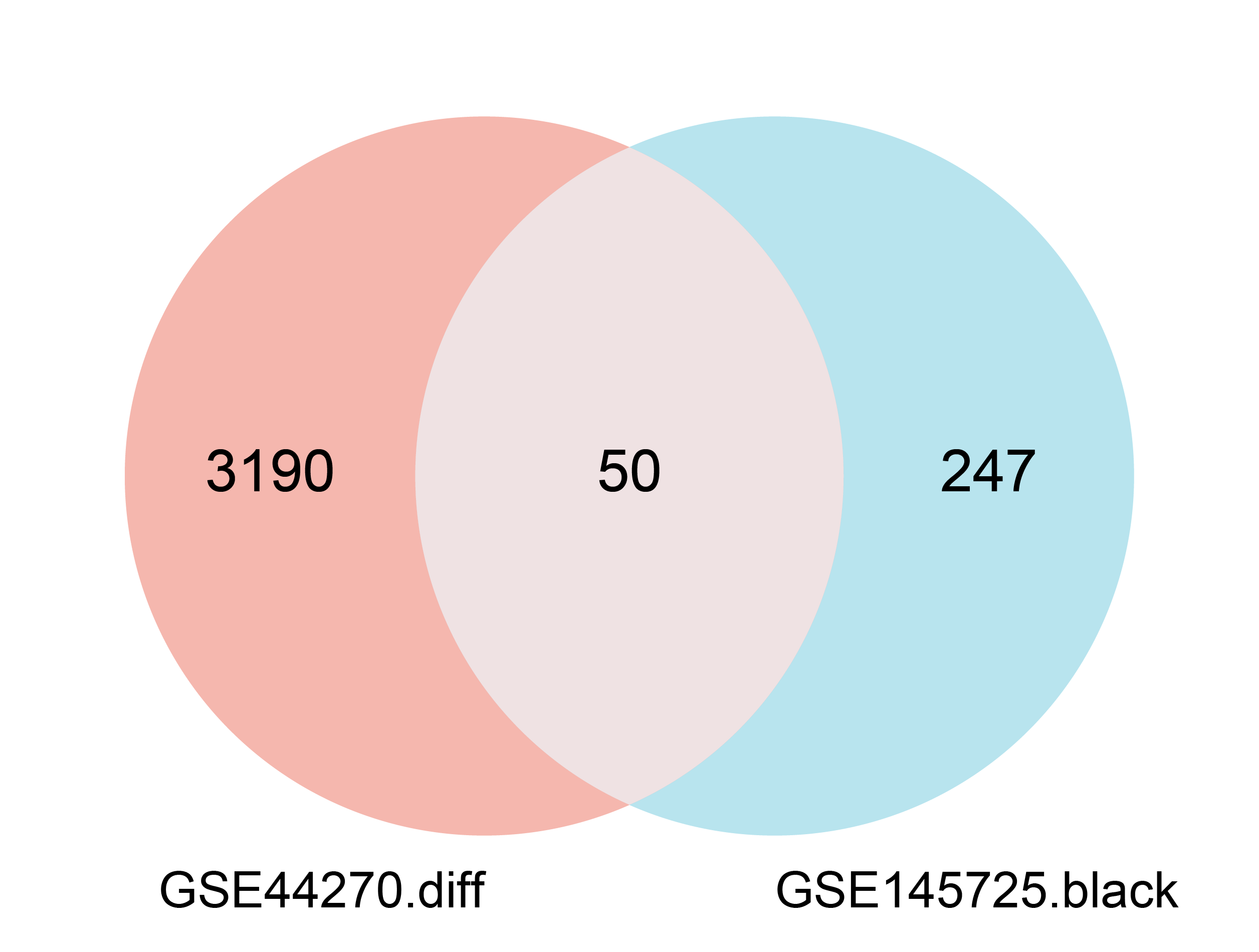

Supplement: Multimedia component 3 [file mmc3.zip › Single image/1G.tif]

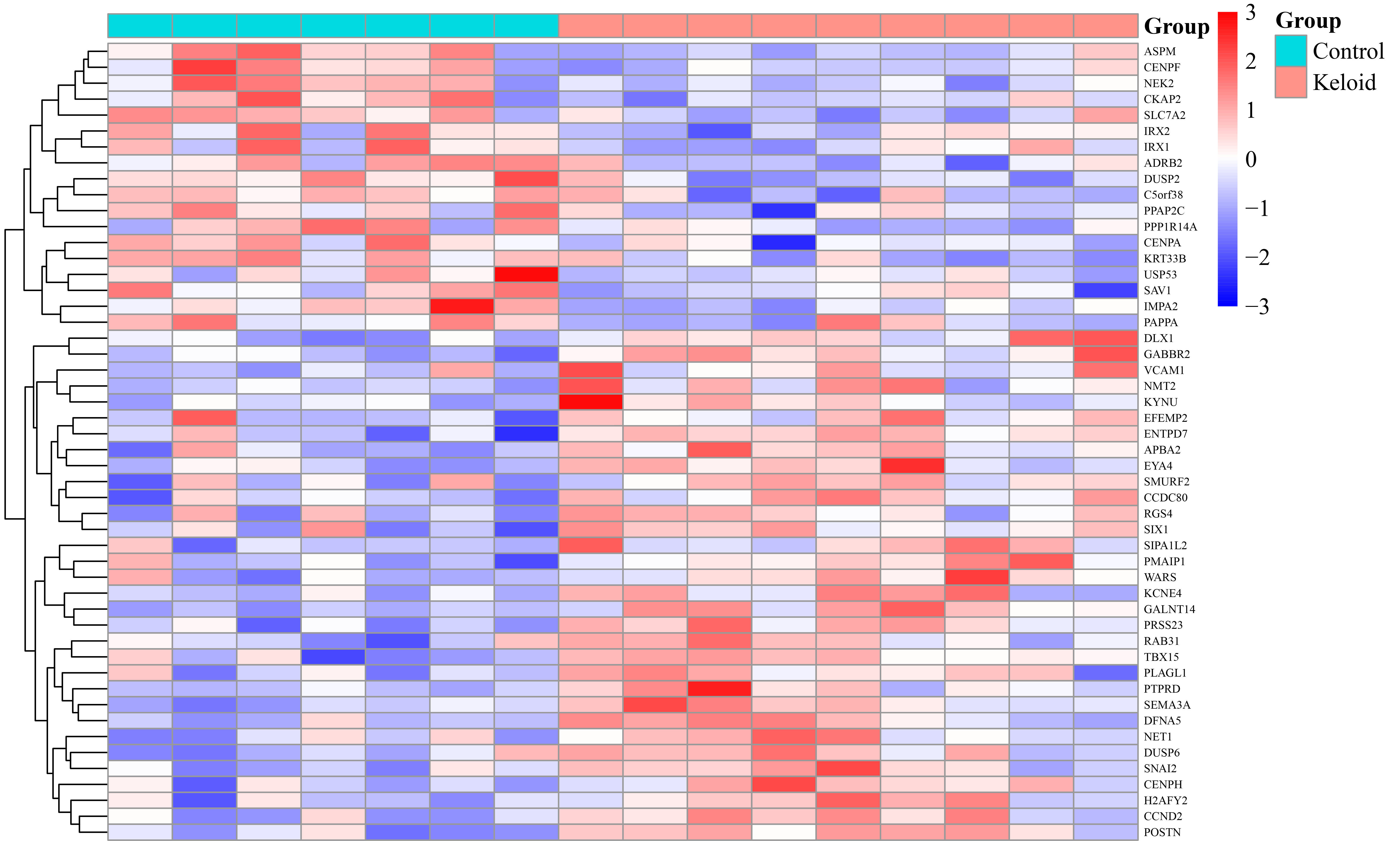

Supplement: Multimedia component 3 [file mmc3.zip › Single image/2A.tif]

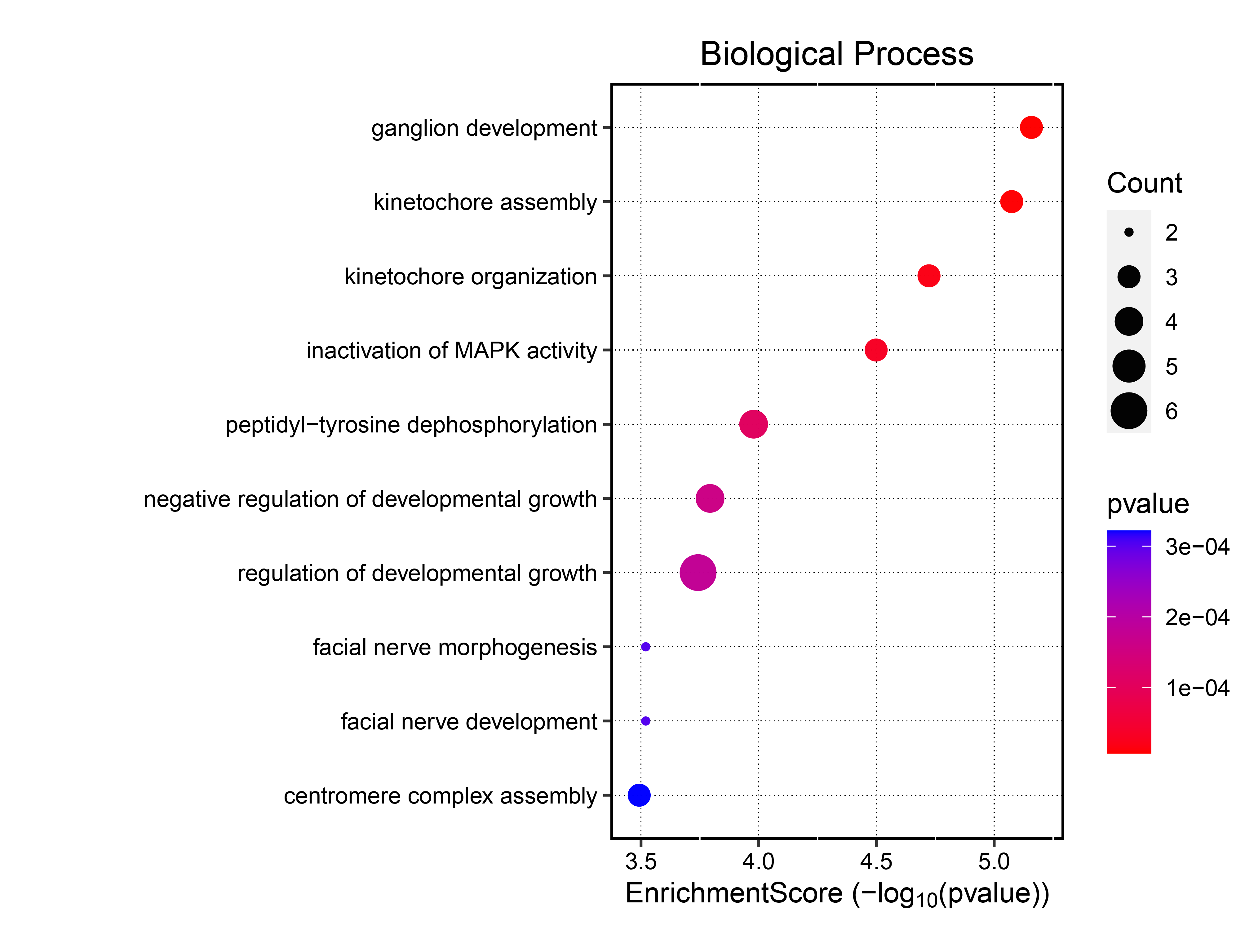

Supplement: Multimedia component 3 [file mmc3.zip › Single image/2B.tif]

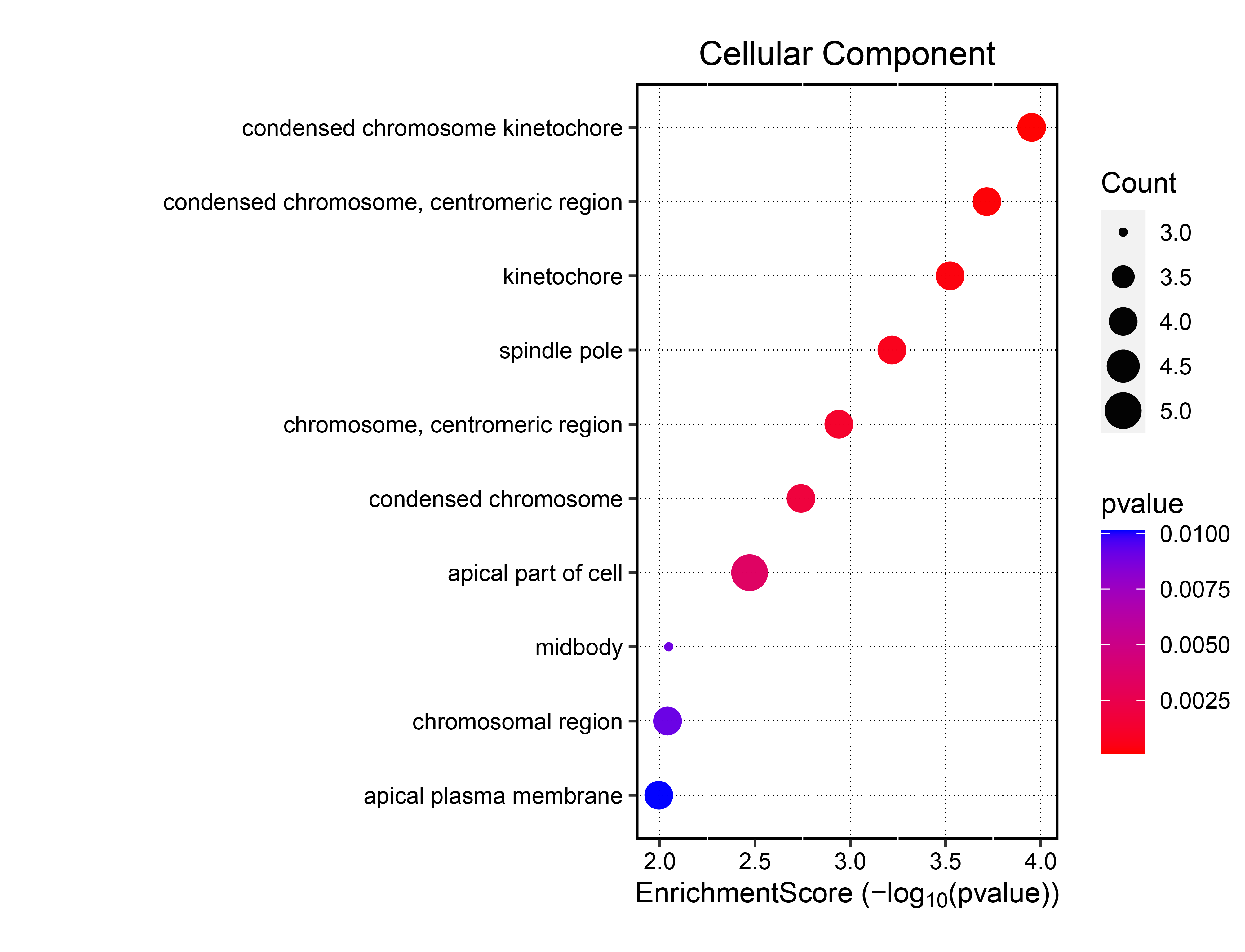

Supplement: Multimedia component 3 [file mmc3.zip › Single image/2C.tif]

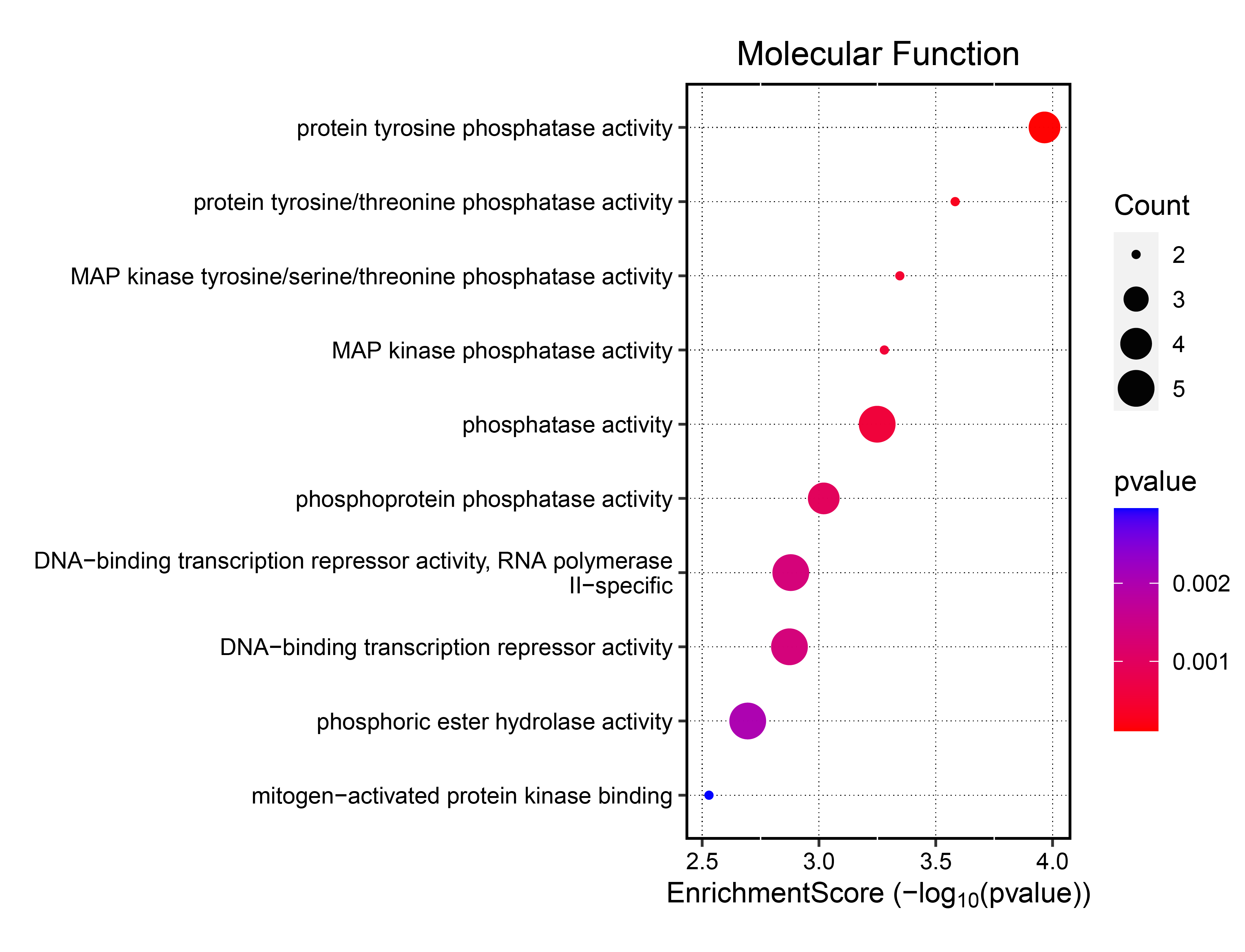

Supplement: Multimedia component 3 [file mmc3.zip › Single image/2D.tif]

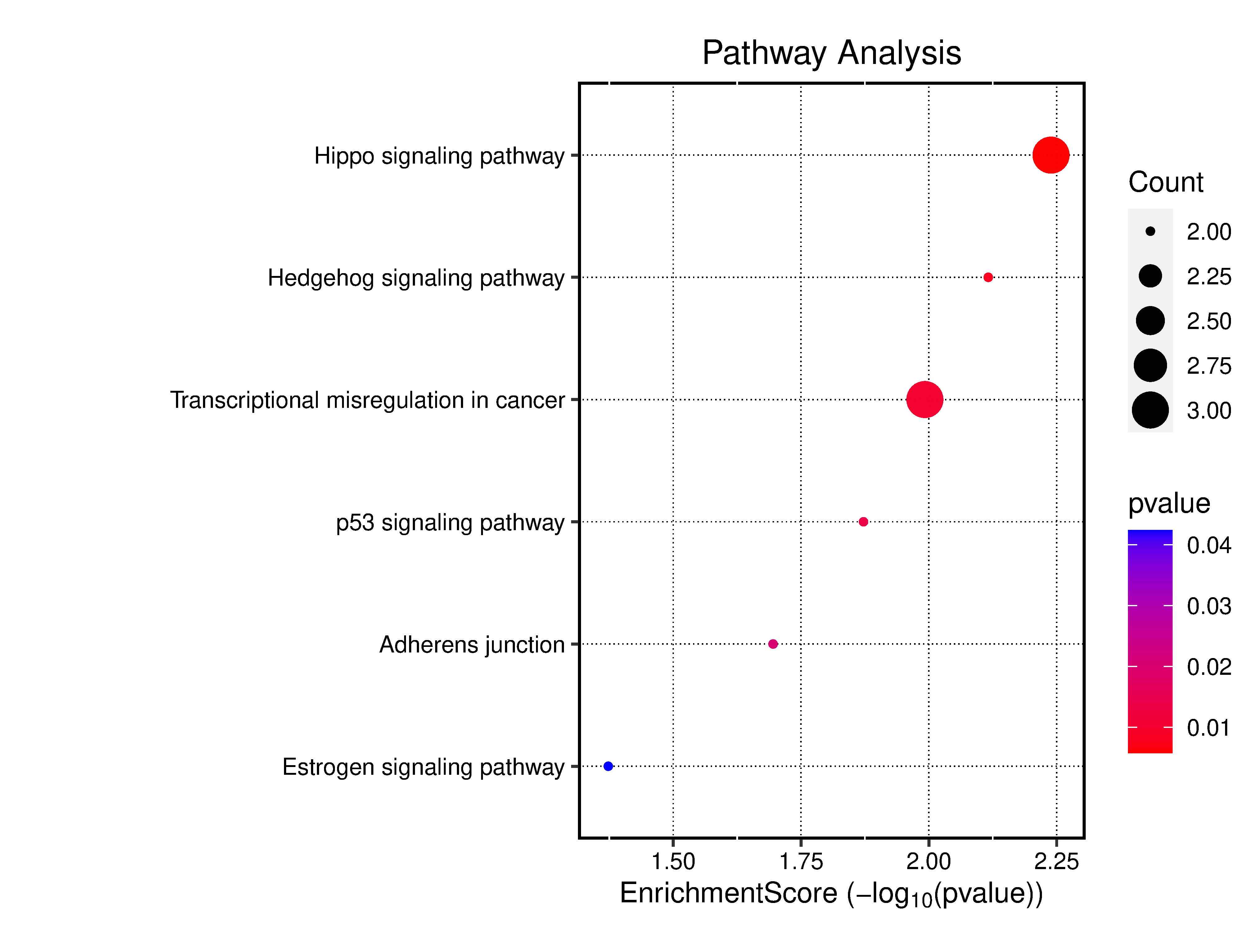

Supplement: Multimedia component 3 [file mmc3.zip › Single image/2E.tif]

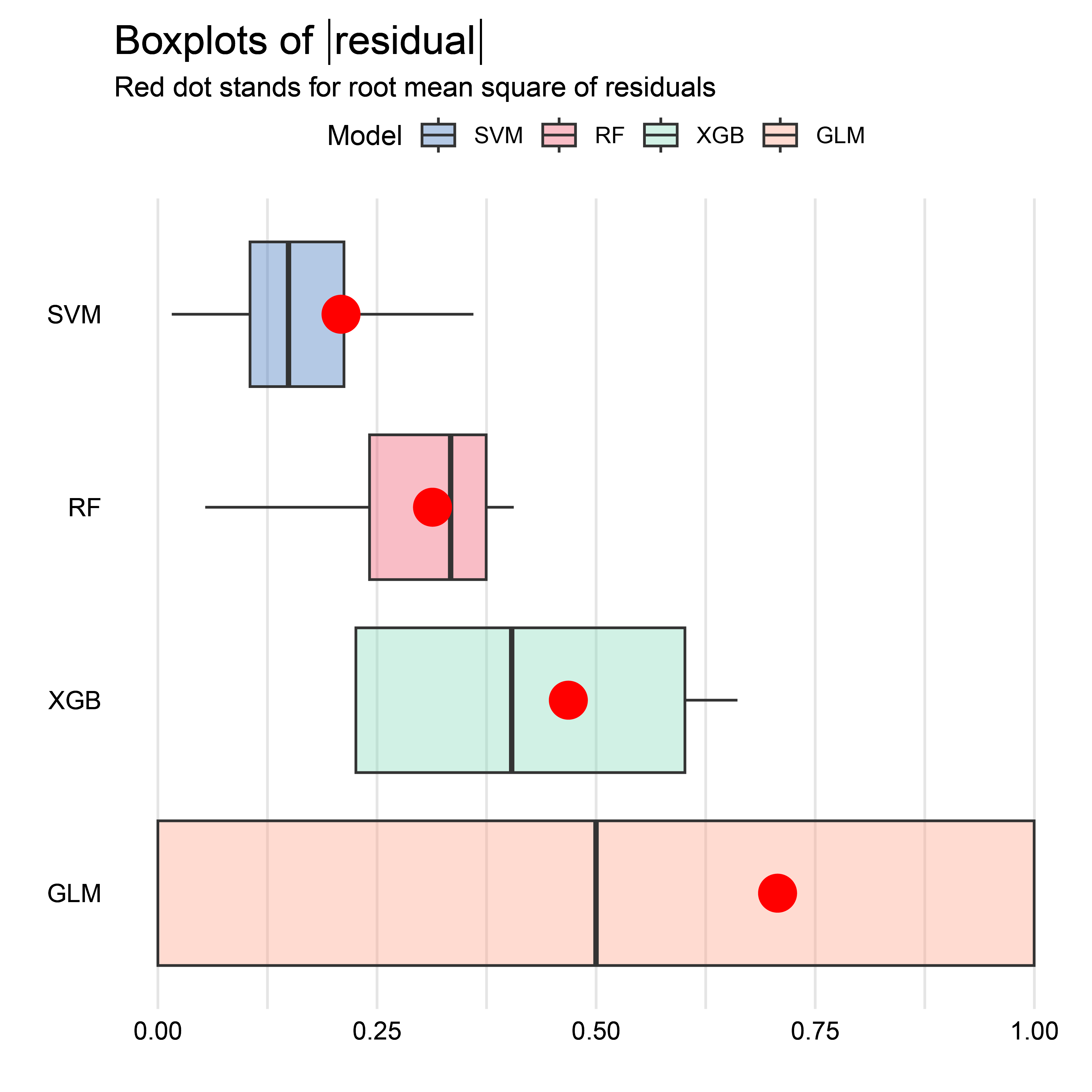

Supplement: Multimedia component 3 [file mmc3.zip › Single image/3A.tif]

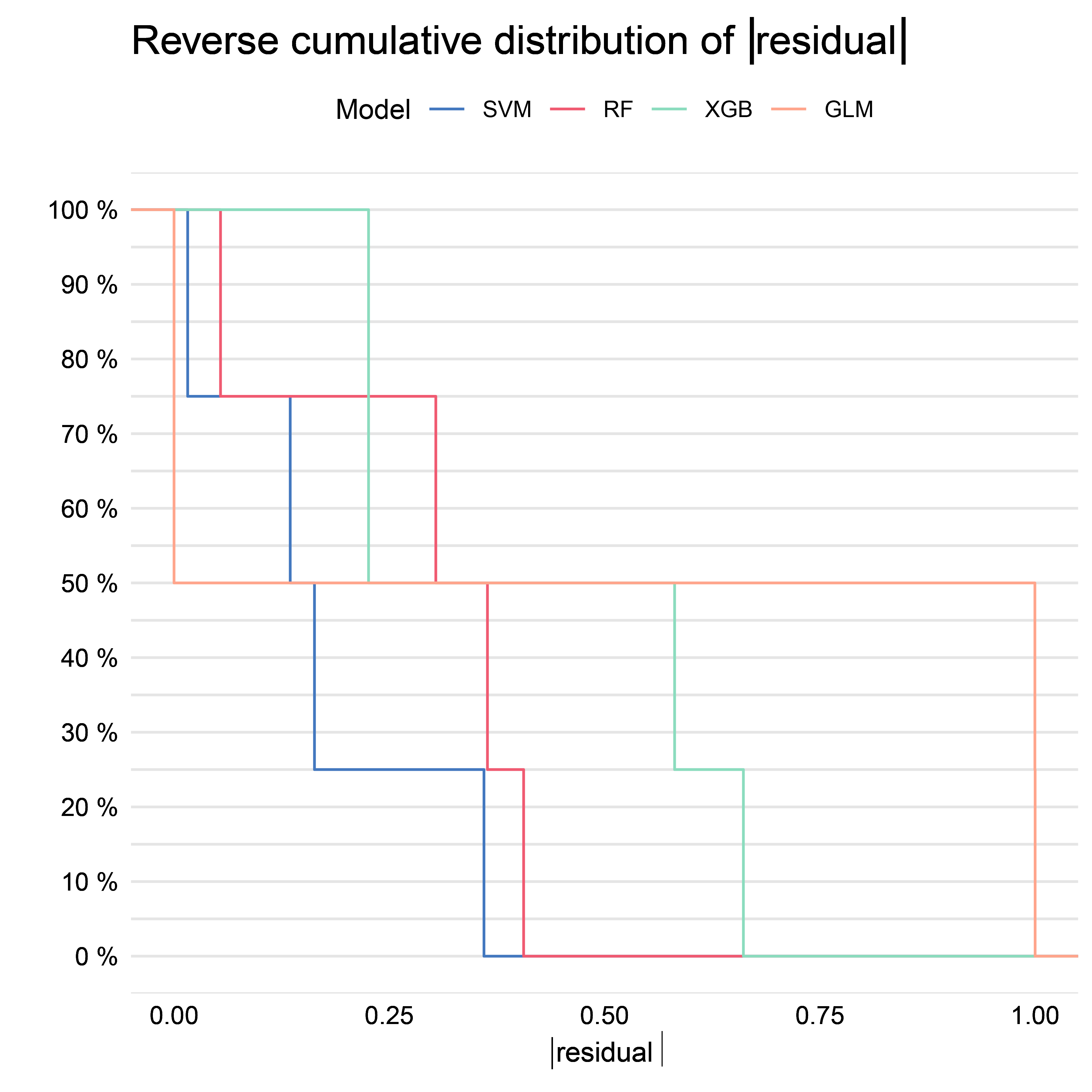

Supplement: Multimedia component 3 [file mmc3.zip › Single image/3B.tif]

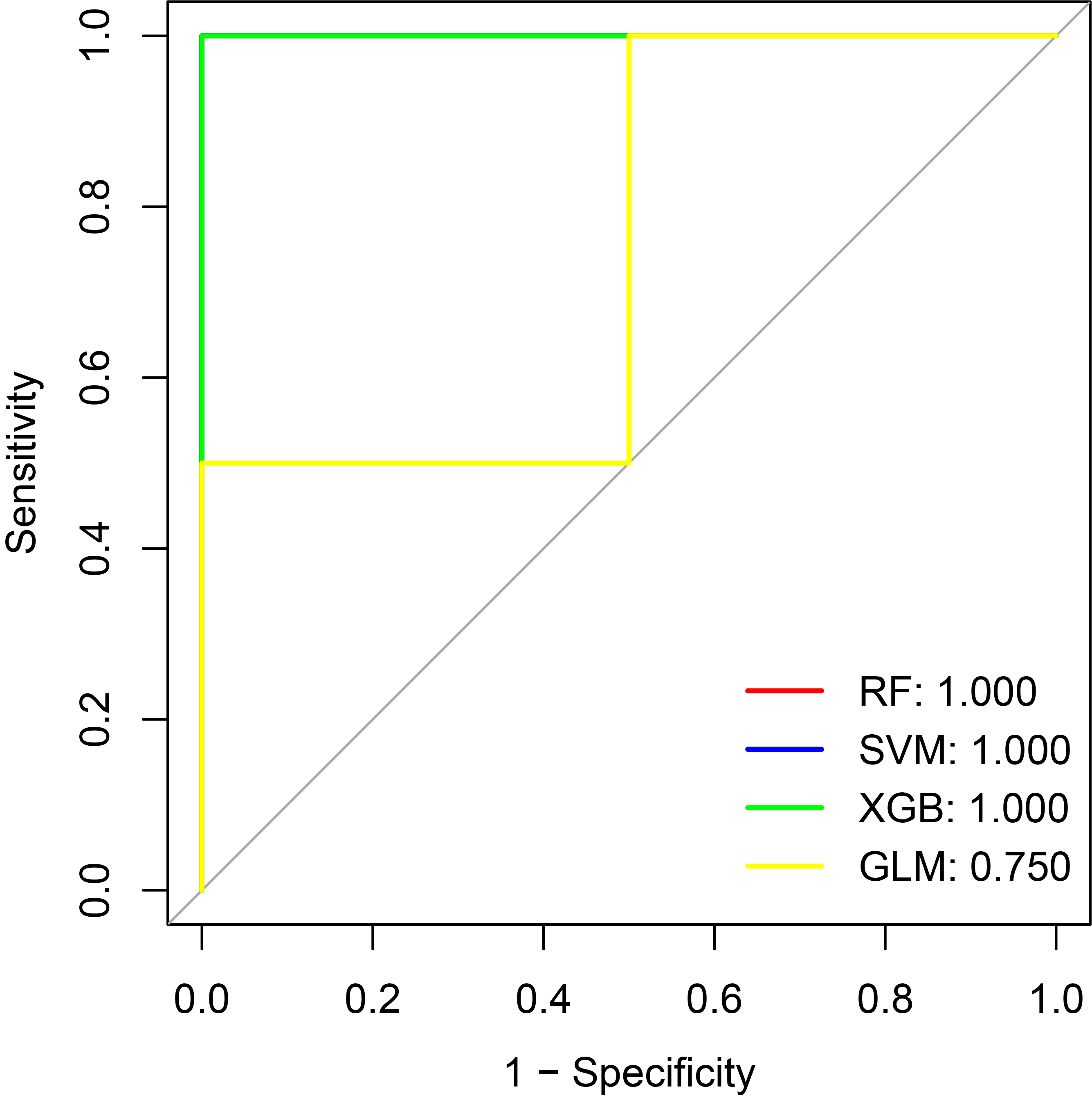

Supplement: Multimedia component 3 [file mmc3.zip › Single image/3C.tif]

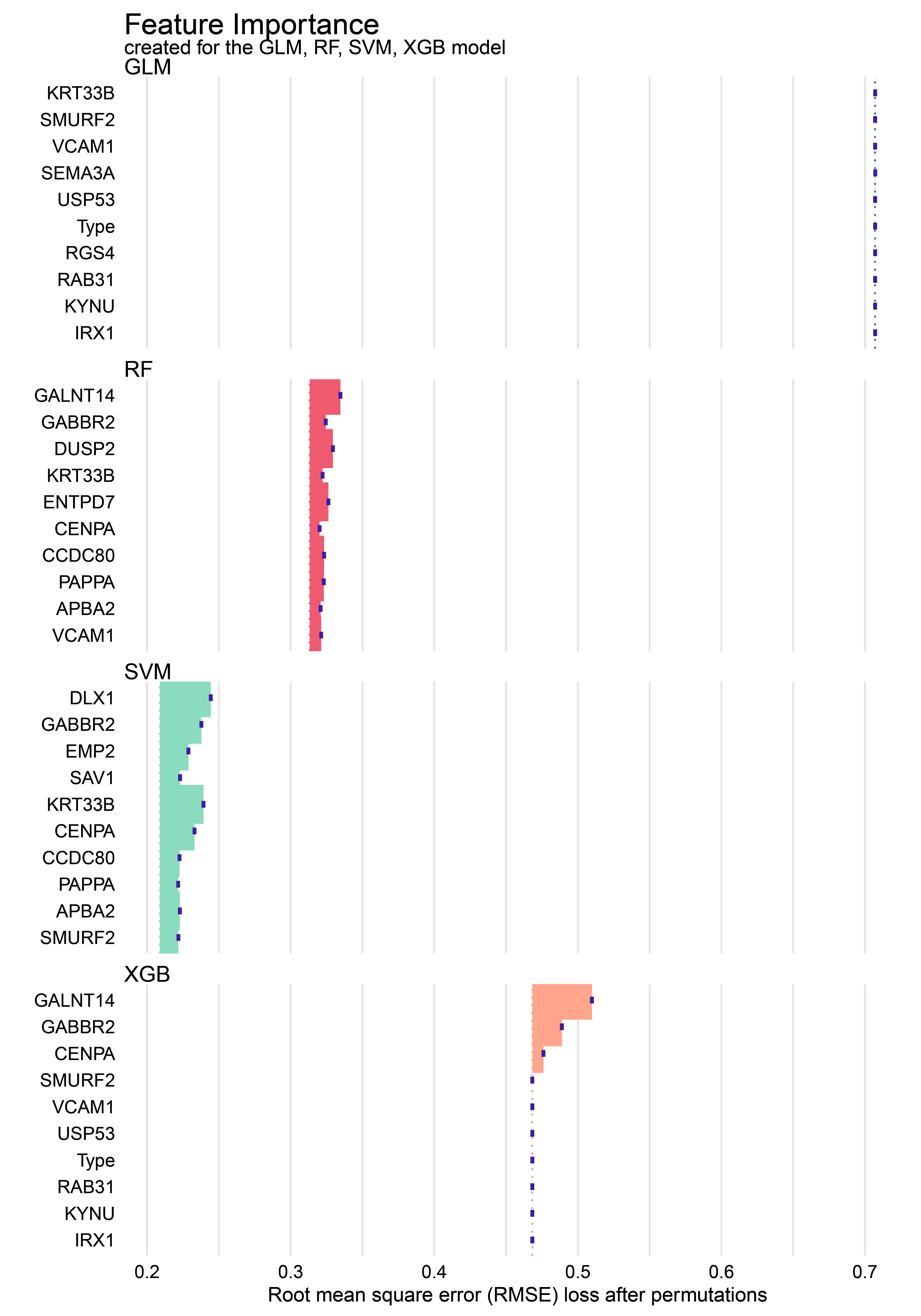

Supplement: Multimedia component 3 [file mmc3.zip › Single image/3D.tif]

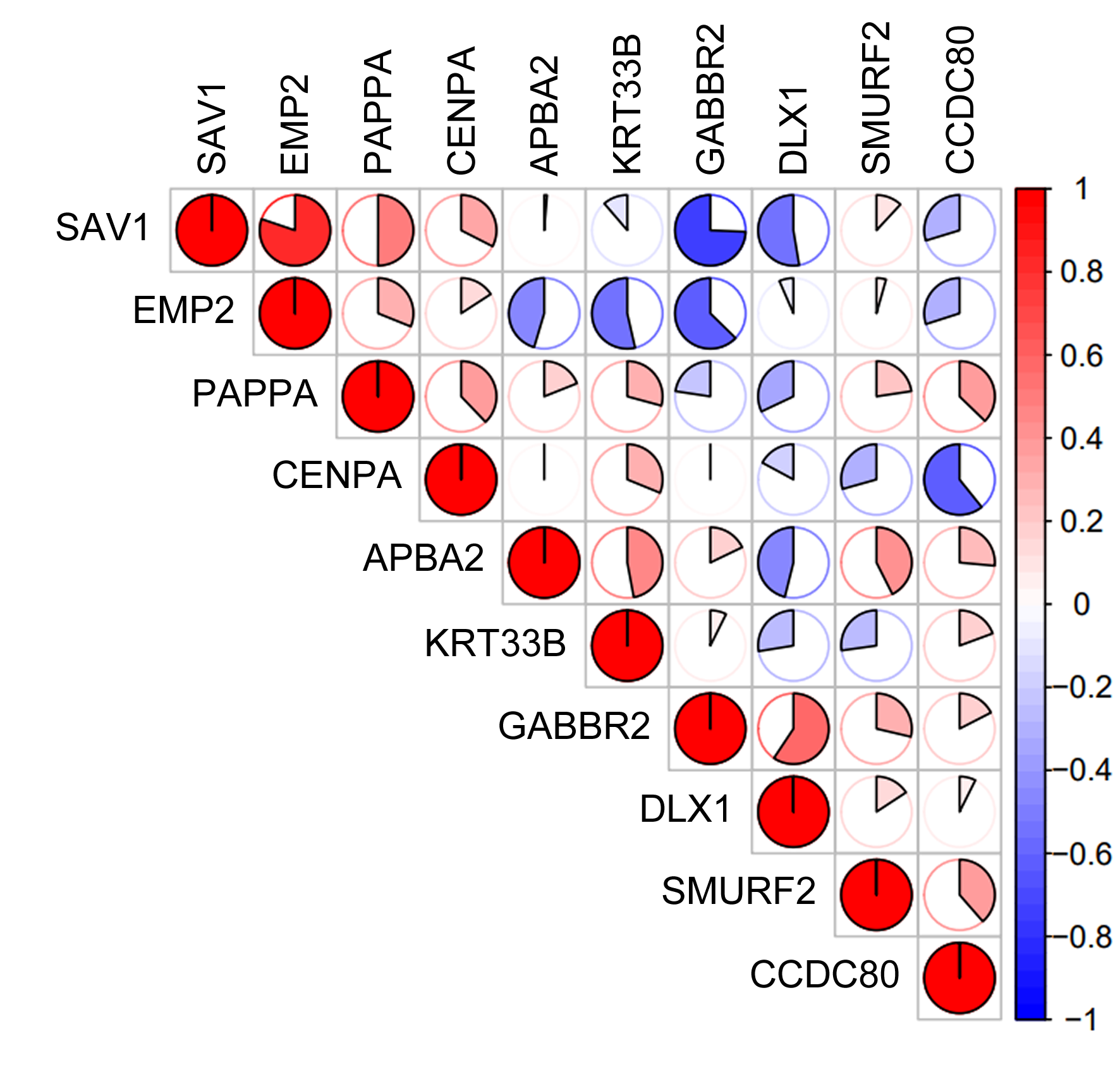

Supplement: Multimedia component 3 [file mmc3.zip › Single image/3E.tif]

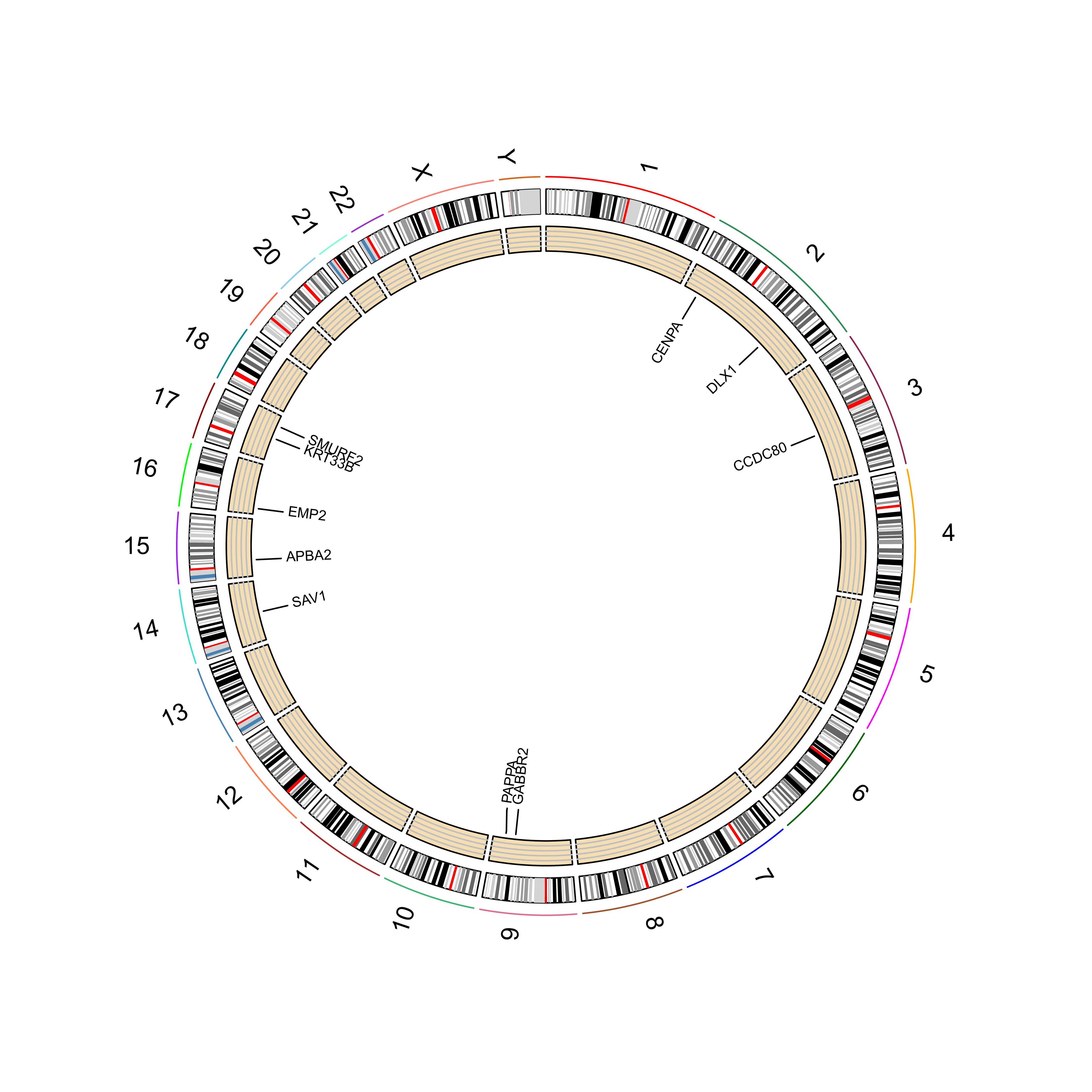

Supplement: Multimedia component 3 [file mmc3.zip › Single image/3F.tif]

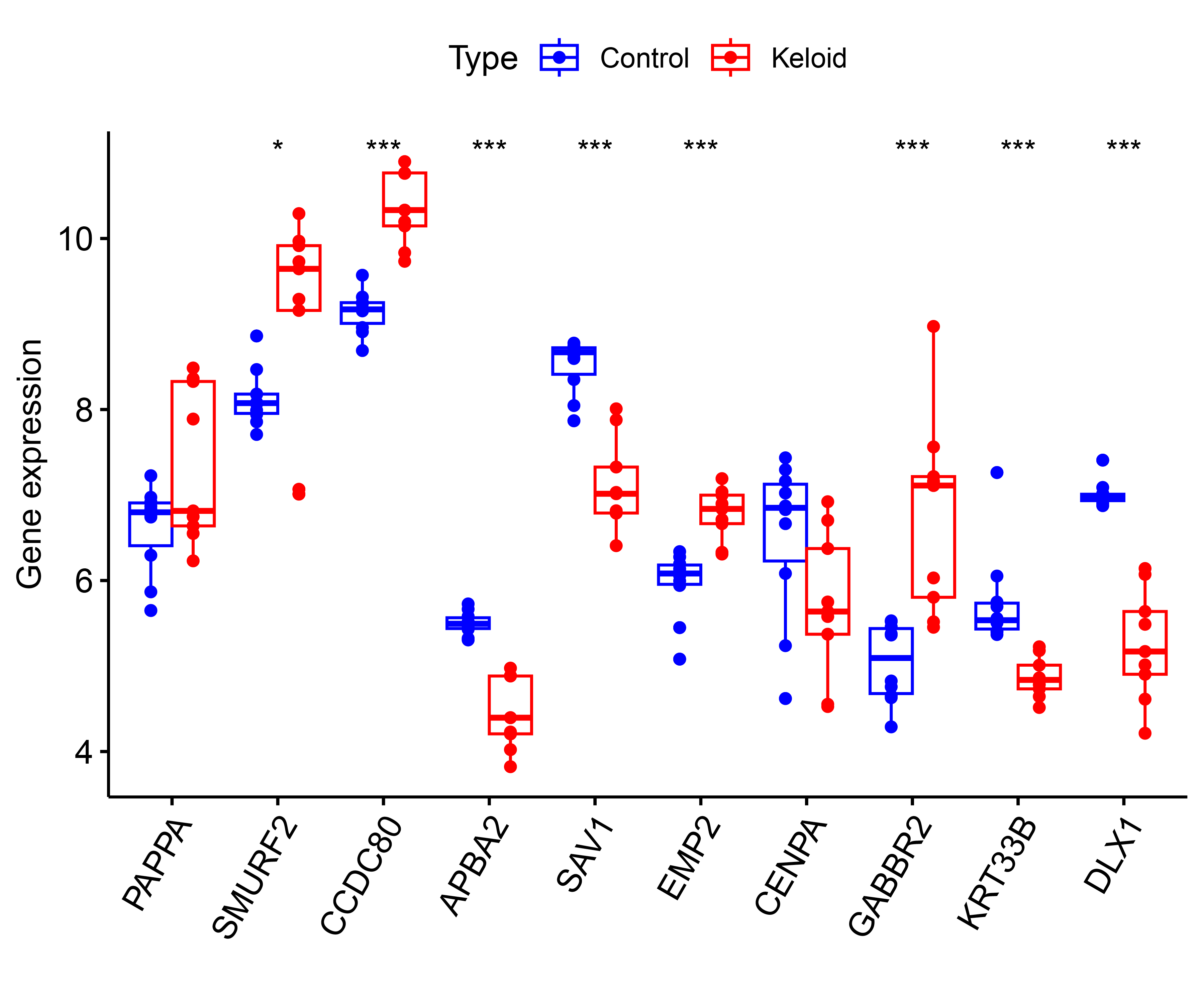

Supplement: Multimedia component 3 [file mmc3.zip › Single image/4A.tif]

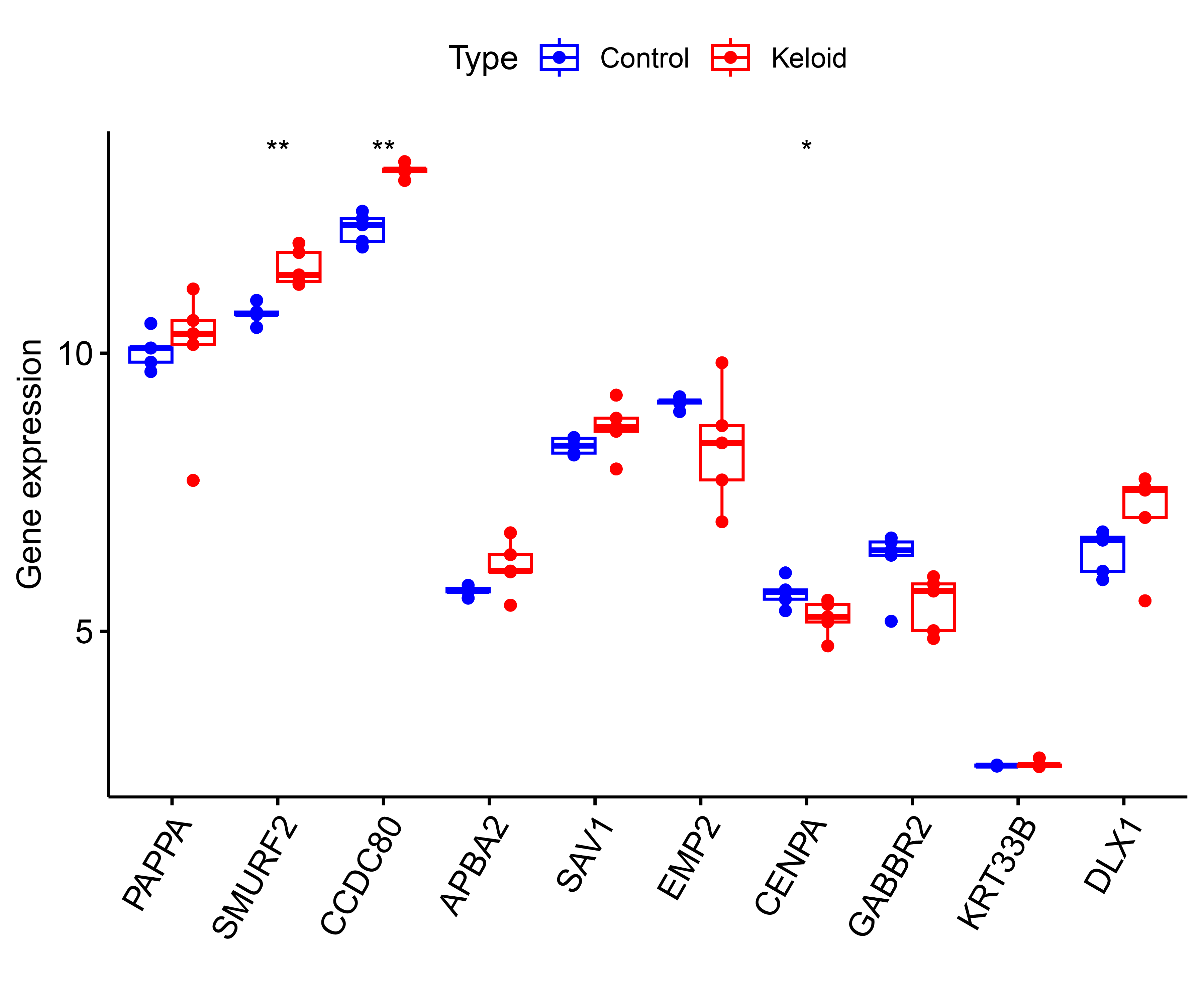

Supplement: Multimedia component 3 [file mmc3.zip › Single image/4B.tif]

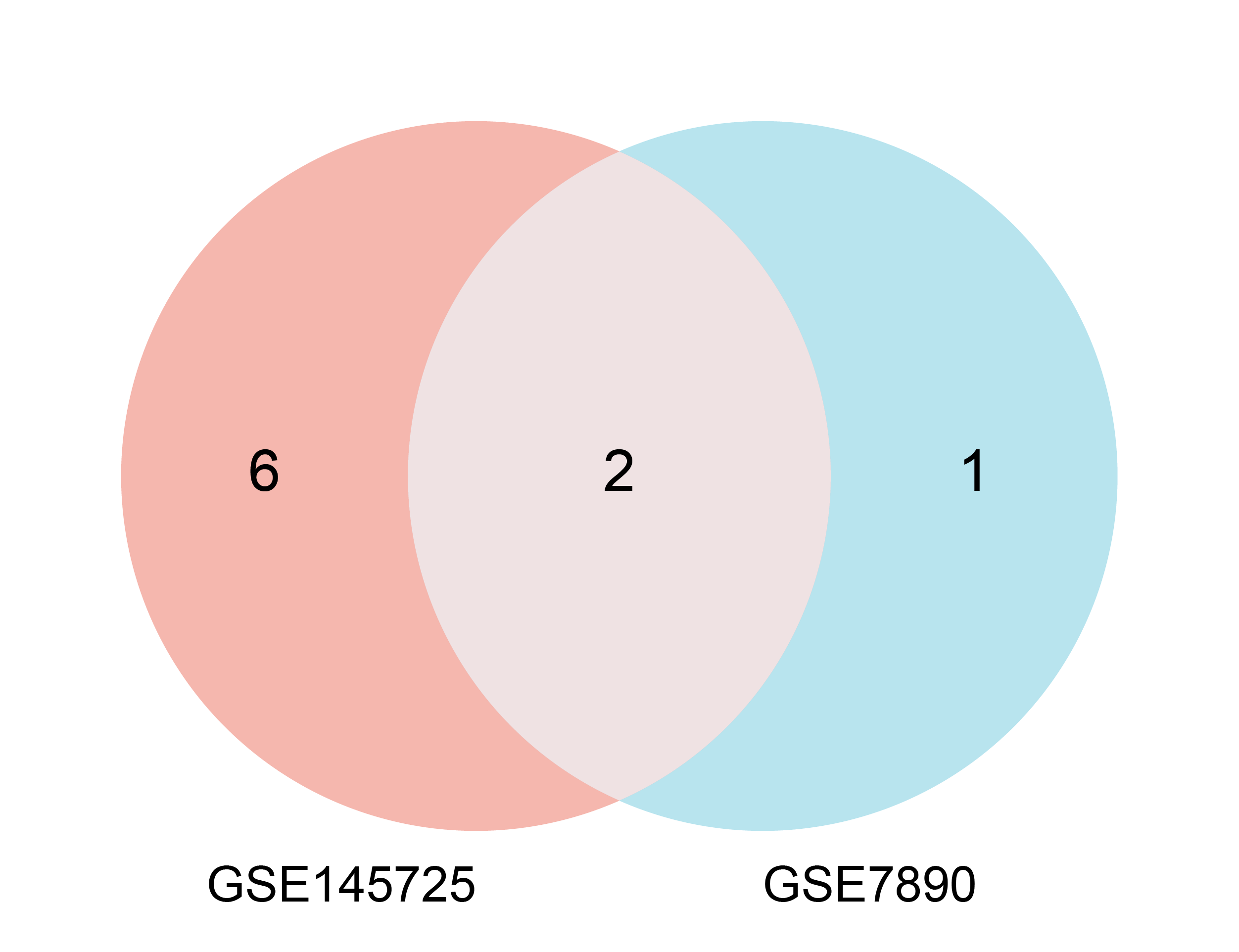

Supplement: Multimedia component 3 [file mmc3.zip › Single image/4C.tif]

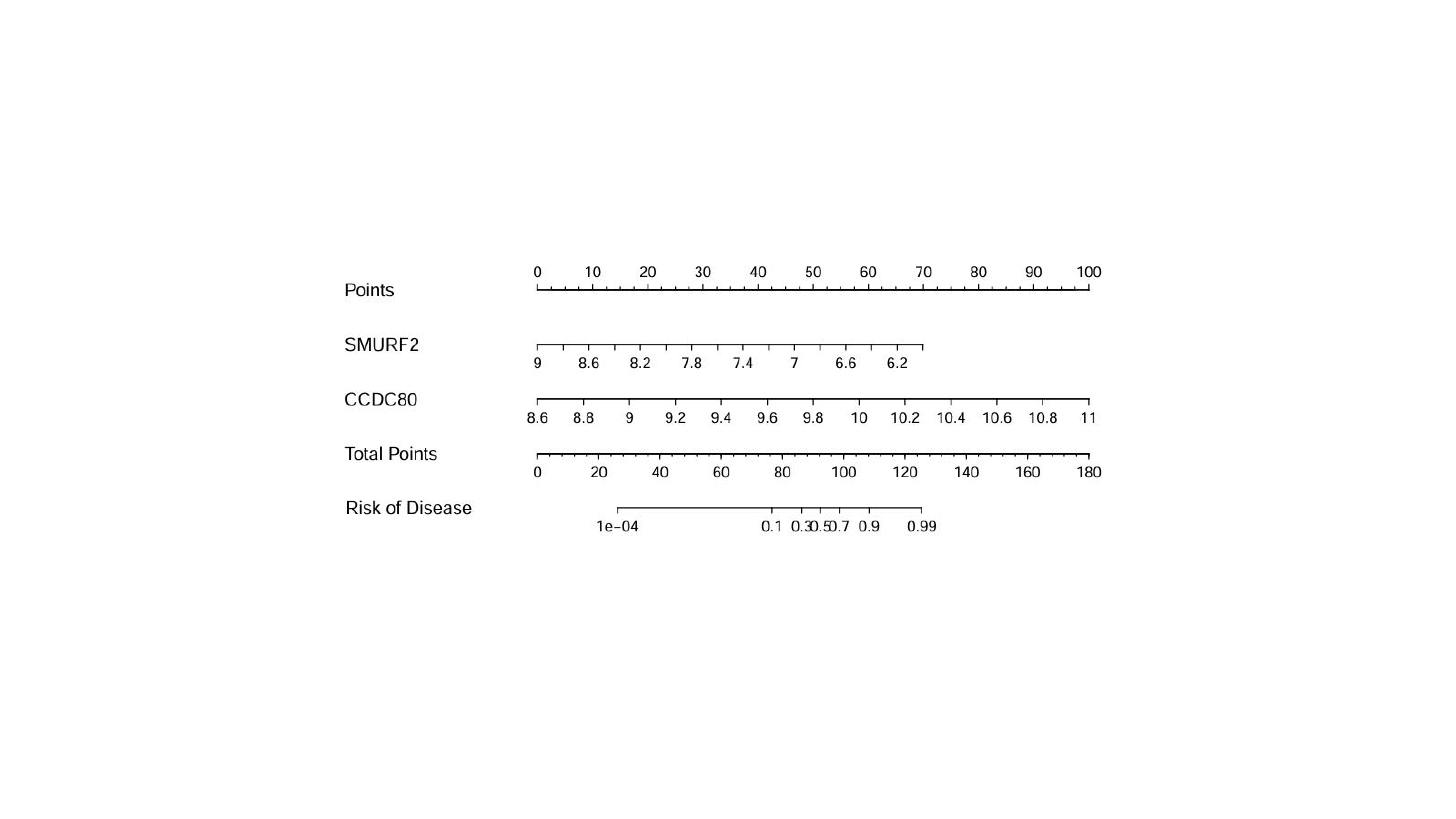

Supplement: Multimedia component 3 [file mmc3.zip › Single image/4D.tif]

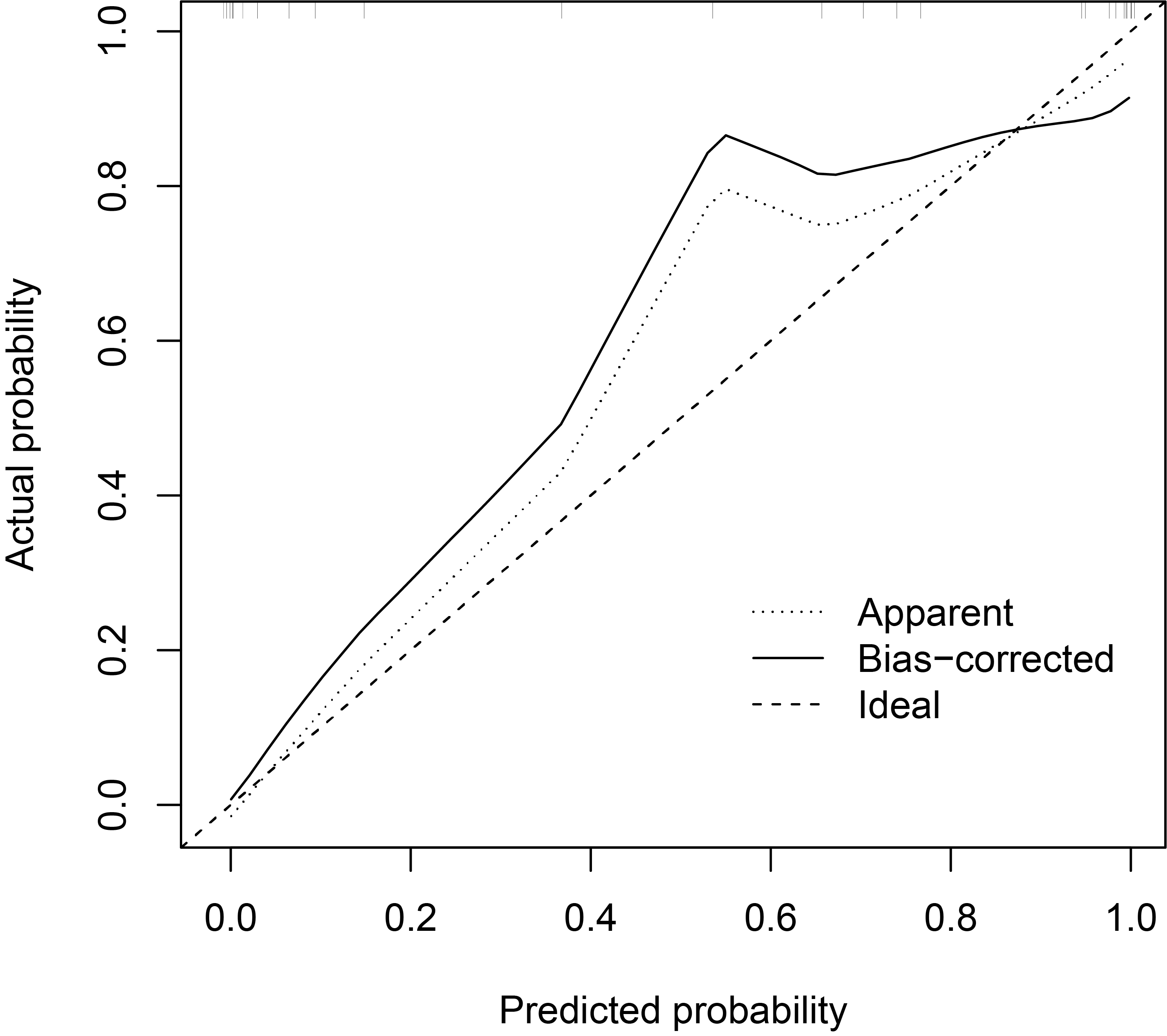

Supplement: Multimedia component 3 [file mmc3.zip › Single image/4E.tif]

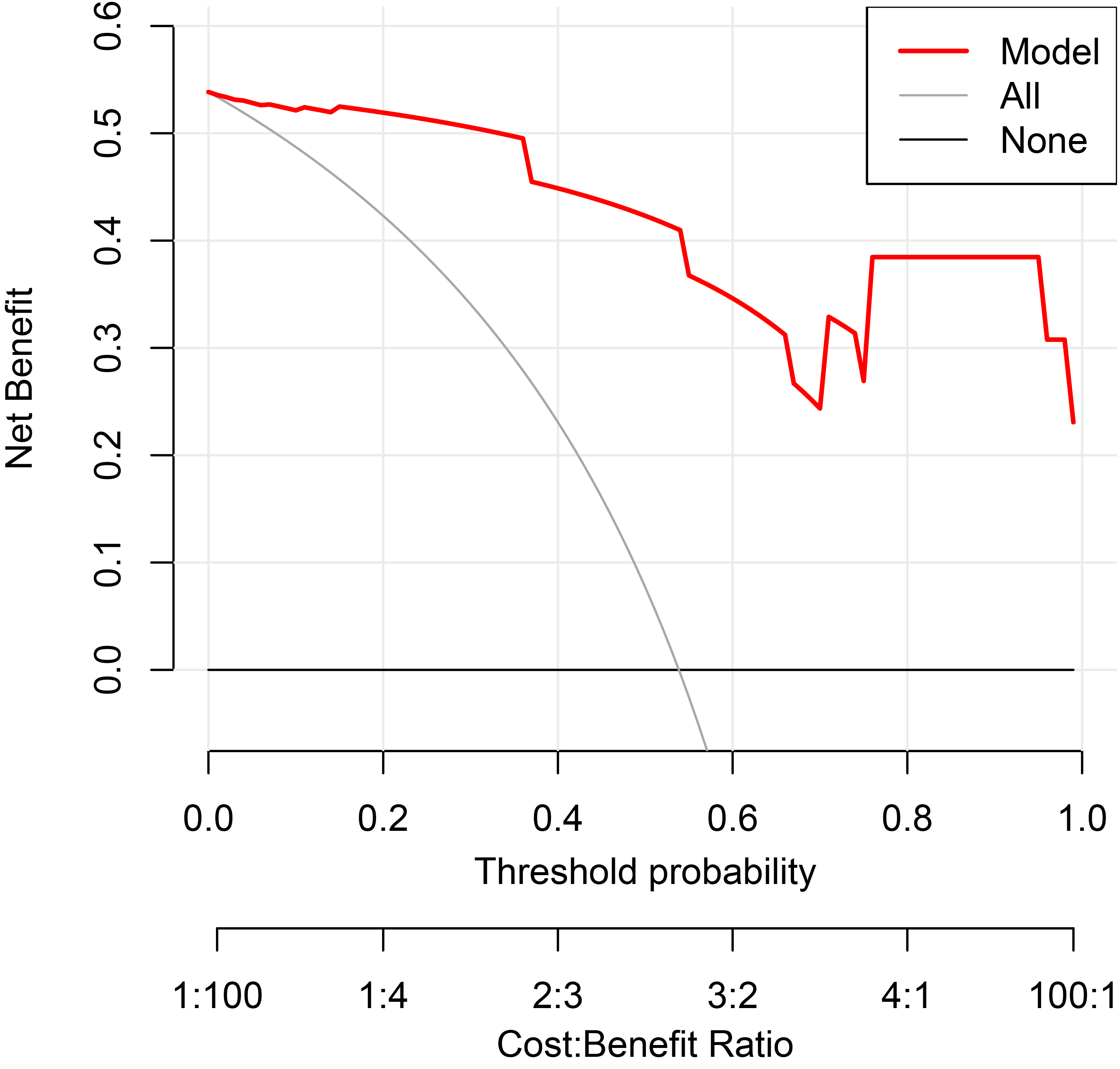

Supplement: Multimedia component 3 [file mmc3.zip › Single image/4F.tif]

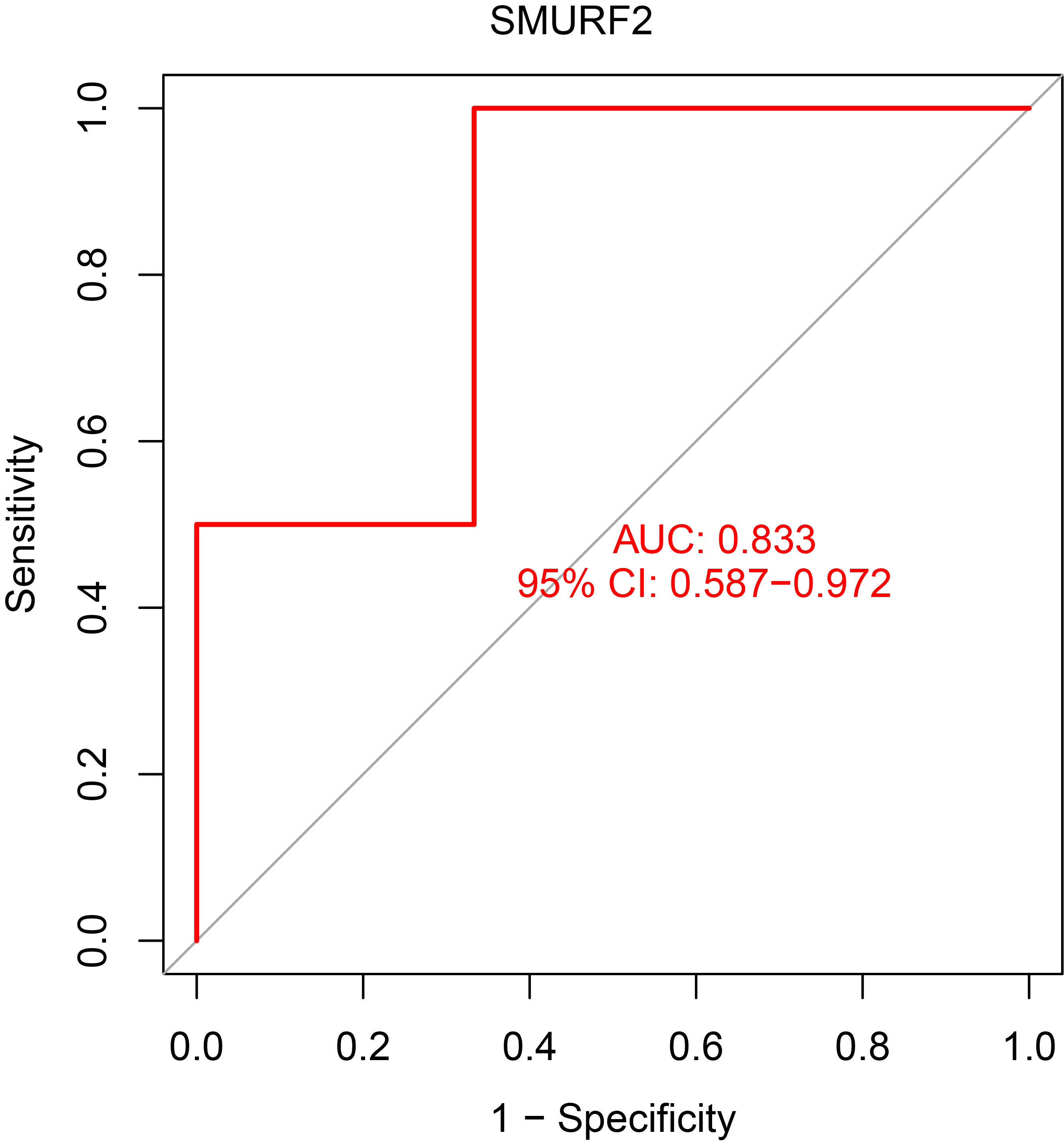

Supplement: Multimedia component 3 [file mmc3.zip › Single image/4G.tif]

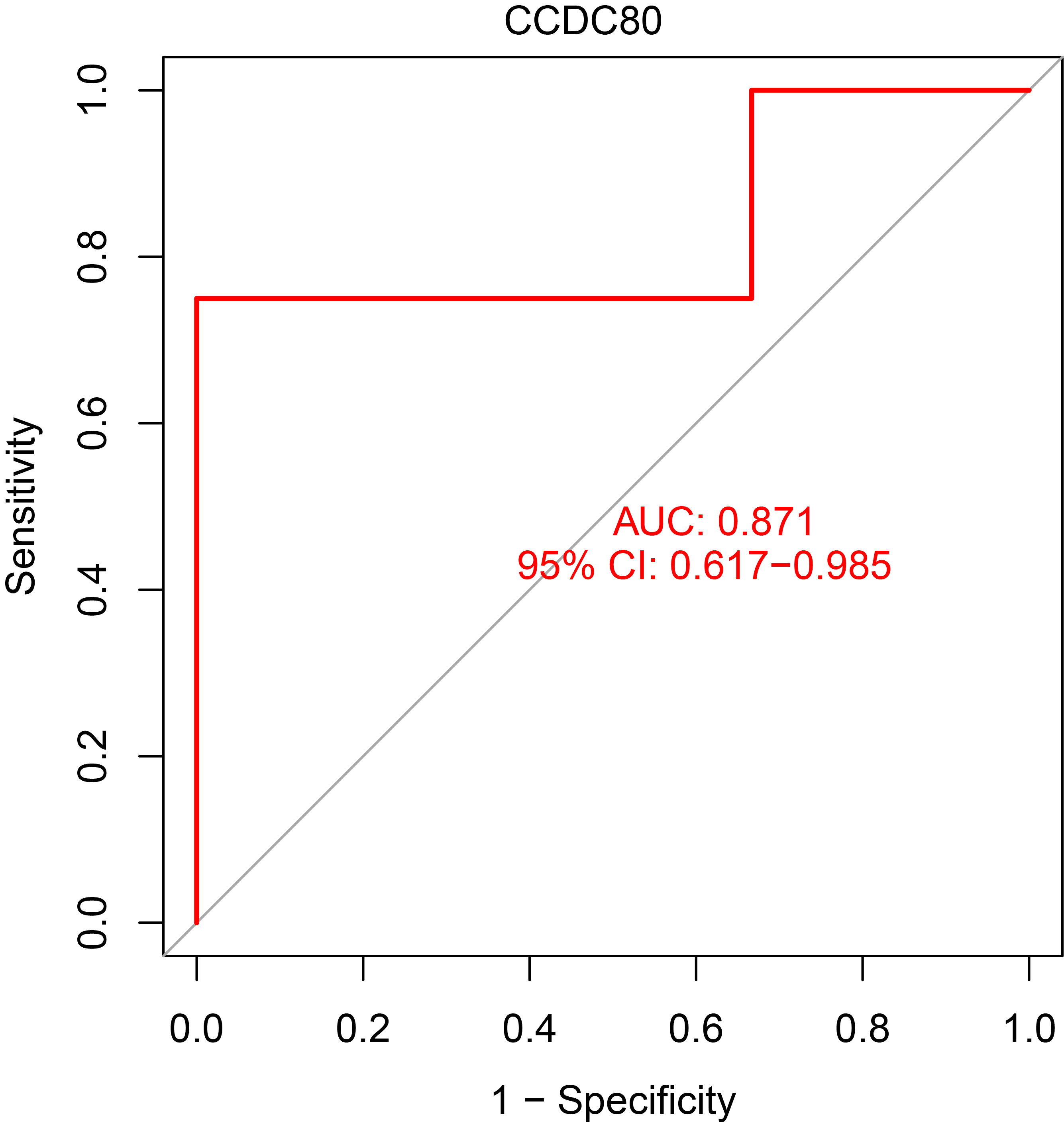

Supplement: Multimedia component 3 [file mmc3.zip › Single image/4H.tif]

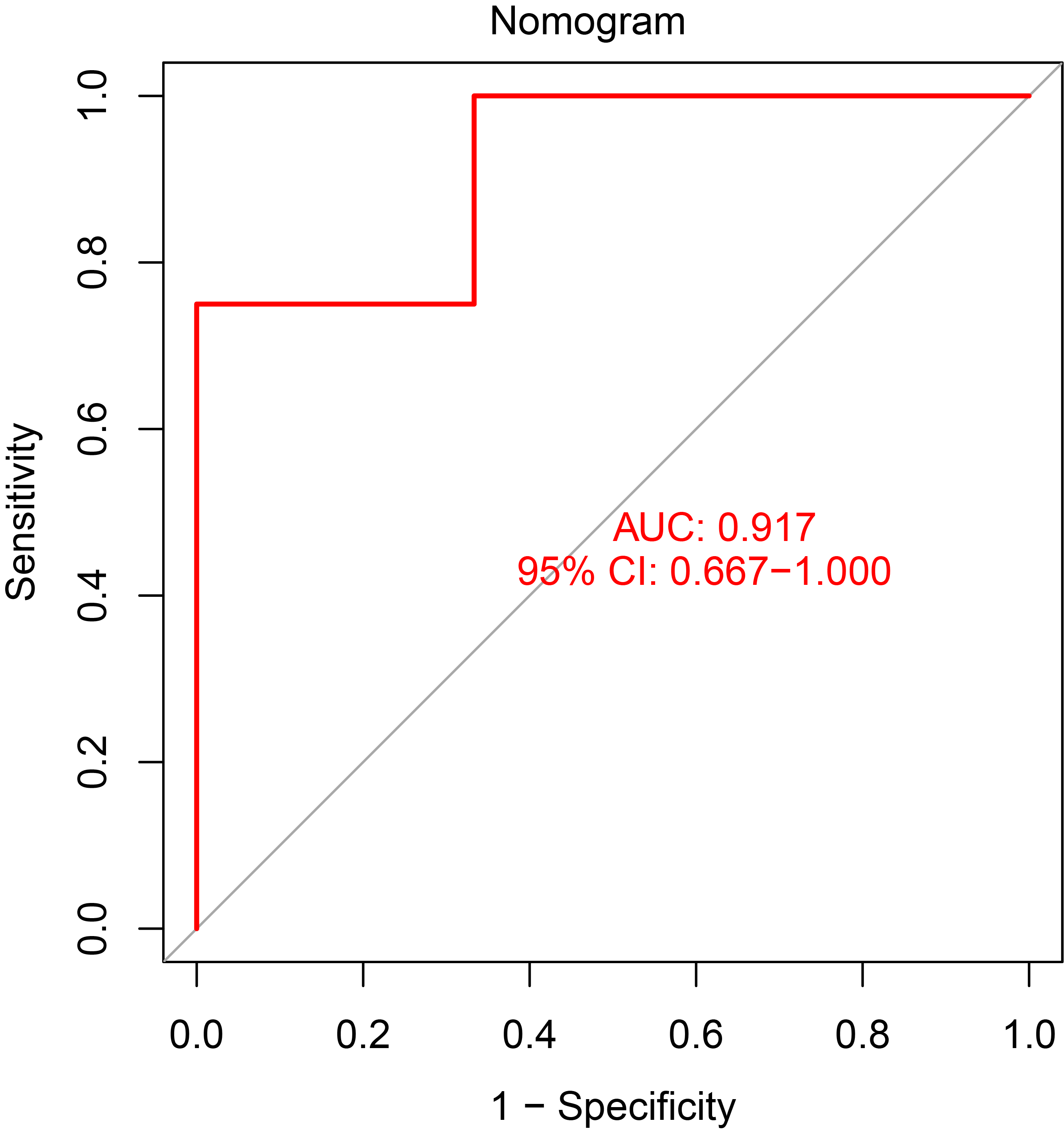

Supplement: Multimedia component 3 [file mmc3.zip › Single image/4I.tif]

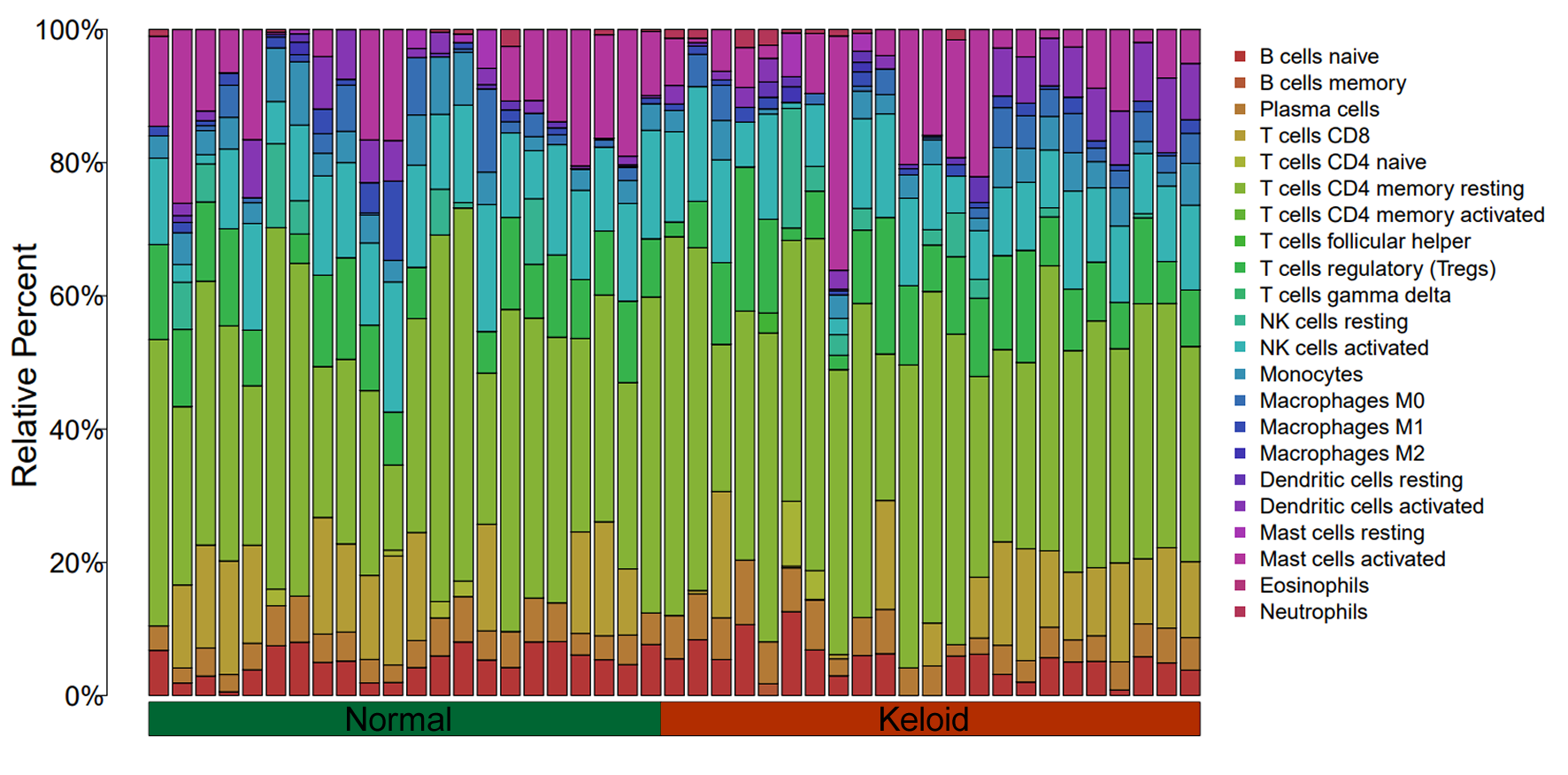

Supplement: Multimedia component 3 [file mmc3.zip › Single image/5A.tif]

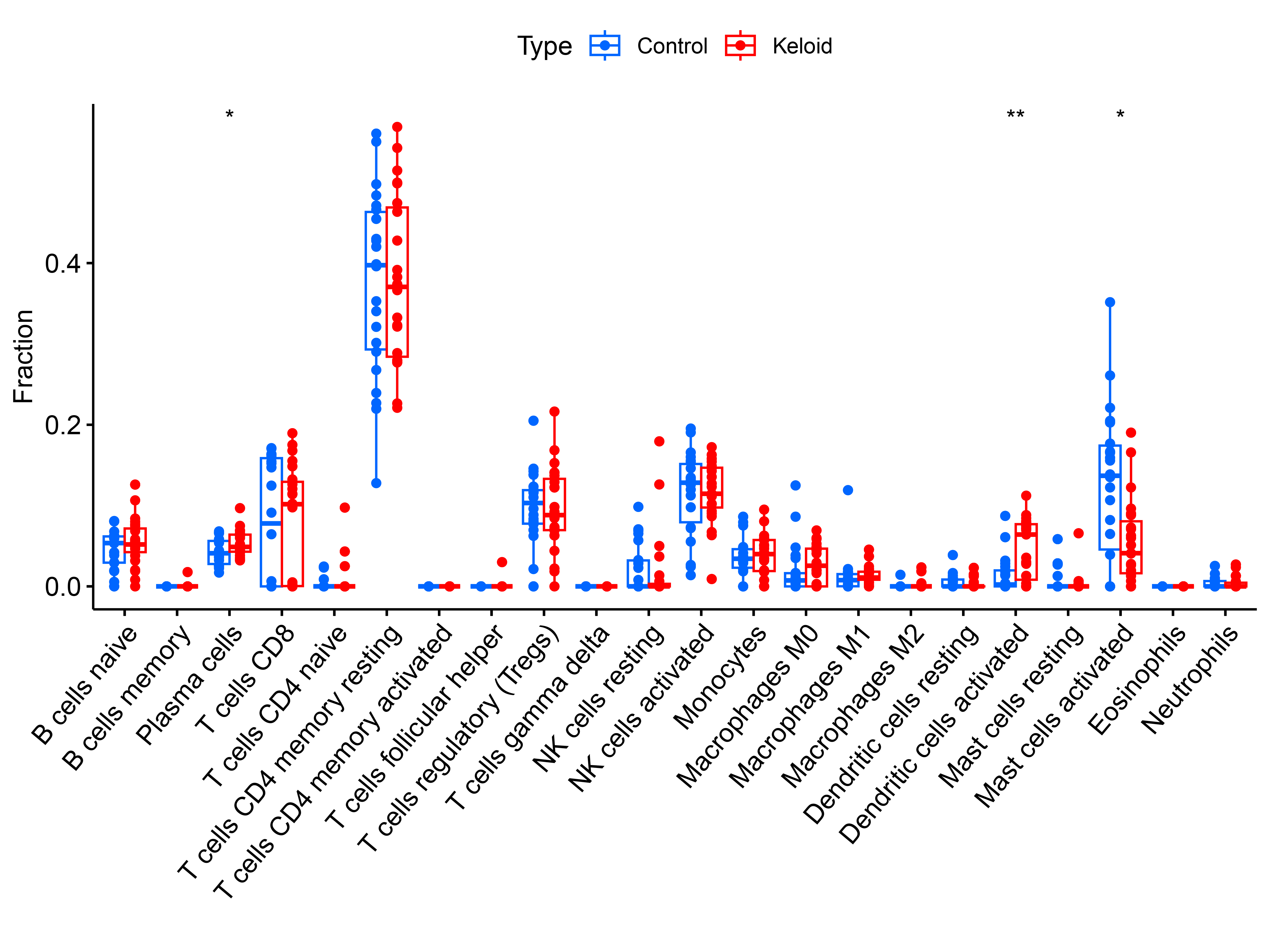

Supplement: Multimedia component 3 [file mmc3.zip › Single image/5B.tif]

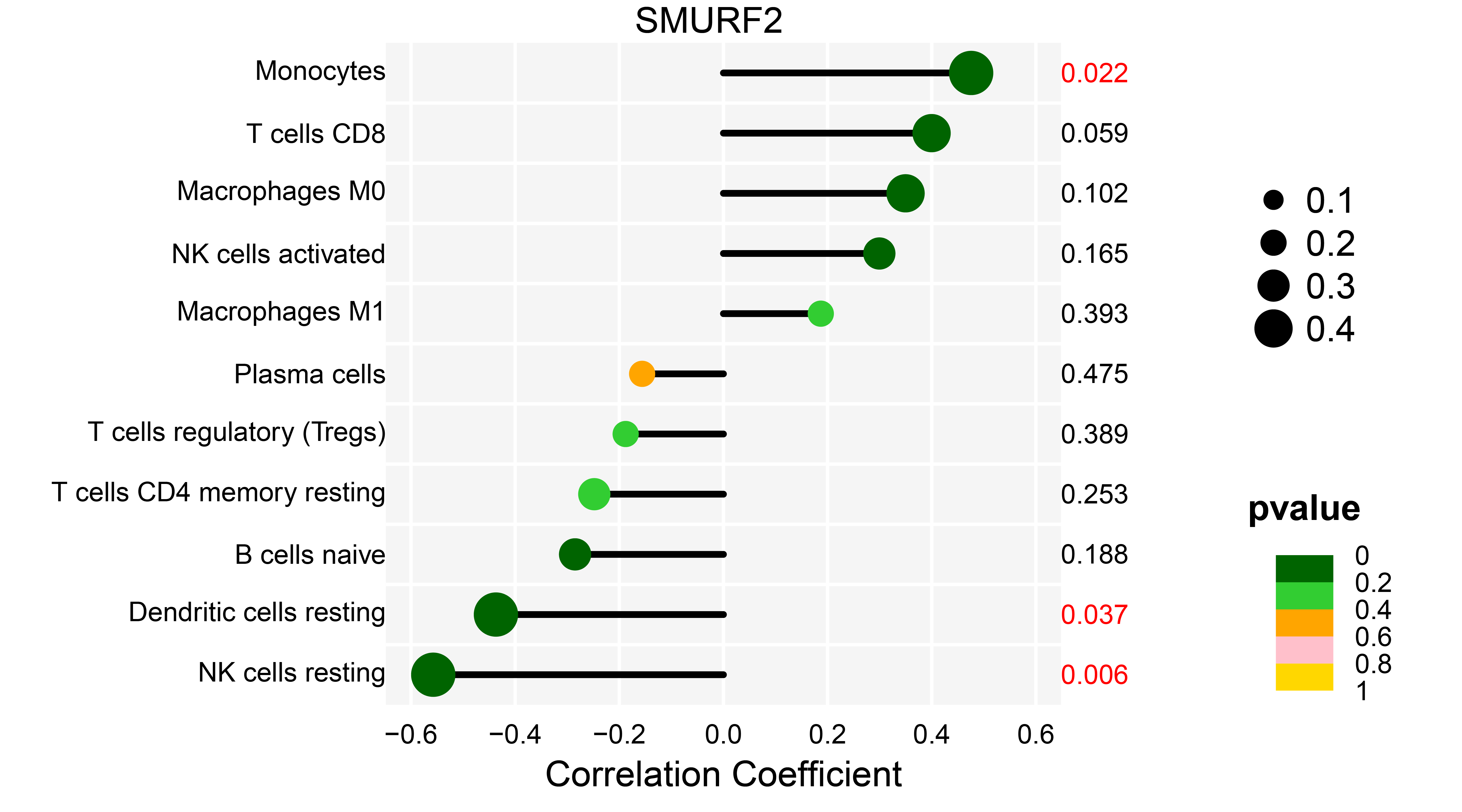

Supplement: Multimedia component 3 [file mmc3.zip › Single image/5C.tif]

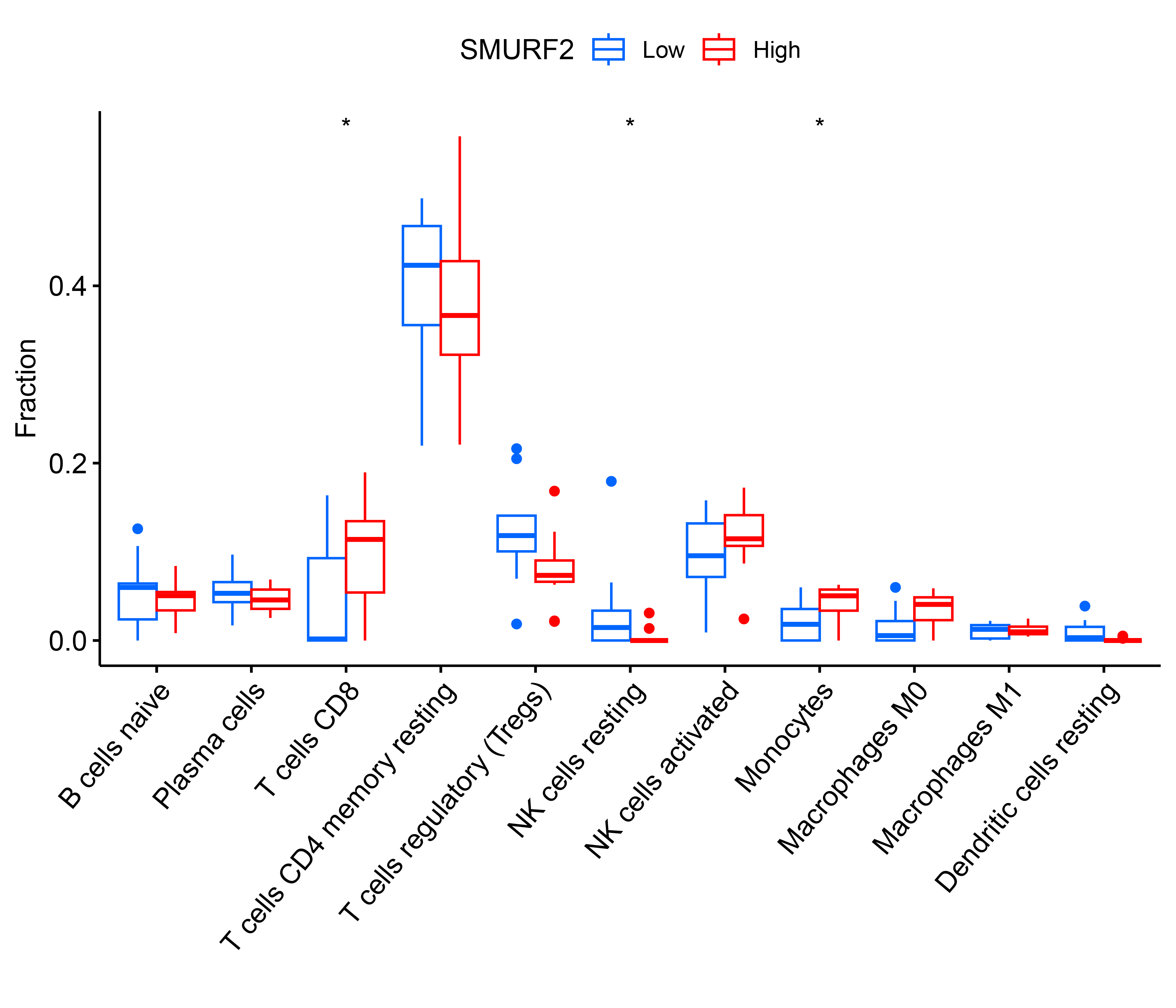

Supplement: Multimedia component 3 [file mmc3.zip › Single image/5D.tif]

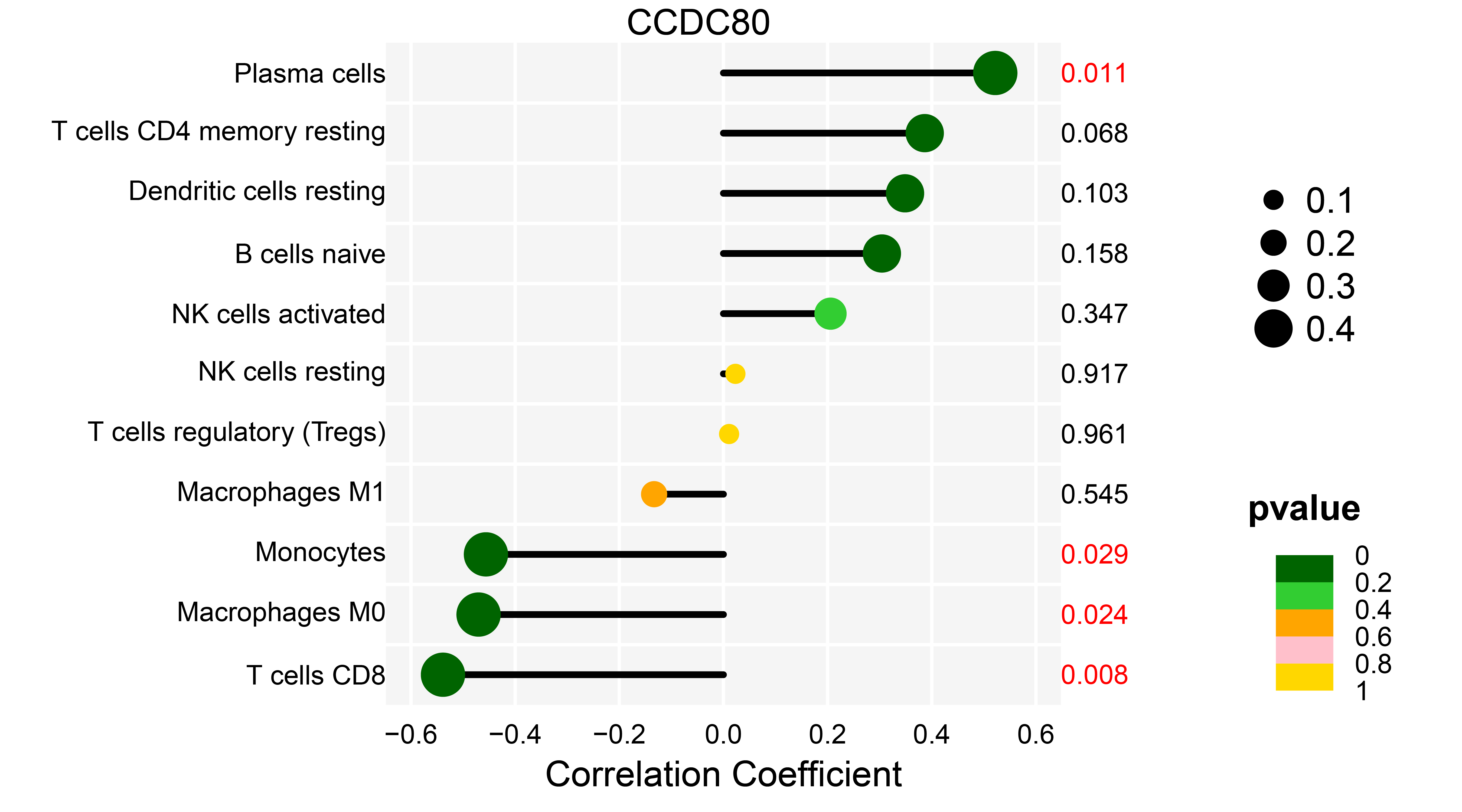

Supplement: Multimedia component 3 [file mmc3.zip › Single image/5E.tif]

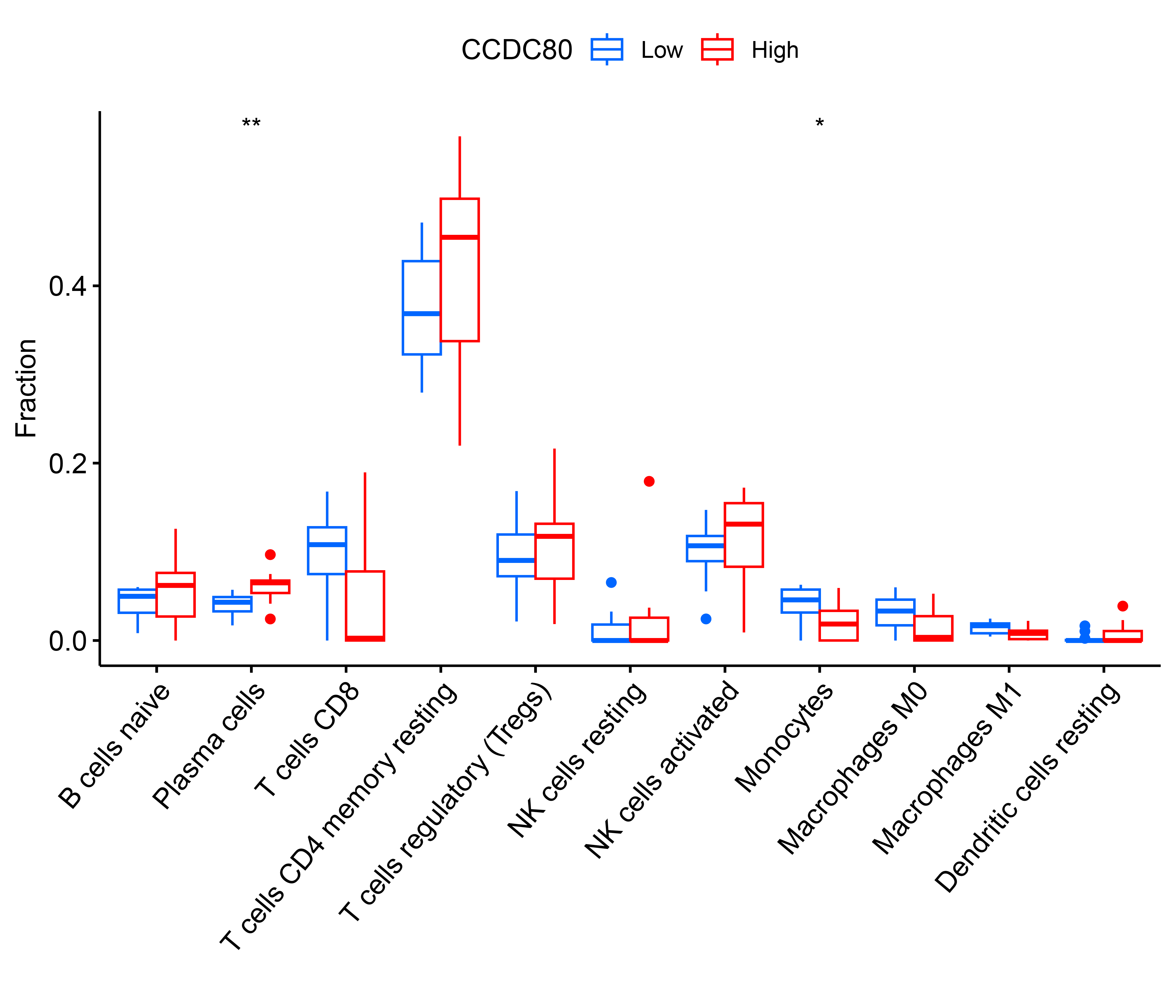

Supplement: Multimedia component 3 [file mmc3.zip › Single image/5F.tif]

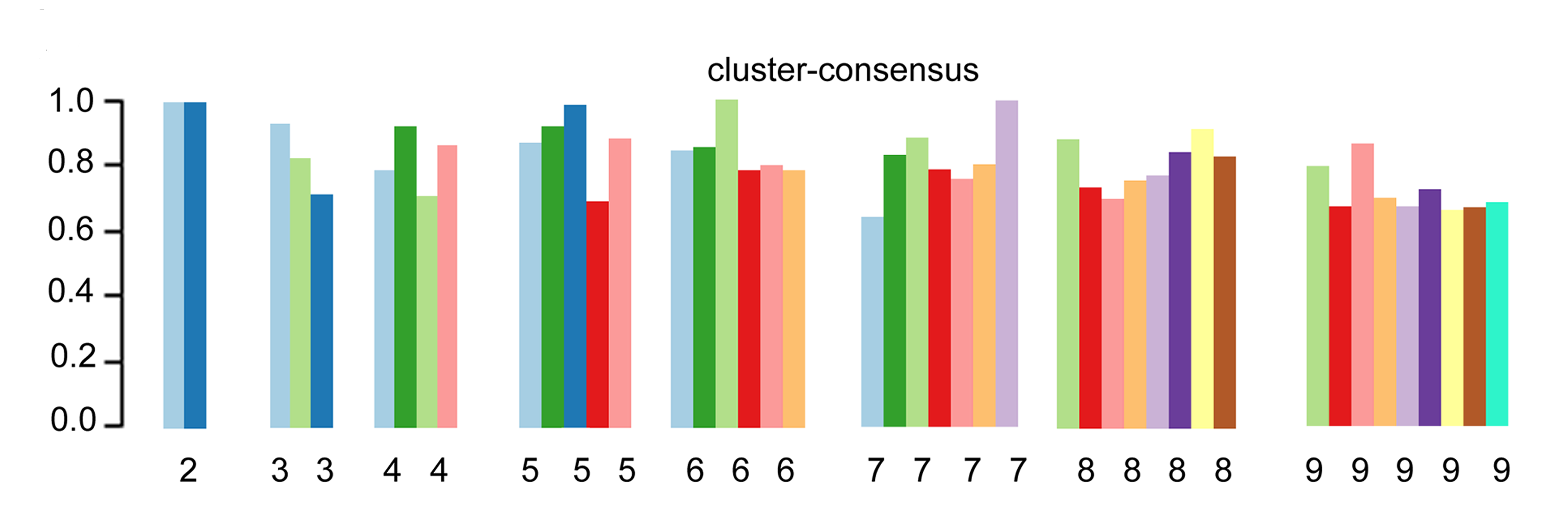

Supplement: Multimedia component 3 [file mmc3.zip › Single image/6D.tif]

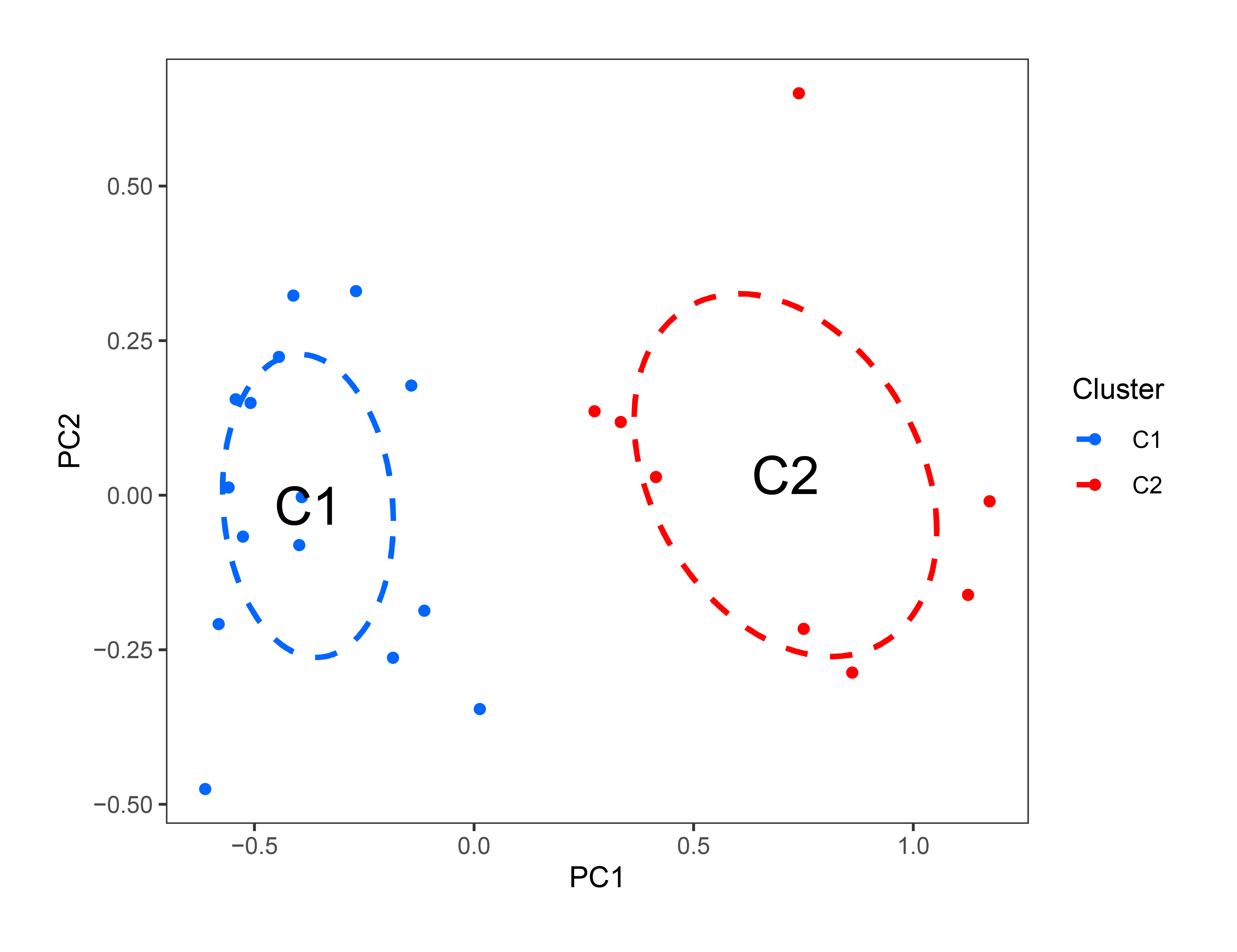

Supplement: Multimedia component 3 [file mmc3.zip › Single image/6E.tif]

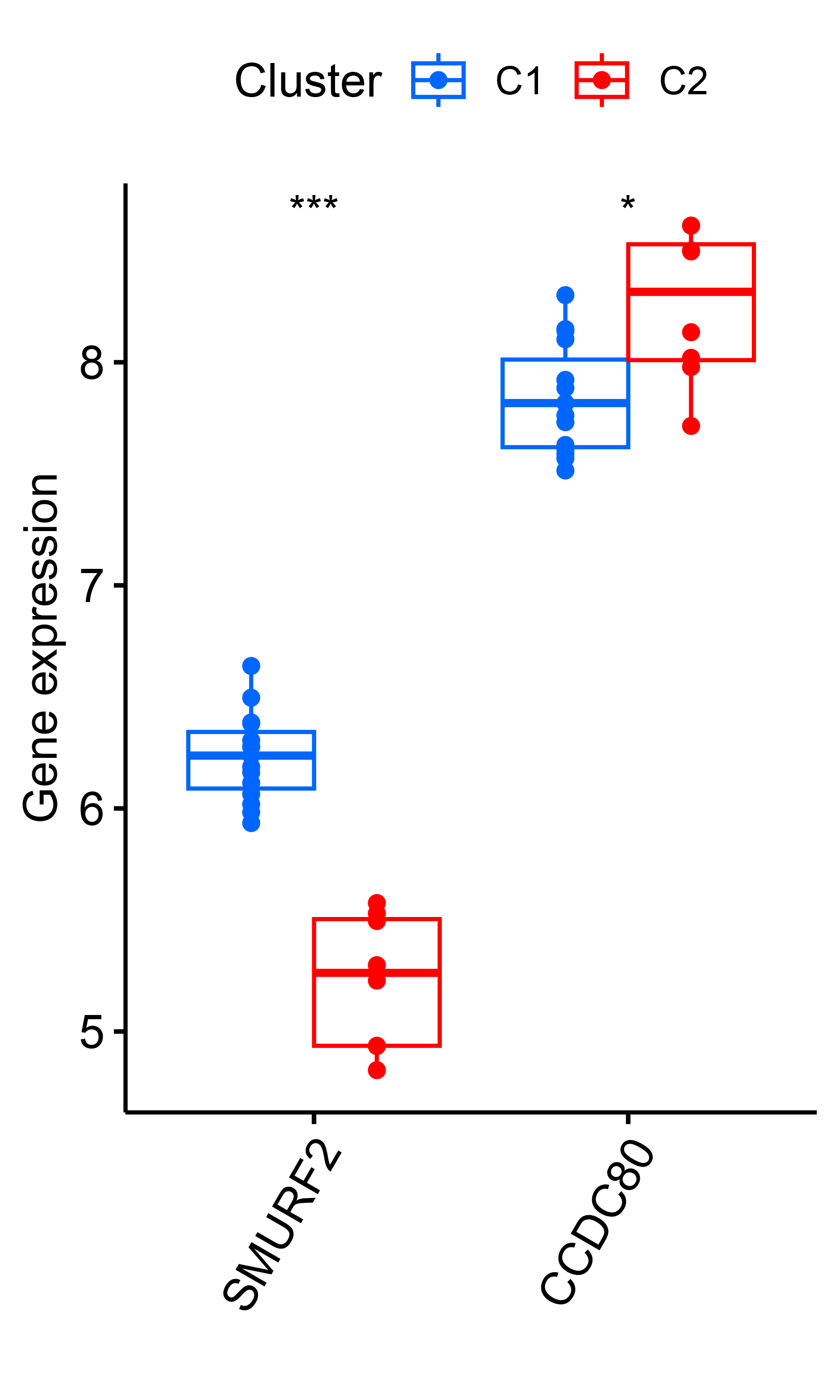

Supplement: Multimedia component 3 [file mmc3.zip › Single image/6F.tif]

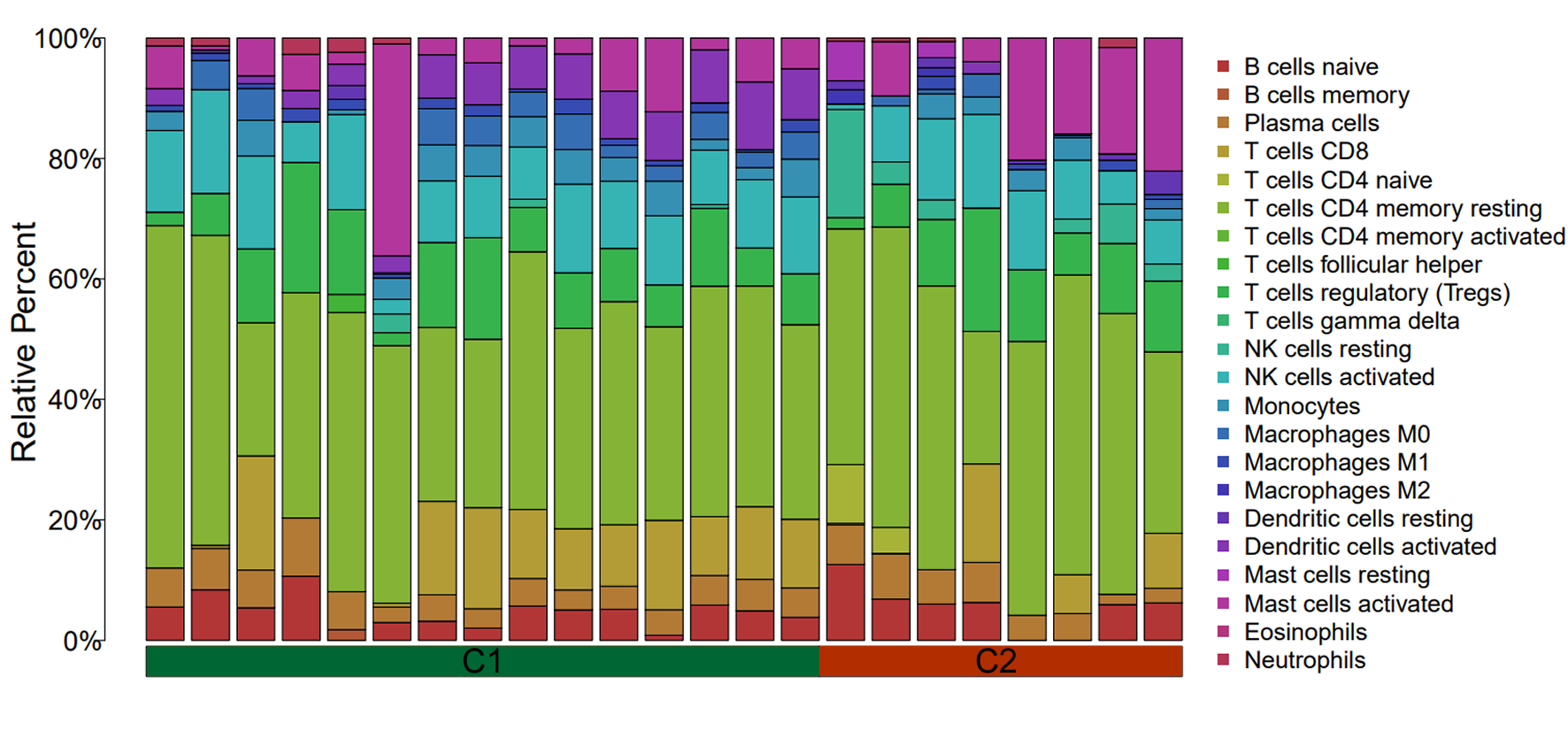

Supplement: Multimedia component 3 [file mmc3.zip › Single image/7A.tif]

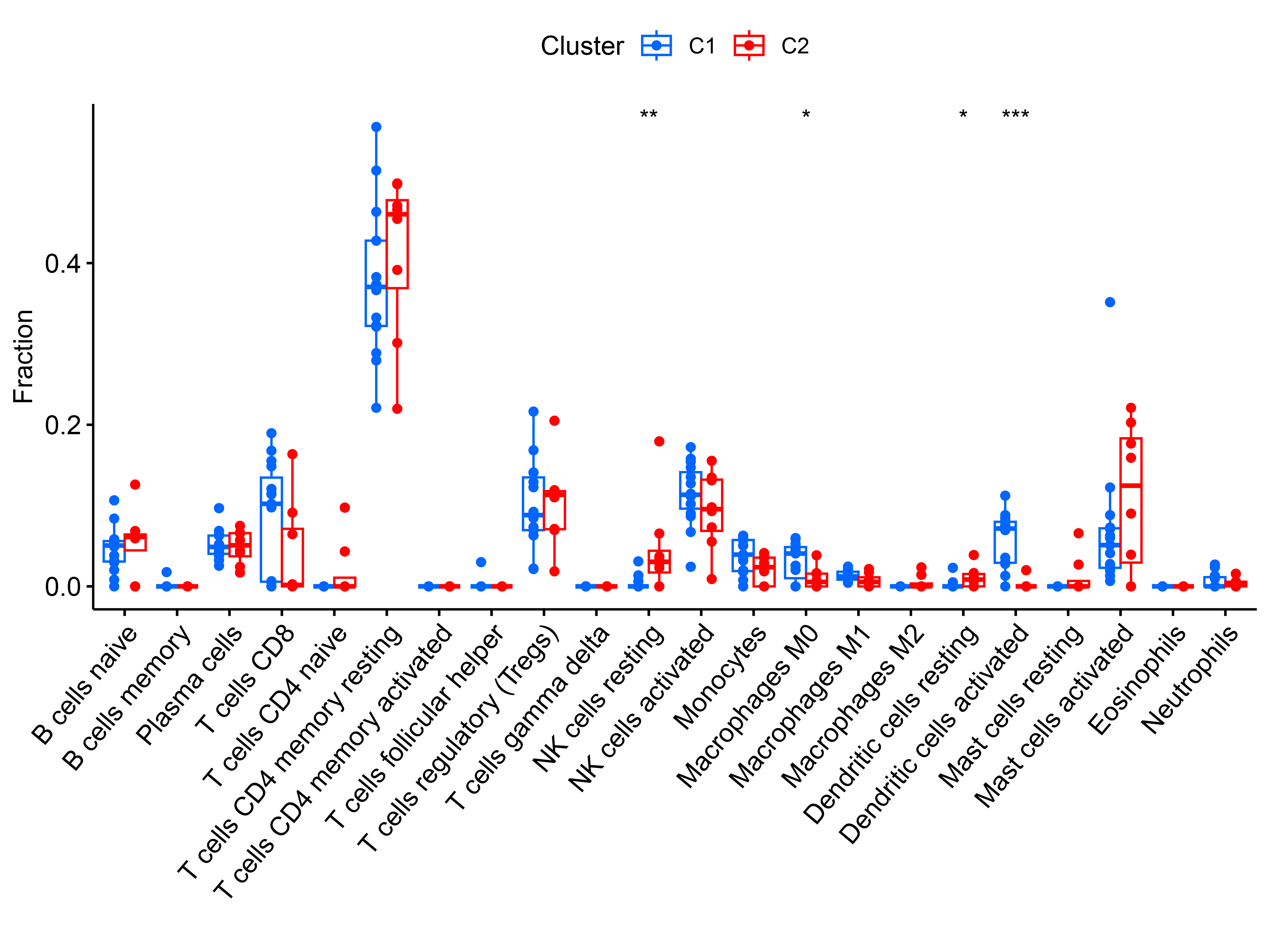

Supplement: Multimedia component 3 [file mmc3.zip › Single image/7B.tif]

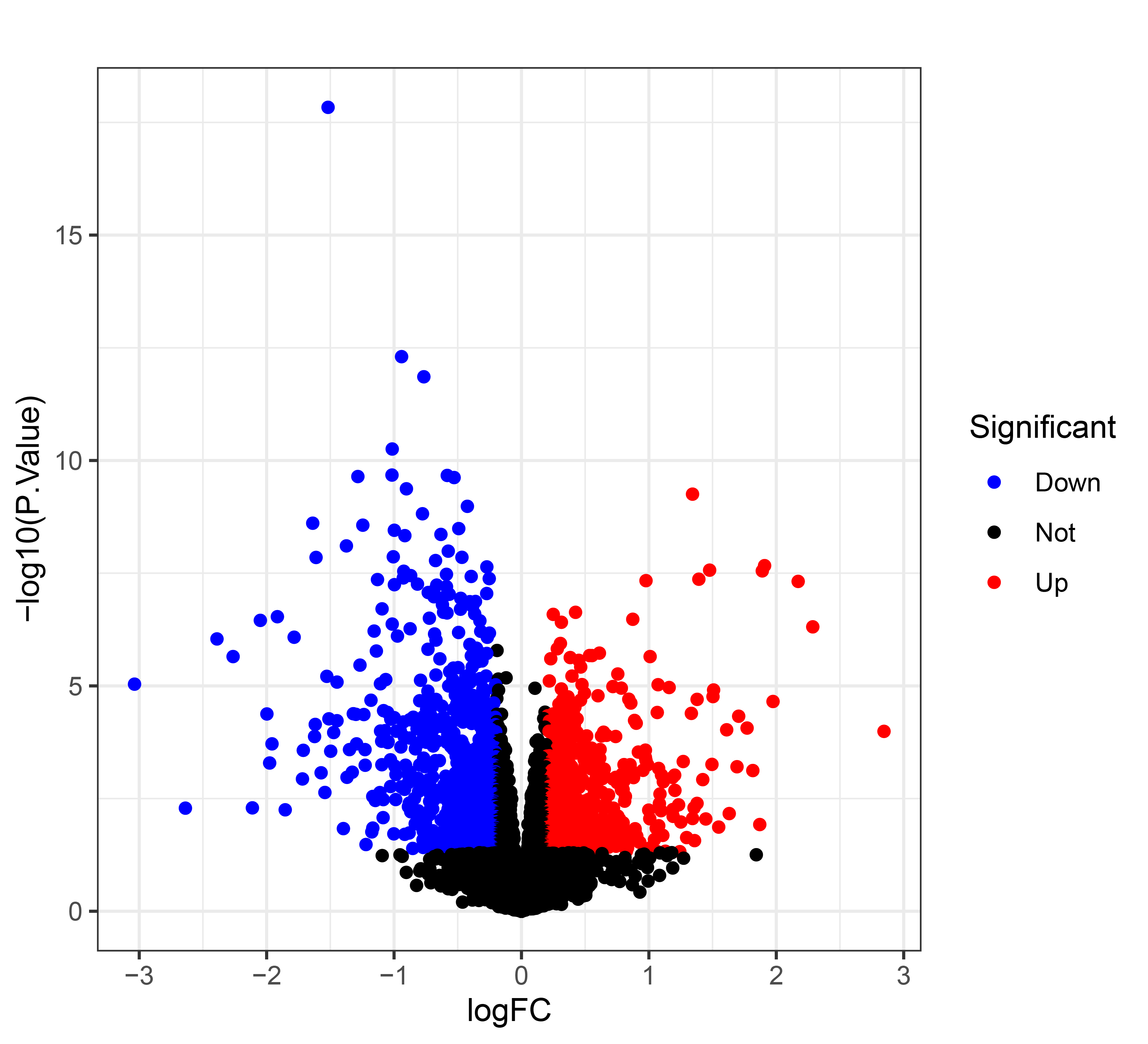

Supplement: Multimedia component 3 [file mmc3.zip › Single image/7C.tif]

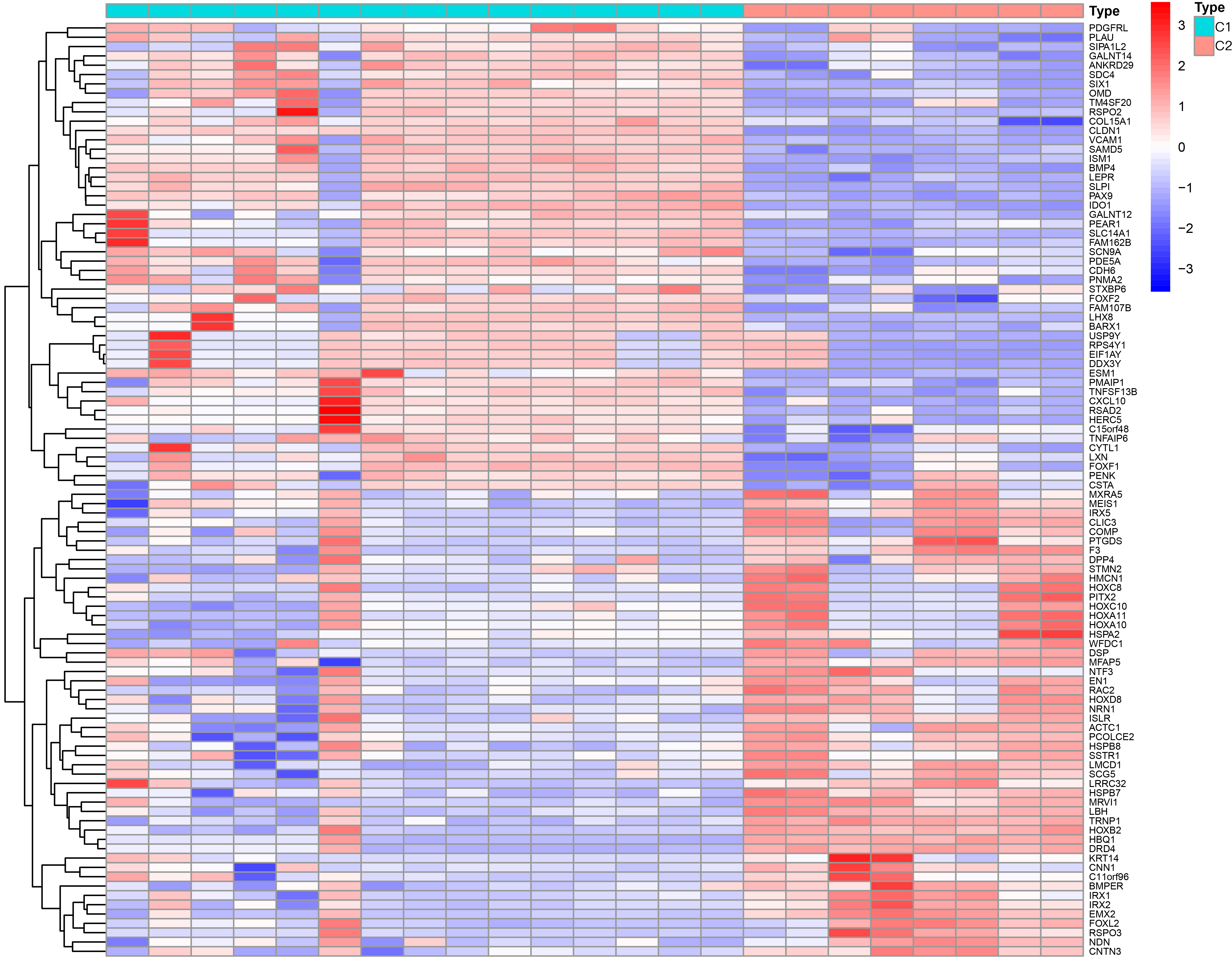

Supplement: Multimedia component 3 [file mmc3.zip › Single image/7D.tif]

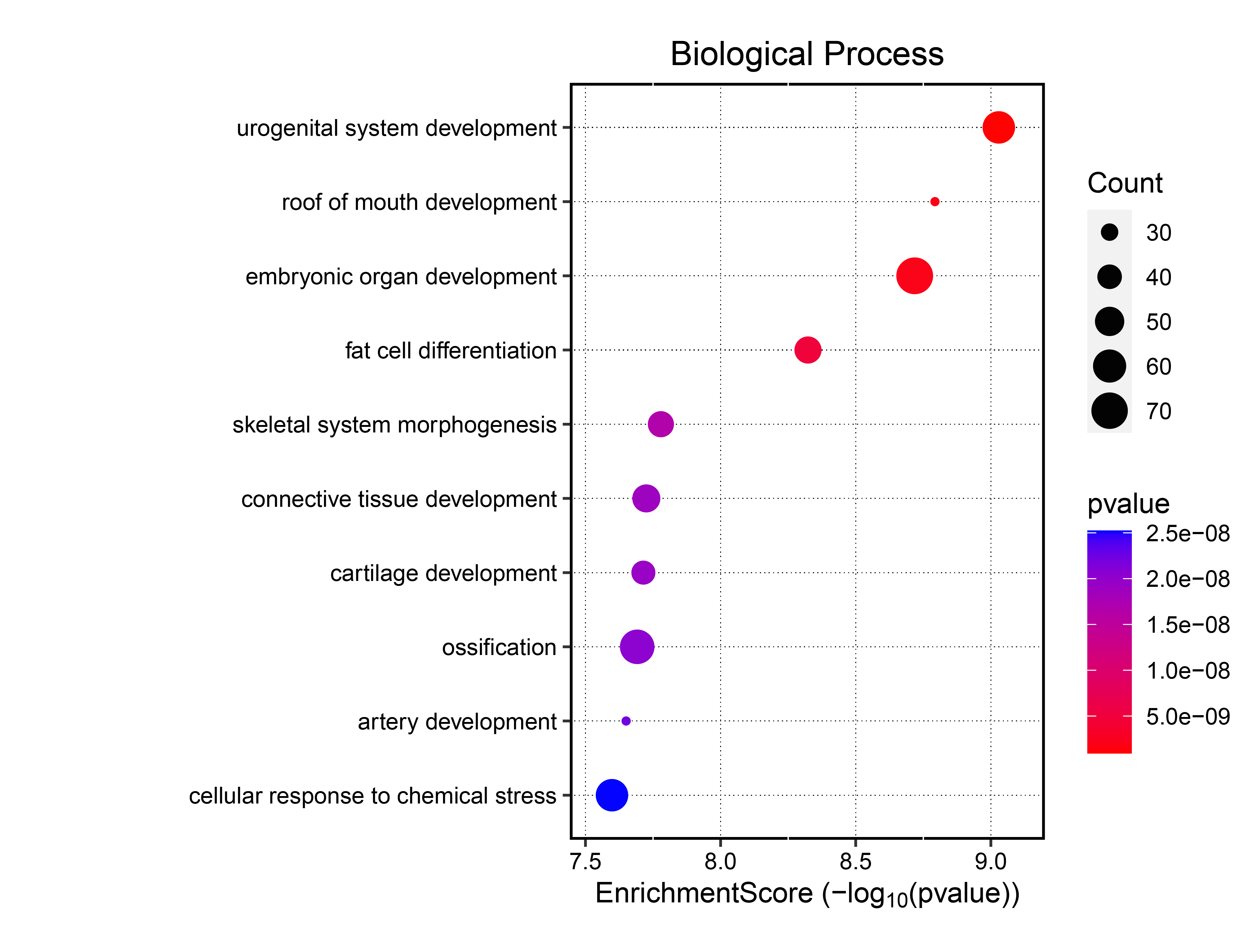

Supplement: Multimedia component 3 [file mmc3.zip › Single image/7E.tif]

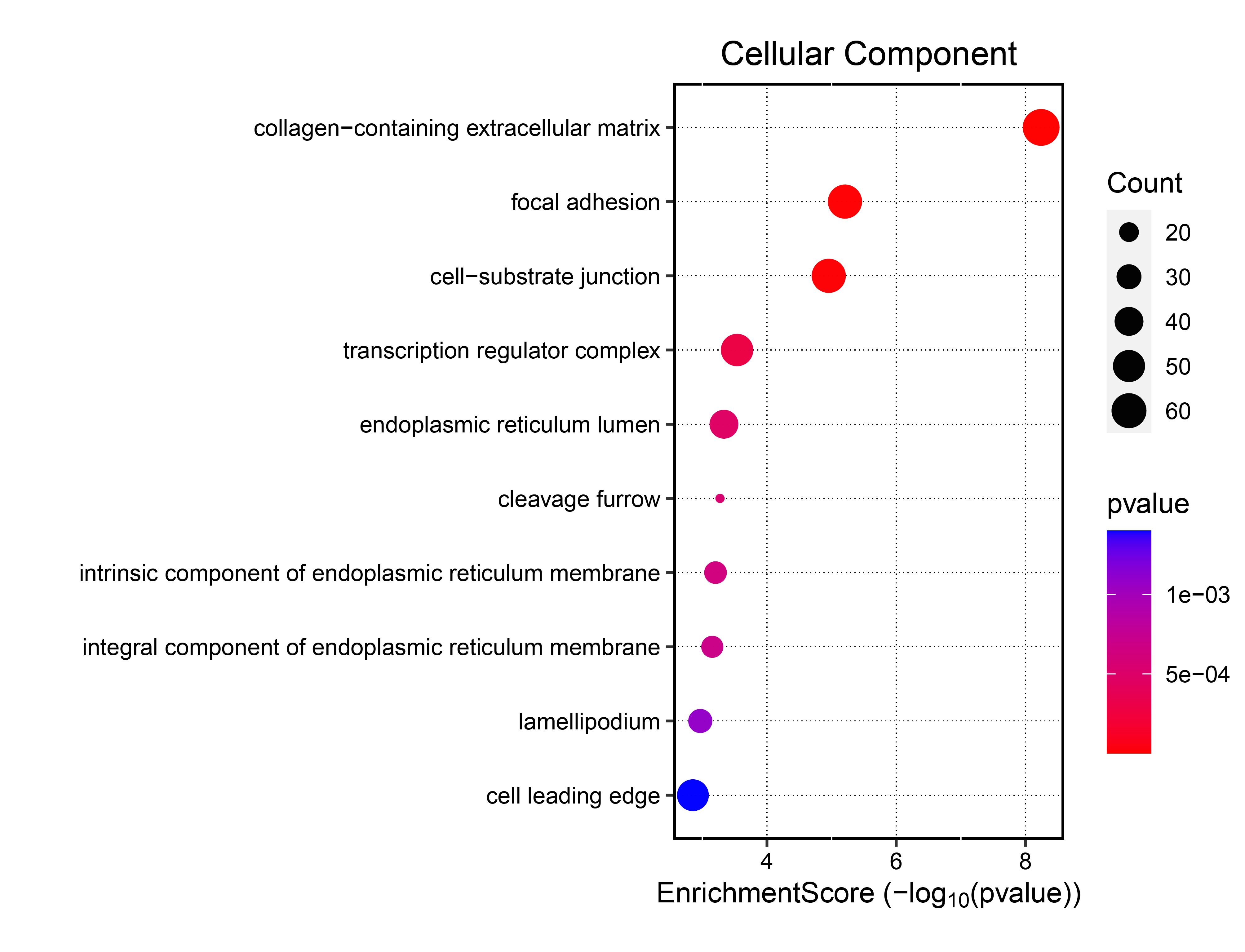

Supplement: Multimedia component 3 [file mmc3.zip › Single image/7F.tif]

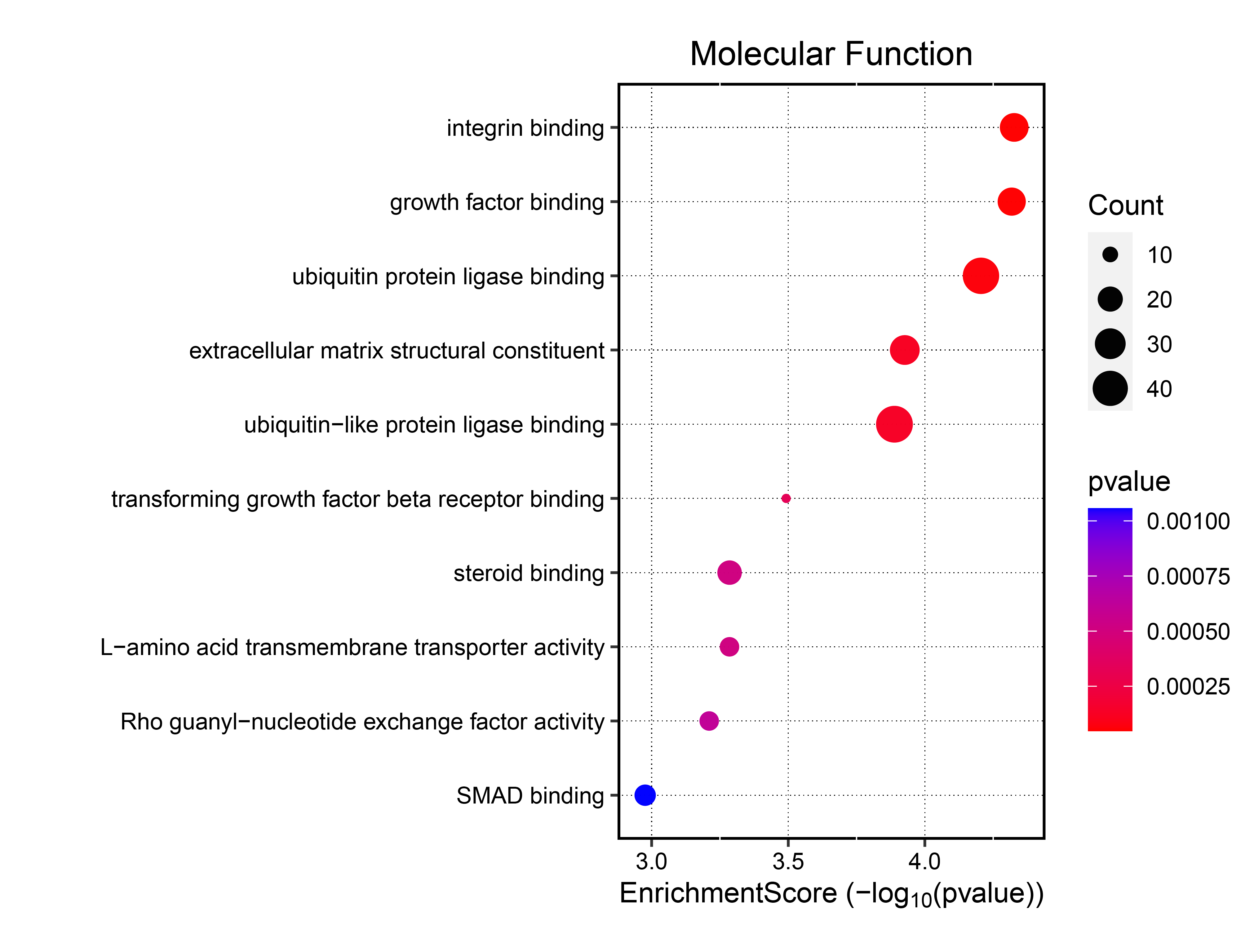

Supplement: Multimedia component 3 [file mmc3.zip › Single image/7G.tif]

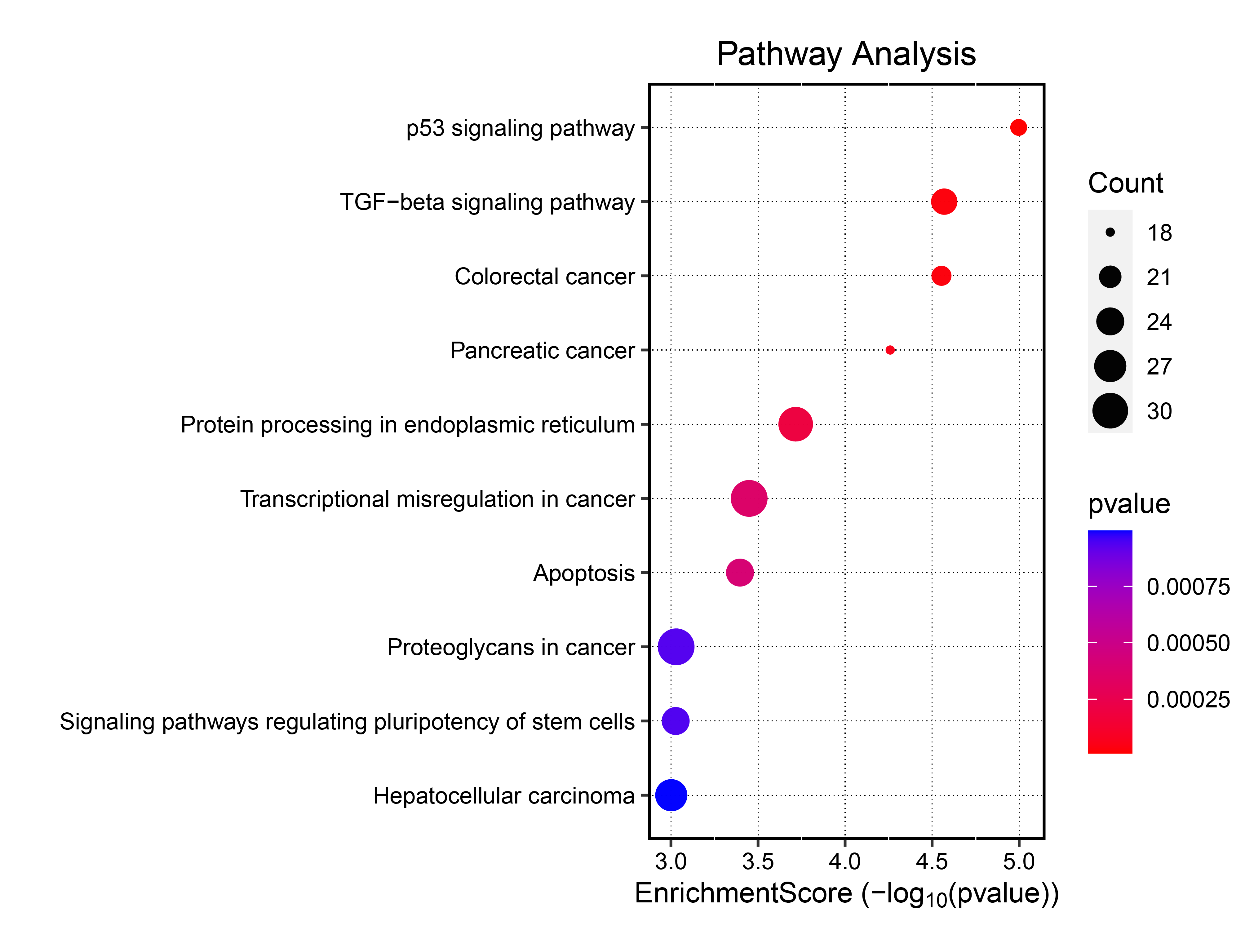

Supplement: Multimedia component 3 [file mmc3.zip › Single image/7H.tif]

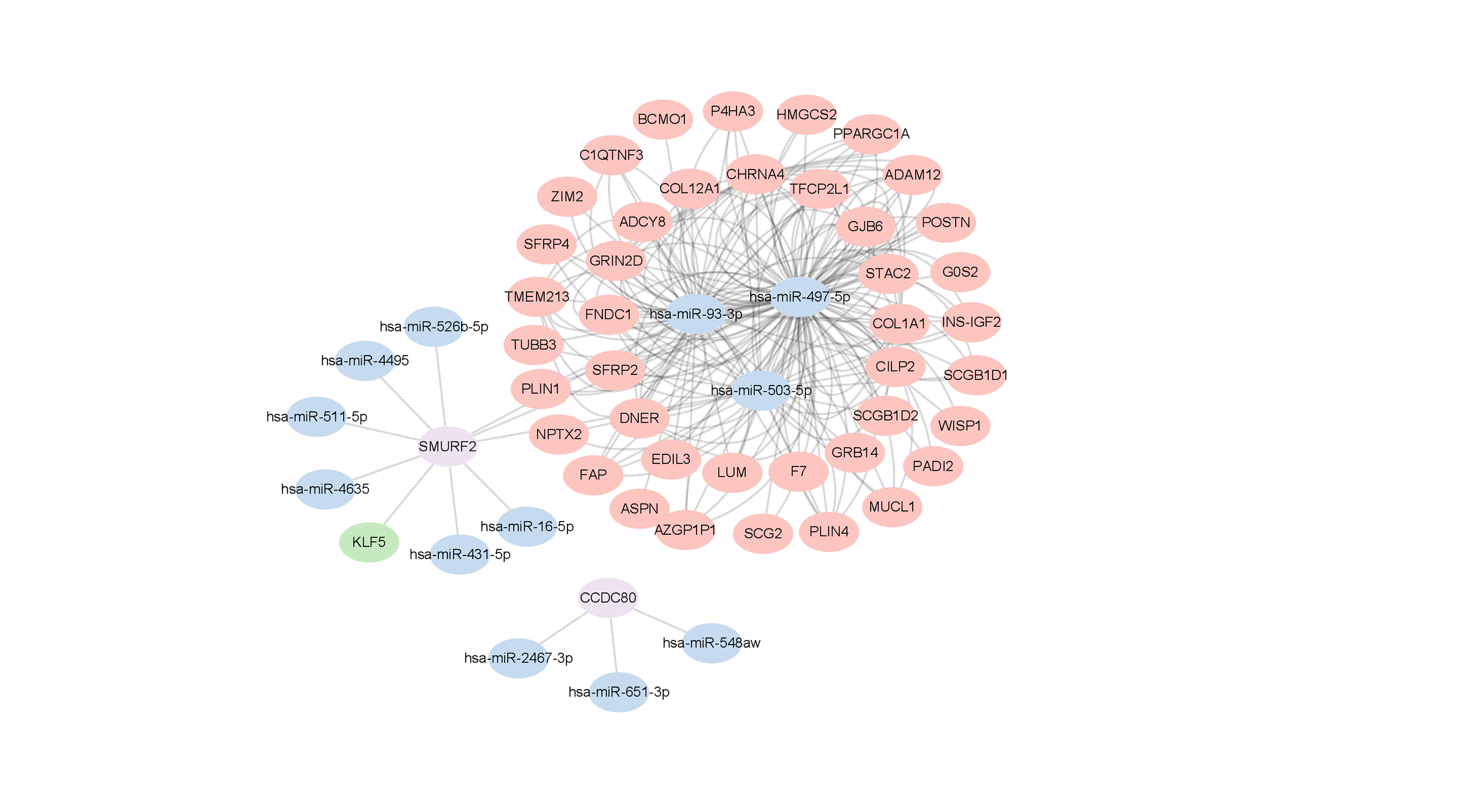

Supplement: Multimedia component 3 [file mmc3.zip › Single image/8A.tif]

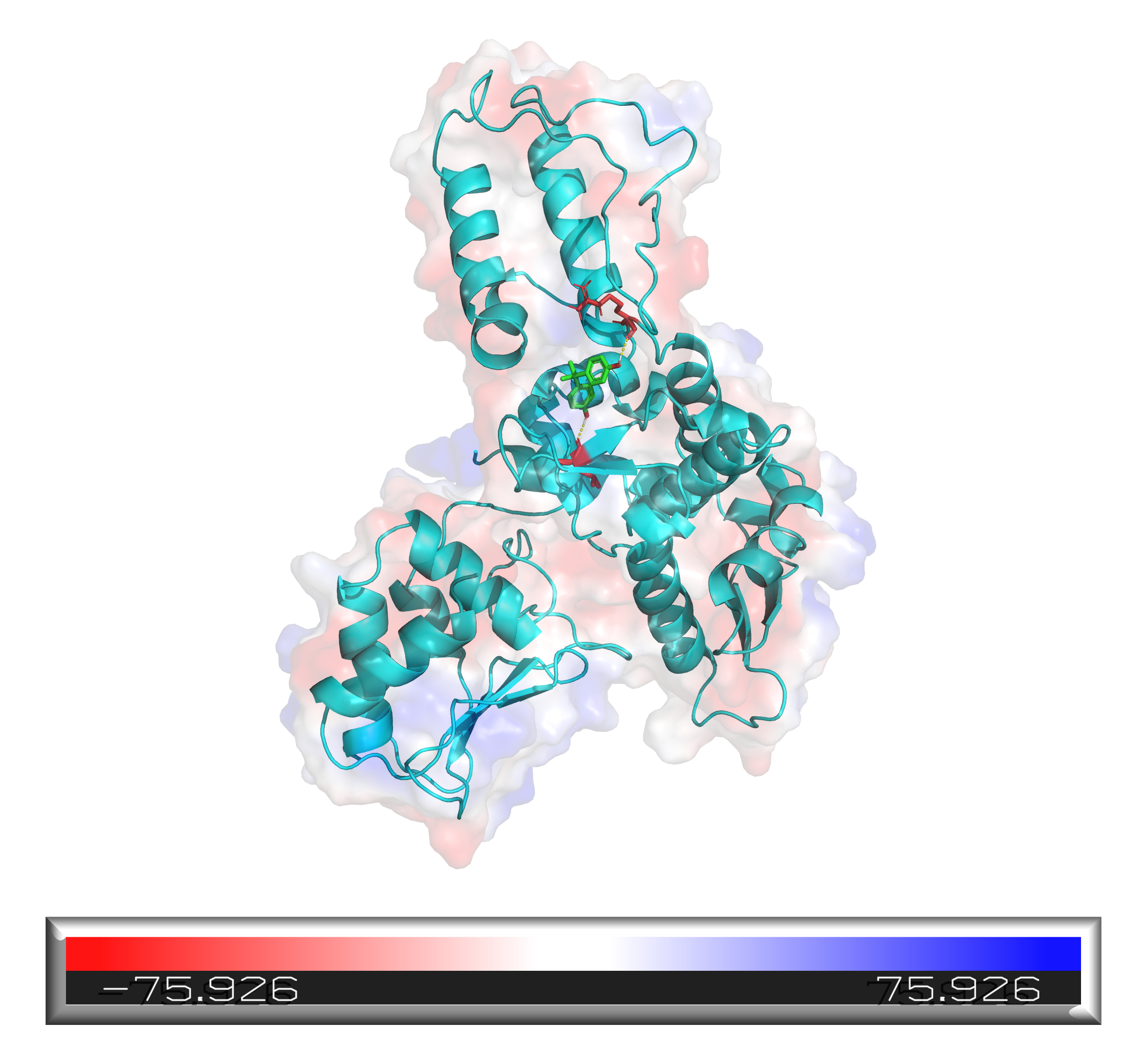

Supplement: Multimedia component 3 [file mmc3.zip › Single image/8B.tif]

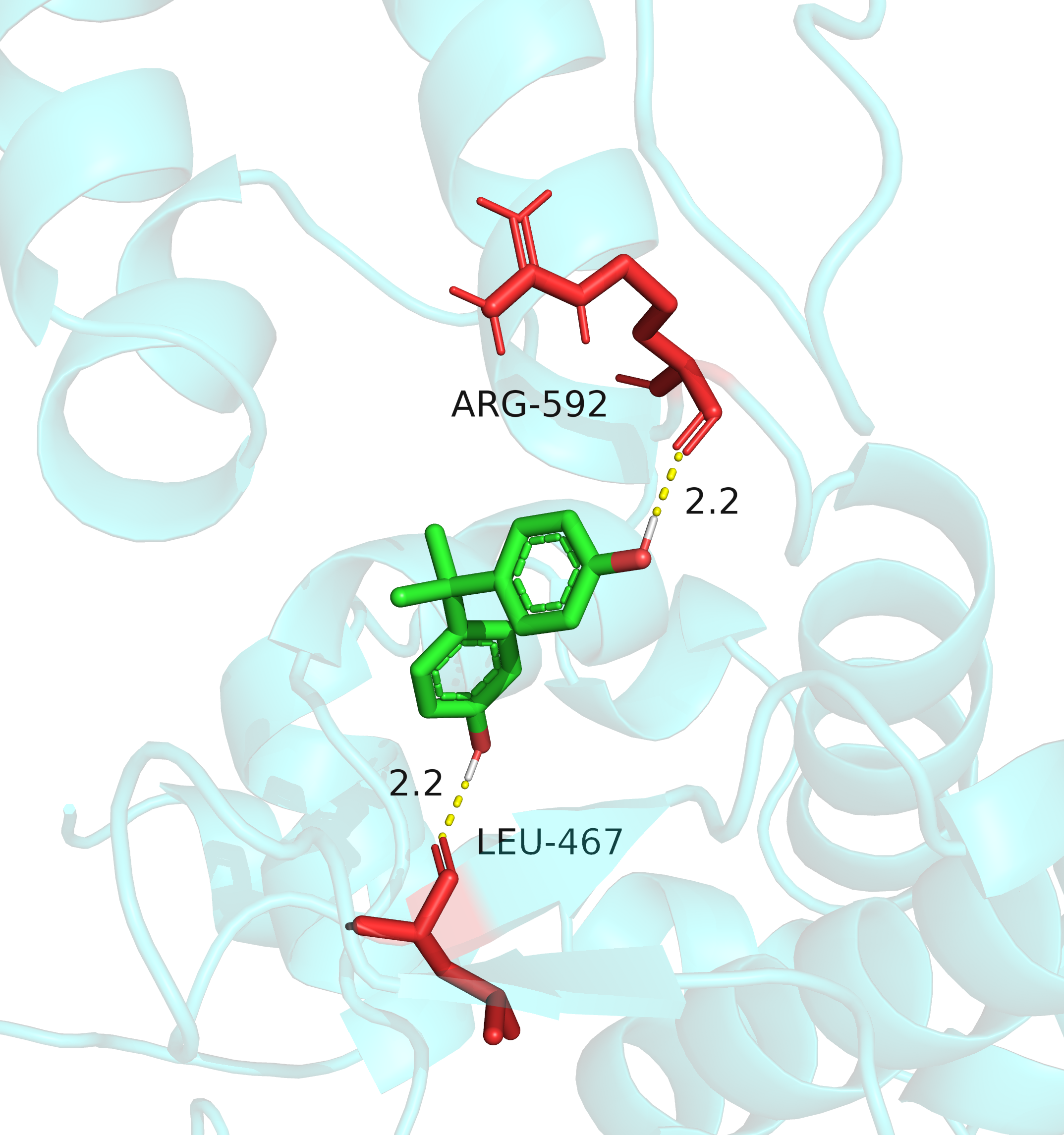

Supplement: Multimedia component 3 [file mmc3.zip › Single image/8C.tif]
